# Likely weakening of the Florida Current during the past century revealed by sea-level observations

**DOI:** 10.1038/s41467-020-17761-w

**Published:** 2020-08-07

**Authors:** Christopher G. Piecuch

**Affiliations:** Woods Hole Oceanographic Institution, 266 Woods Hole Road, Woods Hole, MA 02543 USA

**Keywords:** Physical oceanography, Physical oceanography

## Abstract

The Florida Current marks the beginning of the Gulf Stream at Florida Straits, and plays an important role in climate. Nearly continuous measurements of Florida Current transport are available at 27°N since 1982. These data are too short for assessing possible multidecadal or centennial trends. Here I reconstruct Florida Current transport during 1909–2018 using probabilistic methods and principles of ocean physics applied to the available transport data and longer coastal sea-level records. Florida Current transport likely declined steadily during the past century. Transport since 1982 has likely been weaker on average than during 1909–1981. The weakest decadal-mean transport in the last 110 y likely took place in the past two decades. Results corroborate hypotheses that the deep branch of the overturning circulation declined over the recent past, and support relationships observed in climate models between the overturning and surface western boundary current transports at multidecadal and longer timescales.

## Abstract (web summary)

Understanding trends in ocean circulation and dynamics is hampered by a lack of long-term records. Here the author uses probabilistic reanalysis of available data to show that transport by the Florida Current has declined over the past 110 years, indicating a slowdown of Atlantic Ocean circulation.

## Introduction

**Fig. 1:**
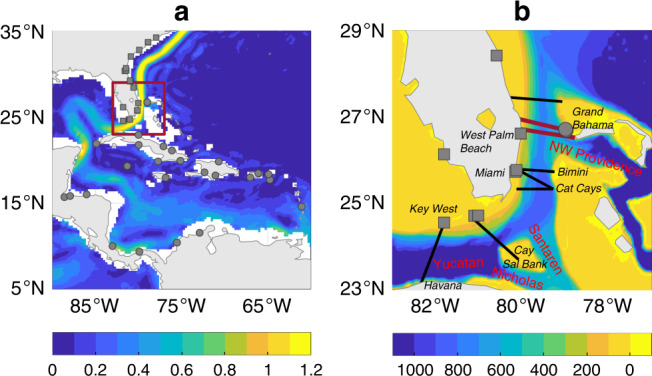
Florida Current and study region. **a** Gray squares (circles) are locations of tide gauges in the southeastern USA (Caribbean). Shading is mean ocean surface current speed (m s^−1^) from surface-drifter data^97^. Red box is area shown in **b**. **b** Details of Florida Straits. Shading is ocean depth (m). Red bold (black oblique) font indicates ocean channels (land locations) mentioned in the text. Thick red lines are locations of submarine-cable measurements^1–4^. Thin black lines are locations of in situ measurements from past studies^18–21^.

Swiftly flowing north through the narrow, shallow Florida Straits, the Florida Current marks the headwaters of the Gulf Stream^1–4^ (Fig. 1). Together with the weaker Antilles Current^5–8^, the Florida Current forms the major western boundary current in the subtropical North Atlantic Ocean at 27°N, providing closure to the wind-driven interior gyre circulation^9–11^, and acting as a vital limb of the Atlantic meridional overturning circulation^12^. Due to its transport of heat and other tracers, the Florida Current has an important role in climate^13–15^.

The integrated volume transport of the Florida Current, hereafter Florida Current transport, has been monitored nearly continuously at 27°N since 1982 through abandoned submarine telephone cables between West Palm Beach and Grand Bahama Island^1–4^ (Fig. 1). Before then, observations were made occasionally as part of short hydrographic cruises or brief field campaigns, each measuring a different component of the current at a distinct location. Earlier observations^16,17^ only measured near-surface transports, missing any transports at depth. Later full-depth transport measurements^18–22^ were made variously between Florida and Havana, Cay Sal Bank, the Cat Cays, or Bimini, which captured the flow through Yucatán Channel, but omitted transports through Nicholas, Santaren, or Northwest Providence Channels, all of which contribute to the transport at 27°N (Fig. 1). Such disparities make it difficult to produce a stable instrumental estimate of Florida Current transport through time. Without such a coherent, long-term estimate, it has been unclear whether the Florida Current has undergone multidecadal- or longer-timescale change. Meinen et al.^3^ concluded that the extant data do not support a long-term trend in the Florida Current transport during 1964–2009. However, it remains unclear whether a trend would emerge in a longer, more complete transport history.

Questions of possible long-term changes in the Florida Current bear on hypotheses that the Atlantic meridional overturning circulation has weakened or is weakening. Proxy indicators, including surface and subsurface ocean temperatures at subpolar latitudes and sortable silts from sediment cores off Cape Hatteras, suggest that the deep return flow of the meridional overturning circulation weakened either continuously during the twentieth century or earlier near the end of the Little Ice Age^23–27^. However, the proxies and their relation to the overturning are uncertain, so it is unclear how robust these suggestions are. Climate models simulate that weakening of the deep branch of the overturning circulation on multidecadal and longer timescales is balanced, in the sense of mass conservation, by weakening of the surface western boundary current^28–31^. Indeed, in paleoceanographic studies, changes in Florida Current transport have often been interpreted in terms of changes in the deep branch of the overturning circulation on centennial and millennial timescales^32^. Thus, a determination of whether the Florida Current transport changed during the past century, and by how much, based on instrumental observations would serve as a test of model simulations and proxy-based hypotheses regarding the deep branch of the overturning circulation, as well as inform paleoceanographic studies.

Previous authors argued that sea level from coastal tide gauges is informative of changes in Florida Current transport^17,18,33–37^. These arguments are based on geostrophic balance: at periods  ≳1 day, the northward flow through Florida Straits imparts an eastward acceleration due to the Coriolis force that is counteracted by a pressure gradient across the Florida Straits, which manifests as a sea-level difference that can be observed by tide gauges on opposite sides of the Florida Current. However, circulation inferences based on tide gauges need to be made cautiously. Tide gauges measure the distance between the sea surface and Earth’s crust at the coast. They capture not only the large-scale geostrophic circulation, but can also be impacted by remotely driven coastal-trapped waves and currents; local forcing and frictional dynamics over the continental shelf; changes in the gravity field, rotation vector, and viscoelastic deformation of the solid Earth; and other isostatic geophysical and oceanographic processes^38–40^. Tide-gauge data are also heterogeneously distributed in space and time. Long, continuous records are available at some southeastern USA and Caribbean sites far afield of the submarine cable at 27°N, but extant tide-gauge records close to the cable’s endpoints near West Palm Beach and Grand Bahama are short, incomplete, and largely not overlapping^41^ (Supplementary Figs. 1, 2).

To overcome these challenges, I use Bayesian data analysis^42–44^ to estimate annual Florida Current transport at 27°N during the past 110 y (see “Methods” section). The estimate uses 1390 y of annual coastal sea level from 46 tide gauges^41^ along the southeastern USA and Caribbean during 1909–2018 and 36 y of annual Florida Current transports from cable measurements^1–4^ over 1982–2018. Sea level is represented as a process with spatial correlation and temporal memory. The Florida Current transport is related to the difference in sea level across Florida Straits through geostrophy, but account is taken of non-oceanographic and ageostrophic effects on sea level and transport. The data are modeled as corrupt, imperfect versions of the processes. Bayes’ rule is used to invert the model equations, and solutions are generated using numerical methods. The model equations are coupled, so that information is shared across space, time, and processes, which allows data gaps to be filled and unobserved processes to be estimated. The solution is fully probabilistic, and comprises thousands of ensemble members, each an equally likely history of transport that is consistent with the data and model equations. This allows subtle spatiotemporal statistics to be calculated, for example, the probability density function of the magnitude or timing of the minimum or maximum decadally averaged transport value during the study period (see “Methods” section). Residual analyses and synthetic data experiments demonstrate the appropriateness of the algorithm and show that it accurately infers the quantities of interest given the data (see “Methods” section).

## Results

### Weakening of the Florida Current

**Fig. 2:**
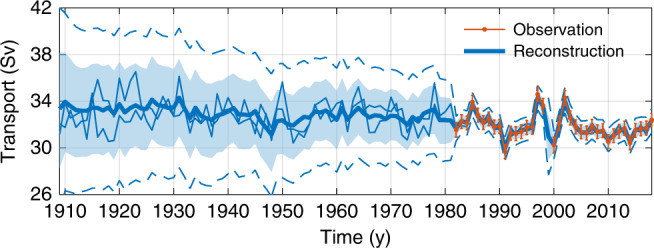
Florida Current transport. Blue shows posterior median (thick line), 95% pointwise (light shading), and pathwise (dash dot) credible intervals, along with two arbitrary, randomly selected ensemble members (thin lines) from the probabilistic Florida Current transport solution. Orange shows annual transport from raw submarine-cable-data plus and minus twice the standard error estimated following refs. ^3,4^.

The probabilistic reconstruction of Florida Current transport is summarized in Fig. 2. The 110-y mean transport is 32.6 ± 1.4 Sv (Supplementary Fig. 3a; 1 Sv ≡ 10^6^ m^3^ s^−1^). Unless otherwise indicated,  ±  values represent the median plus and minus twice the standard deviation inferred by the Bayesian model, which roughly corresponds to the 95% posterior credible interval. The average transport since 1982, when transport has been continuously monitored, is 31.8 ± 0.1 Sv. This value is likely weaker (probability *P* = 0.86) than the average transport during 1909–1981 (32.9 ± 2.1 Sv) before continuous monitoring of the Florida Current (Supplementary Fig. 3a). Estimated uncertainties since 1982 are relatively small, and mostly reflect instrumental errors on the cable data, which strongly constrain the posterior solutions of the transport process. Before 1982, cable data are unavailable at 27°N, and the inference is largely constrained by the tide-gauge records, which have a more uncertain relationship to transport and become sparser earlier in time, resulting in comparatively larger errors that grow into the past.

Superimposed on the time mean are interannual-to-decadal fluctuations in transport (Fig. 2). The standard deviation of annual transports is 1.3 Sv (posterior median estimate). A  −3.3 ± 1.1 Sv weakening from 1997–1998 to 1999–2000, when there was a gap in cable data and low transports were observed upstream in Yucatán Channel^45^, was followed by a 2.5 ± 1.1 Sv strengthening between 1999–2000 and 2001–2002 (Supplementary Fig. 3c). Decadal-average transports during 1922–1932 (33.6 ± 2.8 Sv) and 1956–1966 (33.0 ± 1.7 Sv) were likely (*P* ≥ 0.79) higher than the long-term 110-y mean transport, whereas decadal-mean transports over 1946–1956 (32.2 ± 2.0 Sv) and 1986–1996 (31.7 ± 0.2 Sv) were likely lower than the long-term mean (Supplementary Fig. 3d). Wavelet coherence analysis reveals that transport fluctuations can be related to major modes of surface climate variation (Supplementary Fig. 4). Transport is probably (*P* > 0.68) coherent with the North Atlantic Oscillation^46^ over 2–8-y periods centered between the late 1970s and early 2000s, consistent with past studies of the cable data^2,47^. Coherence is also evident at 2–4-y periods around 1960, and 8-y periods between the late 1930s and early 1950s, which have not been reported previously, and possibly result from changes in forcing by wind-stress curl over the subtropics and mediated by planetary Rossby waves^47^. Transport is also likely (*P* > 0.68) coherent with Atlantic Multidecadal Variability^48^ at 2–16-y periods centered on the mid 1990s and 16-y periods from the late 1940s to early 2000s. Weaker coherence earlier in time could reflect nonstationary relationships between transport and climate or the growth of transport uncertainties into the past.

**Fig. 3:**
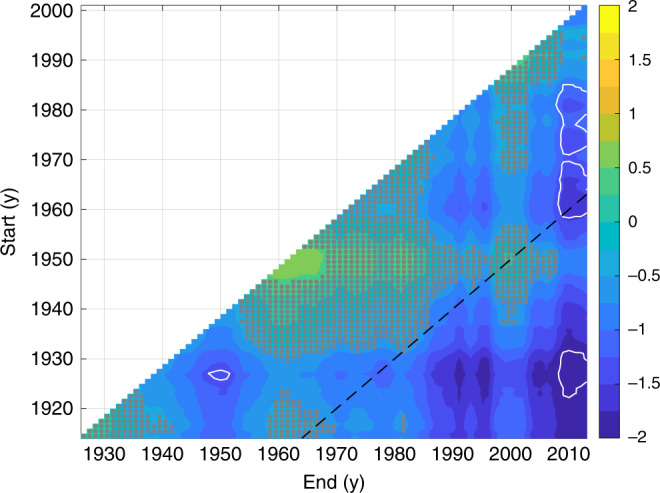
Weakening of Florida Current transport over different periods. Shading shows posterior median estimates of the change in decadal-average Florida Current transport between all pairs of decades (Sv). Negative values indicate that transport fell between the start and the end of the period. Stippling indicates that it is as likely as not (0.33 < *P* < 0.67) that transport rose or fell. White (black) contours encircle periods when it is very likely (*P* > 0.90) that transport weakened (strengthened) from the start to the end decade more than expected from a stationary red-noise process; see “Methods” section for discussion of significance calculations. Black dashes mark  >50-y periods (values below and to the right of the line correspond to periods with duration  >50 y).

**Fig. 4:**
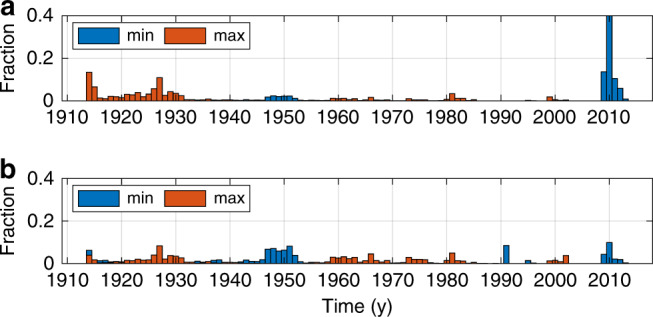
Timing of Florida Current decadal transport extrema. **a** Histograms of modeled probabilities that the minimum (blue) and maximum (orange) decadal-average transport occurred centered on a given year. **b** As in **a** but histograms were calculated after removing the corresponding long-term trend. See “Methods” section for discussion of statistics and uncertainty measures.

Weakening of the Florida Current transport is apparent on longer timescales. The centennial trend during 1909–2018 is  −1.7 ± 3.7 Sv century^−1^, which overlaps zero, but implies that the trend is likely negative (*P* = 0.82; Supplementary Fig. 3b). The inference of a long-term weakening is qualitatively insensitive to the choice of time period. Computing changes between all pairs of non-overlapping decadal averages, I find that transport likely (*P* > 0.68) declined from one decade to another 65% of the time (i.e., 65% of pixels in Fig. 3 are blue and not stippled). Considering only changes over  >50-y intervals, I find that the percentage of periods showing a likely decline increases to 92% (Fig. 3). For example, it is very likely (*P* = 0.90) that transport weakened from 1920–1930 (−2.1 ± 2.9 Sv) and from 1960–1970 (−1.4 ± 1.6 Sv) to the present more than expected from a stationary red-noise process. Indeed, if transport was stationary, extrema would be uniformly likely to occur at any point on a given time interval, whereas in the presence of a long-term decline, the maximum transport would be more likely to happen closer to the beginning, and the minimum transport closer to the end of the interval. Consistent with the latter case, the minimum decadal-average transport (31.1 ± 1.0 Sv) likely started sometime after 2002 (*P* = 0.74), whereas the maximum decadal average (34.1 ± 2.5 Sv) probably ended before 1936 (*P* = 0.70; Fig. 4a). The timing of these extrema cannot be explained in terms of fluctuations about a stationary mean: after removing the long-term trend (Supplementary Fig. 3b), I find it unlikely that the minimum transport started after 2002 (*P* = 0.18), and chances are lower than the maximum ended before 1936 (*P* = 0.38; Fig. 4b).

### Relation between transport and sea level

**Fig. 5:**
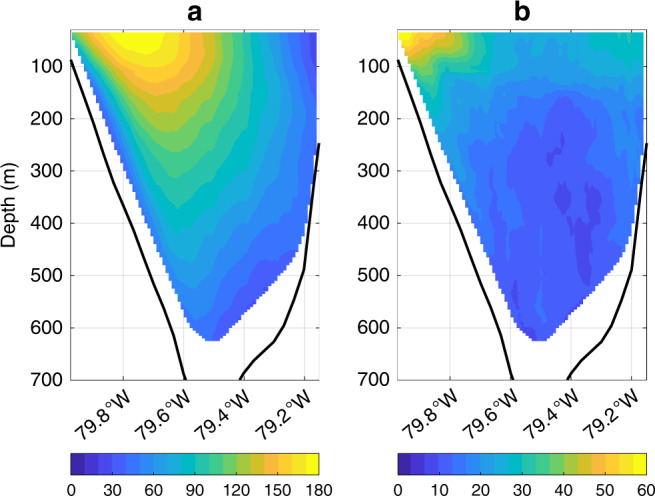
Structure of the Florida Current within Florida Straits. **a** Mean northward velocities (m s^−1^) through Florida Straits from shipboard acoustic doppler current profiler data from 70 research cruises of the research vessel Walton Smith between 2001 and 2018. Values are computed by interpolating all data between 26. 9°N and 27. 1°N from a given cruise onto a common grid using a linear scattered data interpolant, and then averaging over all cruises. For a value to be shown at a longitude and depth, data must have been available from at least 14 cruises. **b** As in **a** but showing the standard deviation in meridional velocities (m s^−1^) across cruises. Black lines mark the bathymetry.

In addition to transport, the Bayesian algorithm also estimates the regression coefficient between transport and sea-level difference across Florida Straits (see Methods). This estimated change in transport per unit change in sea-level difference is 0.21 ± 0.11 Sv cm^−1^ (Supplementary Fig. 5a). I use geostrophy to interpret this value in terms of an effective depth characterizing the vertical scale over which velocity variations decay in amplitude from the surface to the bottom in Florida Straits^49,50^. Following Little et al.^50^, I multiply by the ratio of Coriolis parameter over gravity (6.7 × 10^−6^ s m^−1^ at 27^∘^N) to obtain an effective depth of 144 ± 74 m. This estimate is roughly consistent with the vertical structure of northward currents observed by shipboard acoustic doppler current profiler aboard the research vessel Walton Smith during 70 cruises across Florida Straits at 27°N over 2001–2018. At the longitude of the core of the current, the average meridional velocity over all cruises decays almost linearly in the vertical from  ~1.2 m s^−1^ near the surface to  ~0.9 and  ~0.6 m s^−1^ at 200- and 400-m depth, respectively (Fig. 5a). Computing standard deviations in meridional velocities across cruises, I find that the decay in flow-variation amplitude with depth takes a more exponential form, decreasing rapidly from  ~0.6m s^−1^ near the surface to ~0.3 and  ~0.2 m s^−1^ at 200- and 400-m depth, respectively (Fig. 5b). Similar vertical structures of mean and variable meridional currents were reported from earlier observations made during 1982–1984 as part of the Subtropical Atlantic Climate Studies Program^51^.

In designing the Bayesian algorithm, I assumed that the regression coefficient between sea level and transport is time invariant (see “Methods” section). To test whether this assumption is reasonable, I analyze sea level and transport simulated by an ocean circulation model^52^ over 1871–2010 in the time and frequency domains. Performing admittance and coherence analyses^53^, I determine that modeled sea-level differences across Florida Straits and Florida Current transports are coherent over all accessible timescales, and that the admittance (or transfer function) amplitude is relatively insensitive to frequency band, such that the change in transport per unit change in sea-level difference is similar at interannual and multidecadal timescales (Supplementary Fig. 6). I also perform correlation and regression analyses on the simulated sea-level differences and transports using a sliding 20-y time window. Correlation and regression coefficients are relatively robust to the choice of time period, such that values for any 20-y epoch are within about 20% and 5%, respectively, of the average value over all 20-y epochs over 1871–2010 (Supplementary Fig. 7). These results show that the link between transport and sea-level difference in this ocean model is not strongly dependent on time period or frequency band, which suggests that my assumption of a constant-in-time regression coefficient between the two variables is justifiable. The Bayesian algorithm also successfully infers the correct regression-coefficient value in a synthetic data experiment based on this ocean circulation model (see “Methods” section; Supplementary Figs. 6 and 7), which means that the algorithm correctly estimates the relationship between sea level and transport given the available (gappy, noisy) data.

### Distinguishing dynamic and static sea-level trends

The meaningfulness of the transport estimate hinges on the Bayesian algorithm’s ability to identify and distinguish dynamic from static components of the sea-level difference across the Florida Straits, given the available data. Here dynamic indicates ocean dynamic sea level, which is the local sea-surface height relative to the geoid and corrected for the inverted-barometer effect, whereas static refers to relative sea-level changes unrelated to ocean circulation, including global-mean sea-level changes, the inverted-barometer effect, and sea-level changes arising from changes in Earth’s gravity, rotation, and viscoelastic solid-Earth deformation^54^. The posterior solution for the 110-y trend in sea-level difference across the Florida Straits, between Grand Bahama minus West Palm Beach, is  −0.2 ± 1.0 mm y^−1^ (Supplementary Fig. 5b). This trend results from the competing influences of a dynamic trend in sea-level difference of  −0.9 ± 2.2 mm y^−1^ and a static trend of 0.7 ± 2.3 mm y^−1^ (Supplementary Fig. 5b), which I interpret respectively as differential trends in sea-surface height and vertical land motion across Florida Straits.

Several lines of independent evidence corroborate these inferences, and support my interpretation in terms of sea-surface height and vertical land motion. The Global Positioning System (GPS) provides instrumental observations of vertical land motion. Version 6b of the data set from Université de la Rochelle^55^ gives continuous GPS records from three locations in southeastern Florida and two Bahamas locations (Supplementary Fig. 8 and Supplementary Table 1). I compute the average vertical velocity for the two Bahamas sites, and do the same for the three sites in southeastern Florida. Taking the difference between the two averages, I find that sea level is statically rising 1.0 ± 1.3 mm y^−1^ faster in the Bahamas than on southeastern Florida due to differential land subsidence. Here the  ±  value is the best-estimate plus and minus twice the estimated standard error, assuming that the standard errors provided with the GPS data are independent (Supplementary Table 1). This rate of sea-level rise agrees with the static trend in the sea-level difference across Florida Straits inferred by the Bayesian model.

Proxy sea-level reconstructions are informative of background rates of change unrelated to ocean dynamics. I consider recent standardized compilations of Holocene sea-level index points from the Caribbean and southeastern USA derived from coral reefs, mangrove peats, and other indicators^56,57^. To estimate present-day rates of background change unrelated to circulation and climate, I only consider locations in the databases that have at least three sea-level index points with best-estimate ages between 2000 and 150 y before present. This criterion is satisfied by two southeastern Florida sites and one Bahamas site (Supplementary Fig. 8; Supplementary Table 2). Taking the difference between the linear trend fit to the index points from the Bahamas site and the average of the trends fit to the data at the two southeastern Florida locations, I estimate that sea level rose 0.6 ± 0.6 mm y^−1^ more rapidly in the Bahamas relative to southeastern Florida in the pre-industrial Common Era (Supplementary Table 2). Here the  ±  value is the best-estimate plus and minus twice the standard error from ordinary least squares applied to the best estimates of proxy age and sea level, assuming white-noise residuals. Interpreted in terms of vertical land motion, this sea-level trend difference from proxy data suggests that the difference in rates of vertical land motion between the Bahamas and southeastern Florida observed by GPS is, at least partly, due to background geological processes (e.g., glacial isostatic adjustment).

Modern radar altimeters have observed sea-surface height over nearly the global ocean since 1993. Once adjusted for static effects, altimeter data can be interpreted in terms of surface currents and ocean dynamics. I consider along-track sea-surface height from the Centre for Topography of the Oceans and the Hydrosphere^58^ at the altimeter data points closest to Settlement Point on Grand Bahama Island and Virginia Key in southeastern Florida (Supplementary Fig. 8). Differencing the two altimetric time series and fitting a linear trend, I determine that the rate of change in sea-surface-height difference across Florida Straits during 1993–2017 was  −2.2 ± 3.0 mm y^−1^ (Supplementary Fig. 9). Here  ±  is the best-estimate plus and minus twice the estimated standard error based on simulations with synthetic time series, with the same Fourier amplitude as the data but random phase, to account for autocorrelation in the residuals^59,60^ (Supplementary Note 2). This altimetric trend, while covering a relatively short interval, basically agrees in sign and magnitude with the dynamic trend in sea-level difference across Florida Straits from the Bayesian model. Note that, while closer to the western end point of the submarine cable than Virginia Key, the West Palm Beach tide gauge is not considered. Due to the geometry of the satellite tracks, the closest altimeter point to West Palm Beach is about 50 km offshore, east of the current core, and does not reflect western boundary sea level (Fig. 5; Supplementary Fig. 8).

### Relation to large-scale North Atlantic Ocean circulation

Assuming there were no changes in Bering Strait throughflow, or evaporation and precipitation over the basin, mass conservation demands that weakening of the Florida Current transport must be balanced by equal and opposite changes in transports by other components of the circulation at 27°N. This mass-conservation requirement can be met by weakening of the southward-flowing interior gyre, strengthening of the northward Antilles Current, weakening of the southward deep return flow of the overturning, or some combination thereof.

**Fig. 6:**
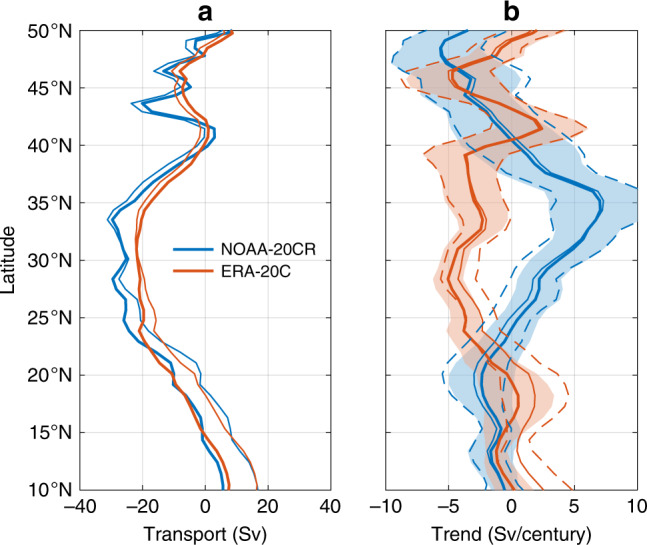
Changes in wind-stress curl and gyre circulation. **a** Thick lines are time-mean geostrophic Sverdrup streamfunction^9^ based on wind-stress curl from NOAA 20CR^61^ and ERA 20C^62^ reanalyses over 1900–2010 as a function of latitude in the North Atlantic. Thin lines are the same, but also incorporate the ageostrophic Ekman transport integrated across the basin. **b** Median estimates (thick lines) and formal 95% confidence intervals (colored shading) of the trend in Sverdrup streamfunction versus latitude during 1900–2010 from the two reanalyses. Thin and dashed lines represent median estimates and confidence intervals, respectively, with Ekman transports also included.

To consider possible changes in wind-driven interior gyre transports, I compute geostrophic Sverdrup streamfunction^9^ using wind-stress curl from two reanalyses for the twentieth century^61,62^. I find a mean southward transport of between  −21 and  −25 Sv at 27°N for 1900–2010 (Fig. 6a). Note that negative (positive) values correspond to southward (northward) transports. This agrees with historical and modern estimates of the mean interior geostrophic gyre transport at 27°N from gridded wind products^5,11,63–65^ and hydrographic observations^66^ for different time periods, which range from  −16 to  −27 Sv. However, the two reanalyses give conflicting estimates of long-term trends in Sverdrup transport (Fig. 6b; Supplementary Fig. 10). For 1900–2010, one reanalysis^61^ gives a weaker northward trend (1.9 ± 2.0 Sv century^−1^), while the other^62^ produces a stronger southward trend (−4.2 ± 1.3 Sv century^−1^) at 27°N (Fig. 6b). Here  ±  is the best-estimate plus and minus twice the standard error based on synthetic data simulations to account for residual autocorrelation^59,60^ (Supplementary Note 2). Discrepancies in Sverdrup transport trends are apparent broadly over the subtropics. One reanalysis^62^ produces significant negative trends suggesting spin-up of the gyre, while the other^61^ shows significant positive trends indicating gyre spin-down over 1900–2010 (Fig. 6b). Such discrepancies are evident more generally at multidecadal and centennial periods. Considering all periods starting between 1900 and 1980 and ending in 2010, I consistently find significant trends in the residual difference in Sverdrup transport between reanalyses at 27°N (Supplementary Fig. 10). These findings are unchanged if ageostrophic Ekman transports are also considered (Fig. 6b; Supplementary Fig. 10). Thus, while they do not paint a consistent portrait of whether the interior gyre strengthened or weakened over the past century, reanalyses suggest that long-term trends in gyre transports of several Sv century^−1^ are possible.

The Antilles Current is a subsurface western boundary current constrained to the upper slope east of Abaco at 26.5°N. Data from the RAPID array since 2004 show that the Antilles Current has a mean northward transport of between 1 and 6 Sv^6–8,66^. While weaker in a time-average sense, the Antilles Current transport is as variable as, if not more variable than, the Florida Current transport^7,8^. Variation in Antilles Current transport has been attributed to a combination of westward-propagating eddies and large-scale wind forcing associated with the Bermuda High^5,7^. Continuous measurements of Antilles Current transport during 1986–1991 and since 2004^5,8^ are too short for diagnosing long-term trends. However, one can put bounds on stochastic trends based on the time-series properties of the available data. Performing simulations of a random stationary process, with the same integral timescale and variance as the observed Antilles Current transport over 2005–2015^8^, I find that stochastic transport trends of  ±2.9 and  ±1.2 Sv century^−1^ are possible on 50- and 100-y timescales, respectively (Supplementary Note 3 and Supplementary Fig. 11). Here  ±  values are 95% confidence intervals determined from the simulations. These results imply that multidecadal and centennial trends in Antilles Current transport, on the same order of magnitude as the trends in Florida Current transport estimated here, cannot be ruled out.

**Fig. 7:**
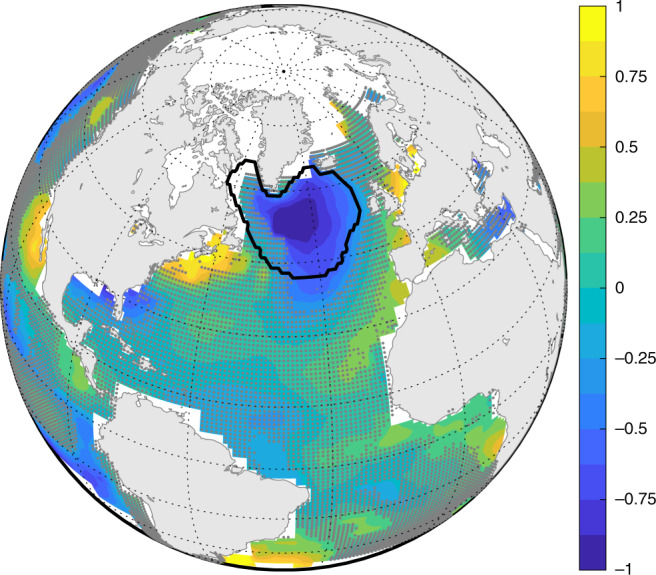
Changes in sea-surface temperature. Shading shows trends in sea-surface temperature over 1909–2018 (^∘^C century^−1^) averaged over two products: HadISST^69^ and Kaplan^70^. Stippling indicates that the magnitude of the trend is less than twice the standard error estimated taking into account autocorrelation of the residuals^59,60^ as described in the Supplementary Information. The black contour outlines the warming-hole region of Caesar et al.^24^. Note that the time series of global-ocean area-averaged sea-surface temperatures has been removed before computing trends.

**Fig. 8:**
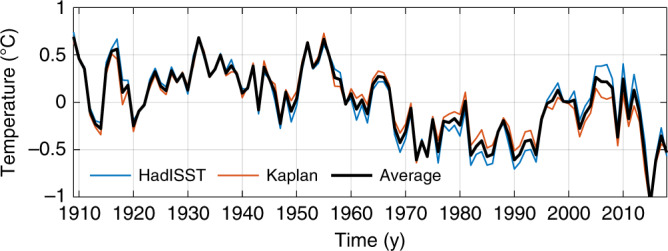
Sea-surface-temperature changes over the warming hole. Time series of anomalous sea-surface temperature (°C; time-mean value removed) averaged over the warming-hole region of Caesar et al.^24^ (see black contouring in Fig. 7) from HadISST^69^ (blue), Kaplan^70^ (orange), and the average between the two products (black). Note that the time series of global-ocean area-averaged sea-surface temperatures has been removed.

Previous studies argued that the deep branch of the overturning circulation declined in the recent past^23–27^. These arguments were partly based on: (1) strong correlation between overturning streamfunction and subpolar North Atlantic sea-surface temperature on multidecadal and longer timescales in climate models^23,24,67,68^; and (2) observations^69,70^ of a warming hole in the subpolar North Atlantic^23,24^, where sea-surface temperature cooled by  −0.6 ± 0.4 °C century^−1^ relative to the global average during 1909–2018 (Figs. 7 and 8). Here  ±  is the best-estimate plus and minus twice the standard error accounting for residual autocorrelation^59,60^ (Supplementary Note 2). However, it has been unclear what processes mediate links between the overturning and subpolar sea-surface temperature in models^71,72^. All else being equal, the trend in sea-surface temperature implies a trend in surface heat flux and ocean heat uptake of 16 ± 11 W m^−2^ century^−1^ over the warming hole (Supplementary Note 4). If this heat was stored locally in the North Atlantic and Arctic Ocean, from 27°N to Bering Strait, it would manifest in an average temperature acceleration of 0.7 ± 0.5 °C century^−2^ over the entire water column, or a full-depth warming of 0.4 ± 0.3 °C during 1909–2018 (Supplementary Note 5). This is larger than published estimates of northern North Atlantic warming over the past century^73,74^. For example, the model-data synthesis of Gebbie and Huybers^73^ suggests an average regional warming of  ~0.1 °C for 1910–2015 (Supplementary Fig. 12). Thus, in addition to satisfying mass conservation, any circulation changes across 27°N must also generate a heat transport divergence that, to leading order, balances the surface heat flux due to the declining subpolar sea-surface temperatures.

Knowing Florida Current transport and subpolar gyre sea-surface temperature, and requiring mass conservation at 27°N and heat conservation across the North Atlantic and Arctic north of 27°N, I estimate past changes in the deep branch of the overturning at 27^∘^N (Supplementary Note 6 for details and caveats). The estimate depends on the mean wind speed and sea-surface temperature over the warming hole as well as the vertical and horizontal temperature stratification at 27°N (Supplementary Notes 4 and 6). Making reasonable choices for these parameters, I estimate that the deep branch of the overturning slowed by 1.4 ± 1.8 Sv century^−1^ during 1909–2018 (Supplementary Fig.13a). Here  ±  is the best-estimate plus and minus twice the standard error. More generally, for long periods starting before 1950 and ending in 2018, I find that best estimates of trends in the deep return flow are northward (positive) and more than one standard error from zero, whereas for shorter periods beginning more recently, errors are larger and trends are mostly indistinguishable from zero (Supplementary Fig. 13a). This points to a probable decline in transport by the deep branch of the overturning circulation over the past 70–110 y, but reveals that any trends in the deep return flow over shorter, more recent time periods cannot be detected from these principles and knowledge of the Florida Current transport and subpolar sea-surface temperature.

I also estimate past changes in the transport of the thermocline recirculation^75^, defined as the sum of all interior upper-ocean circulations across 27°N, including the Antilles Current and Sverdrup gyre (Supplementary Note 6). Given the weakening Florida Current transport and cooling subpolar sea-surface temperature, I estimate a thermocline recirculation trend of  −0.3 ± 4.9 Sv century^−1^ for 1909–2018 (Supplementary Fig. 13b). This value is more uncertain than the change estimated in the deep branch of the overturning circulation over the past century. The difference has to do with the dependence of ocean heat transport on temperature gradients. Vertical temperature gradients are much stronger than horizontal temperature gradients at 27°N (Supplementary Fig. 14). Thus, a change in the deep branch of the overturning leads to a larger heat transport than an equal change in the thermocline recirculation, given the weakening of the Florida Current. In consequence, there is a narrow window of transport histories of the deep return flow that, together with the cooling subpolar sea-surface temperature and weakening Florida Current transport, satisfy heat and mass conservation. There is a wider window of possibility for the thermocline recirculation that allows these constraints to be met. Indeed, thermocline recirculation trends computed for other time periods are similarly uncertain (Supplementary Fig. 13b). This reveals that knowledge of past changes in the Florida Current and subpolar sea-surface temperature is relatively uninformative of the thermocline recirculation on long timescales.

## Conclusions

Lack of knowledge regarding long-term changes in major ocean currents has been a key observational uncertainty related to climate change. I applied Bayesian data analysis^42–44^ to observations from submarine cables and tide gauges to infer the changes in the Florida Current transport at 27°N during 1909–2018. I found that Florida Current transport probably declined steadily over the past century, such that transport since 1982 was likely weaker on average than it was during 1909–1981, and the weakest decadal-mean transport in the last 110 y probably took place in the past two decades. While past changes in the gyre circulation are uncertain, these results corroborate earlier hypotheses based on proxy indicators that the deep branch of the Atlantic meridional overturning circulation weakened continuously during the twentieth century^23–27^. These findings also support model simulations showing that changes in the deep return flow of the overturning circulation are nearly balanced by comparable changes in the surface western boundary current on multidecadal and longer timescales^28–32^.

Future studies should build on this foundation. Uncertainties on this Florida Current transport estimate are large. More observations could be folded into a more general probabilistic framework to better constrain the transport over the past century. My goal was to quantify how informative tide-gauge sea-level data are of past changes in the Florida Current. However, I showed that the results were consistent with exploratory analyses of GPS data, sea-level index points, and satellite altimetry. These data sets could be assimilated by specifying additional equations in the Bayesian algorithm (e.g., following ref. ^76^) to test whether Florida Current transports are better constrained and uncertainties are reduced. There are also short records from the 1960s and 1970s of transport upstream in Florida Straits (e.g., at 26°N between Miami and Bimini^19–21^) that could be leveraged, provided that the uncertain flow through Northwest Providence Channel is taken into account. With a more tightly constrained estimate, it could be possible to identify the mechanisms responsible for past changes in Florida Current transport and the deep branch of the overturning circulation, and to determine whether the interior gyre strengthened or weakened.

Future studies should try to infer Florida Current transports earlier in time. Results here show that coastal sea-level data place valuable constraints on past changes in transport. Yet, the reconstruction was limited by the duration of the available tide-gauge records, which only go back about a century or so in this region^41^. Archival tidal data going back to the mid 1800s have been identified for several locations along the southeastern USA^77^, which (if recovered and digitized) could allow for a longer reconstruction. New sea-level reconstructions derived from salt-marsh sediment along the Florida coast^78–80^, with roughly decadal temporal resolution, might make it possible to reconstruct longer-term changes in Florida Current transport over the Common Era. Pseudoproxy experiments^81^ will be informative to determine whether such a reconstruction is viable. If such a long-term reconstruction is possible, it would allow for a test of the hypothesis^13^ based on oxygen isotope records from Dry Tortugas and Grand Bahama Bank that Florida Current transport strengthened by ~10% from the mid 1700s to the mid 1900s, at the same time that other proxy indicators suggest the deep branch of the overturning circulation was stable or in decline^23–27^.

## Methods

### Observational data used in the Bayesian model

I use annual relative sea level from 46 tide gauges along the southeastern USA (19 records), Caribbean Islands (20 records), southeastern Central America (5 records), and northern South America (2 records) during 1909–2018 (Fig. 1a; Supplementary Figs. 1, 2 and Supplementary Table 3). Data were downloaded from the Permanent Service for Mean Sea Level (PSMSL) Revised Local Reference (RLR) database^41^ on 4 February 2019. The study period is the longest interval such that, for each year, data is available from at least one southeastern USA tide gauge and at least one gauge in the Caribbean Islands, southeastern Central America, or northern South America. Over the study period, each tide gauge returns on average  ~30 y of data, but some have as few as  ~10 y of data, whereas others have as many as  ~100 y. Fewer data are available earlier in time. The time series together constitute 1390 y of data over the study period (27% completeness).

I also use Florida Current transport from submarine telephone cables at 27^∘^N between West Palm Beach and Grand Bahama (Figs. 1b and 2)^1–4^. Using electromagnetic theory, one can estimate changes in the flow from voltages induced across the cable due to the transport of charged particles by the variable current^1^. The original cable spanned from Jupiter Inlet to Settlement Point, giving measurements from 18 March 1982 to 22 October 1998. Observations resumed on 19 June 2000 based on a cable running from West Palm Beach to Eight Mile Rock. Cable-based transport estimates are calibrated against independent observations from dropsonde and lowered acoustic doppler current profiler made as part of regular (roughly bi-monthly) cruises across Florida Straits^3,4^. Data are provided by the National Oceanic and Atmospheric Administration (NOAA) at 1-day intervals, but the data have an effective sampling rate of 3 days, due to low-pass filtering applied to the original observations. I use annual averages of the daily data (Fig. 2). To estimate standard errors on the annual averages, I divide the standard error on the daily data (~1.7 Sv^4^) by the square root of the effective degrees of freedom, which is the number of daily transport measurements in a year divided by the integral timescale of Florida Current transport (~10 days^3^). This gives standard errors of 0.30–0.35 Sv, depending on data availability in any given year, consistent with values computed by Garcia and Meinen^4^. Note that Bayesian model solutions are not overly sensitive to the standard errors placed on the cable data, and similar results are found if the errors are doubled (see below).

### Bayesian framework

I apply a hierarchical dynamical spatiotemporal model^42–44^ to the submarine-cable data and tide-gauge records to infer annual changes in Florida Current transport and coastal sea level. The model comprises three levels: a process level describing how the physical quantities of interest relate to one another, and vary in space and time; a data level specifying how the imperfect available data correspond to the quantities of interest; and a parameter level placing prior constraints on the uncertain parameters in the process and data levels. My model builds on the Bayesian algorithm of Piecuch et al. ^76^, who studied the origin of spatial variation in sea-level trends on the east coast of the USA during 1900–2017. Here I develop new equations to consider an expanded geographic region, incorporate the submarine-cable data, and represent the relationship between Florida Current transport and the difference in coastal sea level across the Florida Straits. See below for residual analyses and synthetic data experiments that establish the appropriateness of the model given the data, and exemplify its ability to accurately estimate the quantities of interest given the available incomplete, noisy, biased data.

### Coastal sea level

Coastal relative sea level is a process with spatiotemporal covariance^60,82^. As in Piecuch et al. ^76^, I model sea level, ${{\boldsymbol{\eta }}}_{k}={\left[{\eta }_{1,k},\ldots ,{\eta }_{N,k}\right]}^{\top }$, at steps $k\in \left\{1,\ldots ,K\right\}$ and locations $n\in \left\{1,\ldots ,N\right\}$, where *K* is the total number of time steps and *N* the total number of target locations, as the sum of a spatially correlated autoregressive process of order 1 and a large-scale spatial field of linear temporal trends,

1 $${{\boldsymbol{\eta }}}_{k}-{\boldsymbol{b}}{t}_{k}=r\left({{\boldsymbol{\eta }}}_{k-1}-{\boldsymbol{b}}{t}_{k-1}\right)+{{\boldsymbol{e}}}_{k}.$$

In Eq. (1), *t*_*k*_ is the time at step *k*, *r* is the lag-1 autocorrelation coefficient, ***b*** is the spatial vector of temporal trends, and ***e***_*k*_ is an innovation sequence, which can be interpreted as the forcing function driving changes in the autoregressive process (e.g., as in a simple stochastic climate model^83^). Supplementary Table 4 describes all of the model parameters. I set $\mathop{\sum }\nolimits_{k = 1}^{K}{t}_{k}=0$ to represent ***η***_*k*_ as anomalies from a time mean. The trend vector ***b*** is modeled as a random normal field with spatial structure, ${\boldsymbol{b}} \sim {\mathcal{N}}\left(\mu {{\bf{1}}}_{N},\Pi \right)$, such that *μ* is the spatial mean, **1**_*X*_ is a *X* × 1 column vector of ones, where *X* is any positive integer, and,

2 $${\Pi }_{ij}={\pi }^{2}\exp \big(-\lambda \big|{{\bf{s}}}_{i}-{{\bf{s}}}_{j}\big|\big).$$

Here *π*^2^ is the partial sill, *λ* is the inverse range, and $|{{\bf{s}}}_{i}-{{\bf{s}}}_{j}|$ is distance between target sites **s**_*i*_ and **s**_*j*_. The symbol  ~ means is distributed as and ${\mathcal{N}}\left({\boldsymbol{p}},{\mathtt{q}}\right)$ is the multivariate normal distribution with mean vector ***p*** and covariance matrix q.

I model ***e***_*k*_ as a temporally independent, identically distributed (iid), but spatially correlated vector with zero mean, ${{\boldsymbol{e}}}_{k} \sim {\mathcal{N}}\left({{\bf{0}}}_{N},{\mathbf{\Sigma}}\right)$, where **0**_*X*_ is a *X* × 1 column vector of zeros, and,

3 $${{\mathbf{\Sigma}}}_{ij}=\left({{\mathtt{c}}}_{ij}\right){\sigma }^{2}\exp \left(-\phi \left|{{\bf{s}}}_{i}-{{\bf{s}}}_{j}\right|\right).$$

Here *σ*^2^ is the partial sill and *ϕ* is the inverse range. Matrix element c_*ij*_ = 1 if locations **s**_*i*_ and **s**_*j*_ are either both on the southeastern USA or both along the Caribbean, Central America, or South America. Otherwise, c_*ij*_ = 0. That is, sea level covaries within, but not between, these regions. This spatial covariance structure is motivated by previous analyses of tide-gauge records and satellite-altimetry data. Thompson and Mitchum^84^ applied clustering methods to low-pass-filtered tide-gauge records during 1952–2001, finding that the Caribbean Sea (which in their analysis comprised Cuba, Puerto Rico, and Colombia) formed one cluster of coherent sea-level variation, and the southeastern USA (from Florida to North Carolina) formed another cluster. Zhao and Johns^85^ determined that Florida Current transports over 1993–2011 were positively correlated with sea-surface height over the Caribbean Sea (including the Bahamas) and along southeastern Central America, but negatively correlated with sea-surface height on the southeastern USA coast on interannual timescales; Domingues et al. ^86^ showed similar results for seasonal timescales.

### Florida Current transport

For periods ≥1 day, the momentum balance across Florida Straights is nearly geostrophic. Assuming that subsurface pressure signals are vertically coherent^49,50^, variations in Florida Current transport should therefore be correlated with changes in the sea-level difference across Florida Straits. Based on this reasoning, I assume that the relationship between annual Florida Current transport, ${\boldsymbol{T}}={\left[{T}_{1},\ldots ,{T}_{K}\right]}^{\top }$, and coastal sea level, ${\boldsymbol{\eta }}=\left[{{\boldsymbol{\eta }}}_{1},\ldots ,{{\boldsymbol{\eta }}}_{K}\right]$, at times ${\boldsymbol{t}}={\left[{t}_{1},\ldots ,{t}_{K}\right]}^{\top }$ can be written as,

4 $${\boldsymbol{T}}=\overline{T}{{\bf{1}}}_{K}+\rho {{\boldsymbol{\eta }}}^{\top }{\boldsymbol{\Delta }}+\alpha {\boldsymbol{t}}+{\boldsymbol{w}}.$$

Here $\overline{T}$ is the time-mean transport and *ρ* is a scalar coefficient representing the change in transport per unit change in sea-level difference across Florida Straits. I assume that *ρ* is constant, and does not vary with time period or frequency band. While it might appear simplistic, this assumption is justified based on analysis of an ocean general circulation model (see below; Supplementary Figs. 6 and 7). The *N* × 1 vector **Δ** is a differencing operator, such that Δ_*i*_ = 1 if site *i* is Settlement Point (the tide gauge nearest to the eastern end of the submarine cable in the Bahamas), Δ_*i*_ = −1 if site *i* is West Palm Beach (the closest tide gauge to the western end of the cable in southeastern Florida), and zero otherwise. Hence, *ρ****η***^⊤^**Δ** is the sea-level difference across Florida Straits converted into units of a transport.

The remaining terms in Eq. (4) account for influences on sea level that are unrelated to the large-scale geostrophic flow. I include *ρ****η***^⊤^**Δ** in Eq. (4) based on geostrophic considerations, but this term potentially includes both dynamic and static processes. The scalar *α* is included in Eq. (4) to correct or offset any static trends in sea level unrelated to ocean circulation that should be subtracted for estimating Florida Current transport (e.g., glacial isostatic adjustment). That is, *α****t*** is designed to remove static trends from *ρ****η***^⊤^**Δ**. Thus, with reference to Eqs. (1) and (4), ***b***^⊤^**Δ** is the total difference in sea-level trends across Florida Straits, whereas *−α*/*ρ* is the static component of the sea-level trend difference. So, the dynamic component of the trend in sea-level difference across Florida Straits (total minus static) is ***b***^⊤^**Δ** + *α*/*ρ*. As discussed in the main text, posterior Bayesian model solutions for the dynamic component are consistent with trends in dynamic sea-surface height differences across Florida Straits observed by satellite altimetry, and solutions for the static component agree with rates of static sea-level change due to differential vertical land motion between the Bahamas and Florida observed by GPS, and differences in long-term geological processes as inferred from proxy sea-level index points (Supplementary Fig. 5). I also include ${\boldsymbol{w}}={\left[{w}_{1},\ldots ,{w}_{K}\right]}^{\top }$, modeled as iid uncorrelated white noise, ${w}_{k} \sim {\mathcal{N}}\left(0,{\omega }^{2}\right)$, with variance *ω*^2^, to parameterize the response to local atmospheric or terrestrial forcing, such as variable river runoff, air pressure, or wind stress across Florida Straits.

### Tide-gauge records

Following Piecuch et al. ^76^, I represent annual data from tide gauges, ${{\boldsymbol{z}}}_{k}={[{z}_{1,k},\ldots ,{z}_{{M}_{k},k}]}^{\top }$, where *M*_*k*_ ≤ *N* is the number of tide gauges with measurements at time step *k*, as corrupted (incomplete, noisy, biased) versions of the underlying ***η***_*k*_ process,

5 $${{\boldsymbol{z}}}_{k}={{\mathtt{H}}}_{k}{{\boldsymbol{\eta }}}_{k}+{{\boldsymbol{d}}}_{k}+{{\mathtt{F}}}_{k}\left({\boldsymbol{a}}{t}_{k}+{\boldsymbol{\ell }}\right).$$

Here ***d***_*k*_ is a random error sequence, which is modeled as a spatially and temporally uncorrelated normal field, ${{\boldsymbol{d}}}_{k} \sim {\mathcal{N}}\left({{\bf{0}}}_{{M}_{k}},{\delta }^{2}{{\mathtt{I}}}_{{M}_{k}}\right)$, with variance *δ*^2^. A vector of location-specific bias offsets ***ℓ*** are imposed and represented as a spatially uncorrelated Gaussian field, ${\boldsymbol{\ell }} \sim {\mathcal{N}}\left(\nu {{\bf{1}}}_{M},{\tau }^{2}{{\mathtt{I}}}_{M}\right)$, with mean *ν*, variance *τ*^2^. Here *M* is the total number of tide gauges that make a measurement at any point in time, such that *N* ≥ *M* ≥ *M*_*k*_ ∀ *k*. Purely local error trends in the data ***a*** are also modeled as a random normal field without spatial correlation, ${\boldsymbol{a}} \sim {\mathcal{N}}\left({{\bf{0}}}_{M},{\gamma }^{2}{{\mathtt{I}}}_{M}\right)$, with variance *γ*^2^. Finally, H_*k*_ and F_*k*_ are selection matrices, filled with zeros and ones, which pick out ***η***_*k*_, ***a***, and ***ℓ*** values where observations are made at time *t*_*k*_.

### Submarine-cable measurements

I assume that *L* annual data values from the submarine cable, ${\boldsymbol{x}}={\left[{x}_{1},\ldots ,{x}_{L}\right]}^{\top }$, are available and represent imperfect (incomplete and noisy) versions of the underlying ***T*** process,

6 $${\boldsymbol{x}}={\mathtt{G}}{\boldsymbol{T}}+{\boldsymbol{u}}.$$

Here G is a *L* × *K* selection matrix that picks out years when cable data are available, and ${\boldsymbol{u}}={\left[{u}_{1},\ldots ,{u}_{L}\right]}^{\top }$ is a zero-mean random data error sequence, where ${u}_{l} \sim {\mathcal{N}}\left(0,{\xi }_{l}^{2}\right)$ and the ${\xi }_{l}^{2}$ are set equal to the corresponding submarine-cable data standard error variances mentioned above and computed based on the availability of data in any given year.

### Parameter level

To close the model, I place priors on the parameters. As in Piecuch et al.^76^, I use proper, mostly conjugate forms. Priors and hyperparameters are given in Supplementary Table 5. These choices follow the basic philosophy in Piecuch et al. ^76^. I use diffuse, mostly uninformative priors, choosing hyperparameters largely to initialize the Gibbs sampler and Metropolis-Hastings algorithm (see below) in an appropriate neighborhood of parameter space. For example, all variance parameters (e.g., *π*^2^, *σ*^2^, *δ*^2^) are given inverse-gamma priors, with shape *ξ* and scale *χ*. In all cases, I select *ξ* = 0.5 and base *χ* on the variance of the data. As explained by Tingley and Huybers^87^, this choice corresponds to 1 prior observation with a variance of 2*χ*, which is a weak constraint that has little influence on the posterior.

To quantify the relative importances of the priors and the data, after I compute the posterior solutions (see below), I compare the widths of the 95% credible intervals from the posterior and prior probability distribution functions for each parameter (Supplementary Table 6). If the prior and posterior credible intervals have similar widths, it means that the posterior solutions are largely determined by the prior assumptions. If posterior credible intervals are much narrower than the prior credible intervals, it means that the posterior solutions are mostly constrained by the observations. Almost universally, the 95% posterior credible intervals are much narrower than the 95% prior credible intervals (Supplementary Table 6). This implies that posterior inference is drawn predominantly from the information content of the observations, and not overly influenced by prior beliefs encoded into the model.

### Drawing samples from the posterior distribution

Given the model equations, I use Bayes’ rule, and assume that the posterior probability distribution function takes the form,

7 $$\begin{array}{rcl}p\left({\boldsymbol{\eta }},{\boldsymbol{T}},\Theta | {\mathtt{Z}},{\boldsymbol{x}}\right)&\propto &p\left({\mathtt{Z}},{\boldsymbol{x}}| {\boldsymbol{\eta }},{\boldsymbol{T}},\Theta \right)\times p\left({\boldsymbol{\eta }},{\boldsymbol{T}}| \Theta \right)\times p\left(\Theta \right)\hfill\\ &=&p\left({{\boldsymbol{\eta }}}_{0}\right)\times p\left(\overline{T}\right)\times p\left(r\right)\times p\left({\sigma }^{2}\right)\times p\left(\phi \right)\times p\left(\mu \right)\times p\left({\pi }^{2}\right)\hfill\\ &\times &p\left(\lambda \right)\times p\left({\delta }^{2}\right)\times p\left(\nu \right)\times p\left({\tau }^{2}\right)\times p\left({\gamma }^{2}\right)\times p\left(\rho \right)\times p\left(\alpha \right)\hfill\\ &\times &p\left({\omega }^{2}\right)\times p\left({\boldsymbol{b}}| \mu ,{\pi }^{2},\lambda \right)\times p\left({\boldsymbol{\ell }}| \nu ,{\tau }^{2}\right)\times p\left({\boldsymbol{a}}| {\gamma }^{2}\right)\times p\left({\boldsymbol{x}}| {\boldsymbol{T}}\right)\hfill\\ &\times &p\left({\boldsymbol{T}}| {\boldsymbol{\eta }},\rho ,\alpha ,{\omega }^{2},\overline{T}\right)\times \mathop{\prod }\nolimits_{k = 1}^{K}\left[p\left({{\boldsymbol{z}}}_{k}| {{\boldsymbol{\eta }}}_{k},{\boldsymbol{a}},{\boldsymbol{\ell }},{\delta }^{2}\right)\times p\left({{\boldsymbol{\eta }}}_{k}| {{\boldsymbol{\eta }}}_{k-1},{\boldsymbol{b}},r,{\sigma }^{2},\phi \right)\right]\hfill\end{array}$$

In Eq. (7), Z is the structure of all tide-gauge data points, *p* is used to represent probability distribution function, ∣ is conditionality,  ∝  is proportionality, and $\Theta \mathop{=}\limits^{.}\left\{r,{\sigma }^{2},\phi ,\ldots \right\}$ is used to represent the set of all model parameters. I assume that the observations are conditionally independent, provided the process and parameters.

Draws from the posterior probability distribution function are made as in Piecuch et al.^76^. I use Markov chain Monte Carlo (MCMC) methods, evaluating the full conditional distributions for process and parameter values using a Gibbs sampler, but using Metropolis-Hastings steps for the inverse range parameters, which have non-standard full conditionals. I run 200,000 MCMC iterations, setting initial process values to zero, and drawing initial parameter values randomly from the respective prior distribution. To remove initialization transients, I discard the first 100,000 iterations as burn in. Then I keep only 1 out of every 100 of the remaining 100,000 iterations to reduce serial correlation effects between draws. Results shown here are based on a 3000-element chain produced by performing the above procedure three times and stitching together the resulting 1000-member chains. Solutions for scalar parameters are summarized in Supplementary Table 6. To evaluate convergence of the solution, I compute the convergence monitor $\hat{R}$ of Gelman and Rubin^88^, which compares the variance within and between the three different 1,000-member solutions. In all cases, $\hat{R} \sim 1.00$ (Supplementary Table 6), indicating that the solutions have converged.

### Local and global uncertainty measures

The fully probabilistic nature of the posterior solutions allows both pointwise and pathwise uncertainty measures^89^ to be calculated. Pointwise statistics measure probabilities locally. The light blue shading in Fig. 2 represents the 95% pointwise posterior credible intervals computed from the transport solutions at each year of the reconstruction. The interpretation is that, for each year, there is a 95% chance that the true transport value falls within this blue shading.

Pathwise statistics measure probabilities more globally. The dashed blue lines in Fig. 2 represent the 95% pathwise posterior credible intervals calculated from the transport estimates across all years of the reconstruction. These values are computed by widening the 95% pointwise posterior credible intervals until 95% of modeled transport time series are captured in their entirety. That is, there is a 95% chance that the full time series of transport does not ever stray outside the bounds of these pathwise credible intervals.

Other examples of pathwise statistics include values quoted in the text for the minimum and maximum decadal-average transports and the corresponding histograms of their timing shown Fig. 4. For each of the 3000 ensemble members comprising the posterior solution, I smooth the transport time series using an 11-point boxcar window, and then identify the minimum and maximum transport values along with the times at which they occurred. These values vary from one ensemble member to the next, and so performing this procedure for each ensemble member allows me to populate histograms for the transport extrema and their occurrence times.

### Hypothesis testing

In addition to generating posterior solutions for transport and sea level, the Bayesian model provides data-constrained estimates of the various model parameters (e.g., Supplementary Table 6). This allows for rigorous hypothesis testing through simulation experiments. For example, in Fig. 3, I show the change in decadal-average Florida Current transport between all possible pairs of non-overlapping decades, and indicate the probability that such changes would have occurred from stationary red-noise process with the same autocorrelation and variance characteristics. As a specific instance, I state that decadally averaged transport declined by  −1.4 ± 1.6 Sv from 1960–1970 to the present, and that this decline is very likely (probability *P* = 0.90) more than would be expected from stationary red noise. This conclusion is determined as follows. First, I use the posterior transport solutions to compute a histogram of transport averaged over 2008–2018 minus the transport averaged over 1960–1970. Next, I use the posterior solutions for the scalar model parameters as the basis for the simulation of a parallel set of 3000 synthetic transport time series following Eqs. (1) and (4), but with the trends (***b*** and *α*) set to zero. Then, I populate histograms of the difference between decadally averaged synthetic transport between 1960–1970 and 2008–2018. Finally, I compute what fraction of the original posterior transport solutions shows more of a decline than is shown by the stationary synthetic transport process, which, in this example case, is 0.90.

### Residual analysis

Various residual terms appear in the Bayesian model equations (see above). When building the model, I made certain assumptions regarding the spatial and temporal structures of these residuals. To test whether these assumptions are appropriate given the data, I perform a residual analysis, using the posterior solutions and model equations to solve for the sea-level innovations ***e***_*k*_, tide-gauge errors ***d***_*k*_, transport innovations *w*_*k*_, cable-data errors ***u***, tide-gauge error trends ***a***, and tide-gauge data bias ***ℓ***.

I assumed that ***e***_*k*_, ***d***_*k*_, *w*_*k*_, and ***u*** behave as iid temporal white noise. If this assumption is reasonable, then posterior solutions should look random in time. However, if systematic temporal structure is observed, it means that this assumption is inappropriate, and that the model is misspecified given the data. Time series of posterior ***e***_*k*_ and ***d***_*k*_ solutions are shown in Supplementary Fig. 5a, b for an arbitrary (randomly chosen) target location, while model solutions for *w*_*k*_ and ***u*** are shown in Supplementary Fig. 15c, d. The time series look random in time, and there are no obvious signs of autocorrelation. The amplitudes of ***e***_*k*_, ***d***_*k*_, and *w*_*k*_ variations are consistent with posterior solutions for the respective variance or partial sill parameters *σ*^2^, *δ*^2^, and *ω*^2^ (Supplementary Table 6), and the magnitude of fluctuations in ***u*** is in keeping with the prior error variances placed on the submarine-cable data.

To be more thorough, I compute sample autocorrelation coefficients directly from the posterior solutions for ***e***_*k*_, ***d***_*k*_, *w*_*k*_, and ***u*** across all space and time points. I compare those values to the autocorrelation coefficients expected theoretically for temporal white noise, given the same number of time steps. Supplementary Figure 16 compares the empirical and theoretical autocorrelation coefficients for time lags between 1 and 20 y. Values calculated empirically from the posterior solutions are consistent with the theoretically expected values. More quantitatively, 96%, 95%, 93%, and 95% of empirical autocorrelation coefficients computed respectively from ***e***_*k*_, ***d***_*k*_, *w*_*k*_, and ***u*** are captured by the theoretical 95% confidence intervals.

In addition to being random in time, ***e***_*k*_ and ***d***_*k*_ are supposed to have spatially invariant amplitudes. In Supplementary Fig. 17, I map median estimates of standard deviations computed empirically from the posterior model solutions of ***e***_*k*_ and ***d***_*k*_ at each tide-gauge location. While there is some higher-order spatial variation, these values are to lowest order fairly uniform and constant in space, and very similar to the posterior estimates of the partial sill *σ*^2^ and variance parameter *δ*^2^ (Supplementary Table 6).

Motivated by past studies^84–86^, I assume that ***e***_*k*_ is spatially structured, such that there is covariance between sites along the Caribbean, Central America, and South America, and between sites on the southeastern USA, but no covariance between these two broad regions. These assumptions are reflected in the block structure of the theoretical covariance matrix **Σ** shown in Supplementary Fig. 18b computed from the posterior median solution for the partial sill *σ*^2^ (Supplementary Table 6). This theoretical covariance matrix is very similar to the covariance matrix determined empirically by comparing all pairs of posterior solutions for ***e***_*k*_ (Supplementary Fig. 18a). Indeed, the Pearson correlation coefficient between the two matrices in Supplementary Fig. 18 is 0.91, and the theoretical covariance matrix explains 82% of the variance in the empirical covariance matrix.

In Supplementary Fig. 19a, I show a map of median values of Pearson correlation coefficients computed between posterior solutions for the Florida Current transport and sea level at every coastal location after removing linear trends from the time series. Correlation coefficients are positive over the Caribbean, Central America, and South America, and negative along the southeastern USA. The magnitude of the correlation decreases with increasing distance of the sea-level location from the Florida Straits. This behavior of the empirically determined correlation coefficients is broadly consistent with the correlation pattern expected theoretically (Supplementary Fig. 19b), given Eqs. (3) and (4) and posterior solutions for the model parameters (Supplementary Table 6).

Finally, I consider residual spatial fields of the tide-gauge data biases ***ℓ*** − *ν***1** and error trends ***a***. According to data level Eq. (5) for the tide gauges, these two vectors should have zero mean, no spatial correlation, and spatial variances of *τ*^2^ and *γ*^2^, respectively. Supplementary Fig. 20 facilitates an assessment of these assumptions, showing both posterior solutions for ***ℓ*** − *ν***1** and ***a*** alongside the solutions expected for a zero-mean random process given the posterior solutions for *τ*^2^ and *γ*^2^ (Supplementary Table 6). Consistent with model assumptions, these vector fields look fairly random, scattered about zero. The spatial spread in ***ℓ*** − *ν***1** and ***a*** appears consistent with the posterior *τ*^2^ and *γ*^2^ solutions. Indeed, 95% of the posterior ***ℓ*** − *ν***1** solutions are captured by the 95% credible intervals predicted for a zero mean, spatially uncorrelated Gaussian process with variance *τ*^2^, and similarly 95% of posterior solutions for ***a*** fall within the 95% credible interval produced by simulating a zero-mean random normal field with variance *γ*^2^ (Supplementary Fig. 20).

In conclusion, the design of my Bayesian algorithm is supported by residual analysis, which demonstrates that the model structure is appropriate and warranted given the available data.

### Sensitivity of model solutions to input data

Posterior solutions for Florida Current transports in the main text are based on the assimilation of submarine-cable data over 1982–2018 with standard errors of 0.30–0.35 Sv (see above). To quantify how robust or sensitive the solutions are to the duration of the data and the selected standard errors, I perform two additional data assimilation experiments. In the first experiment, I double the standard errors on the cable data given to the Bayesian algorithm during 1982–2018. I refer to this first experiment as the double-error experiment. For clarity, in this section, I call the Bayesian model solution from the main text the baseline experiment. In the second sensitivity experiment, I maintain the original standard errors, and I give the Bayesian algorithm all of the cable measurements for the period 2000–2018, but I withhold all data during 1982–1998. Due to an outage in the observing system, no data are available for 1999. I call this second experiment the half-data experiment.

Salient features of the sensitivity experiments are summarized alongside the baseline experiment in Supplementary Fig. 21. Baseline and double-error solutions are, in many respects, very similar. For example, Florida Current transport during 1909–1981, transport trend over 1909–2018, and regression coefficient between transport and sea-level difference across the Florida Straits from these two experiments are nearly the same (cf. blue and orange in Supplementary Fig. 21). One difference is that widths of the posterior 95% credible intervals on the transport during 1982–2018 (i.e., the period when transport observations are available) are about twice as large in the double-error experiment compared to the baseline experiment (Supplementary Fig. 21a). This is consistent with the larger standard errors placed on the data in the former experiment. In sum, I conclude that model solutions are generally quantitatively insensitive to reasonable alternative specifications of the standard error on the cable transport measurements.

Solutions from the half-data experiment (yellow in Supplementary Fig. 21) show similarities to the other two solutions, but can show larger uncertainty. This is unsurprising, since the half-data experiment has fewer data constraints. For example, whereas the posterior 95% credible intervals on the 110-y transport trend are  −1.7 ± 3.7 and  −1.6 ± 3.9 Sv century^−1^ in the baseline and double-error experiments, in the half-data experiment it is  −2.3 ± 6.9 Sv century^−1^. The fact that uncertainties from the double-error experiment are smaller than from the half-data experiment suggests that having more data with larger errors is more informative for constraining the transport history than having fewer data that have smaller errors. Importantly, although the trend from the half-data experiment is more uncertain in an absolute sense, the sign of the trend is similarly determined in all three experiments. I find that 82%, 80%, and 77% of trend solutions in the baseline, double-error, and half-data experiments are negative, respectively (Supplementary Fig. 21b). That is, all three experiments suggest that Florida Current transport probably declined over the past century. Thus, I reason that the main findings in this study are qualitatively robust to reasonable alternative choices for the duration of the transport data assimilated into the Bayesian algorithm.

### Synthetic data experiments

In the half-data experiment,  ~90% of the withheld Florida Current transport values during 1982–1998 fall within the pointwise posterior 95% credible intervals on the transport. This suggests that the uncertainties estimated by the Bayesian algorithm are reasonable. To more thoroughly evaluate the meaningfulness of the posterior solutions from the Bayesian algorithm, I perform a number of synthetic data experiments. In these experiments, I take a set of known processes and corrupt them to look like the observations, and then apply the model to the corrupted process values. By comparing the posterior solutions to the known but withheld values, I quantify the accuracy and precision of the error bars estimated by the model (e.g., are  ~95% of the true values actually captured by the posterior 95% credible intervals?).

### Perfect model synthetic data experiment

I run a perfect model experiment. I choose, from the ensemble of posterior model solutions presented in the main text, the array of scalar parameter solutions $\left(\overline{T},r,{\sigma }^{2},\ldots \right)$ from the ensemble member that minimizes the Mahalanobis distance to the mean parameter array. Using these scalar parameter values, I simulate synthetic versions of the sea-level and transport processes based on the process-level equations. Using the data-level equations, I generate synthetic tide gauge and submarine-cable data by adding noise, bias, and gaps to the simulated processes, as in the real world, and I apply the Bayesian model to these synthetic data.

Results are summarized in Supplementary Table 7 and Supplementary Fig. 22. For 13 out of the 14 scalar parameters, or  ~93%, the true value is captured by the corresponding 95% posterior credible interval from the model (Supplementary Table 7). Considering vector fields, I find that 100%, 98%, and 100% of the true values for regional sea-level trends ***b***, tide-gauge biases ***ℓ***, and tide-gauge error trends ***a***, respectively, fall within the corresponding pointwise posterior 95% credible intervals (not shown). In terms of the processes, 98% of the true sea-level values and 99% of true transport values fall within the estimated pointwise 95% credible intervals, and the true transport time series is entirely encompassed by the pathwise 95% posterior credible intervals (e.g., Supplementary Fig. 22). Together, these results show that the model performs well, and that the posterior credible intervals are meaningful, albeit slightly conservative, roughly capturing the correct fraction of true process and parameter values.

### More realistic synthetic data experiment

The first synthetic data experiment is informative, showing that the processes and parameters are identifiable given incomplete, noisy, biased data. It is also potentially idealistic, since the model is perfectly specified. The equations governing the spatiotemporal evolution of the processes, and the relationship between the observations and the processes were known perfectly, and the task was to infer the uncertain values of the processes and parameters appearing in those equations. While residual analysis suggests that they are appropriate given the data, the model equations probably represent a simplification of the complex, myriad oceanographic, and geophysical processes contributing to changes in sea level and transport, and their correspondence to observations in the real world. While some degree of model misspecification is inevitable, the salient question is whether the model is robust to misspecification and still provides meaningful posterior estimates.

So, I perform a second synthetic data experiment. Rather than use the process equations to simulate sea level and transport, I bring together output from more complex physical models. I begin with ocean dynamics. I take 110 y of monthly Florida Current transport near 27°N, and sea level from each of the model grid cells nearest to the 46 tide gauges from version 2.2.4 of the Simple Ocean Data Assimilation (SODA) product^52^. This version of SODA represents a solution to an ocean general circulation model forced at the surface by an atmospheric reanalysis over the period 1871–2010 (I use the past 110 y of output covering from 1901 to 2010). The model has moderate spatial resolution, with 40 vertical levels and a native 0.25° × 0.40° horizontal grid in longitude and latitude. A version of the solution, which was interpolated onto a regular 0. 5° × 0.5° horizontal grid, was downloaded from the Asia-Pacific Data-Research Center (APDRC) of the University of Hawai’i School of Ocean and Earth Science and Technology. After downloading, I removed the monthly time series of global-mean sea level and computed annual means from the resulting monthly sea-level values.

The SODA solution represents a tradeoff between spatial resolution and temporal coverage. Coupled climate models are available that cover comparable or longer time periods^90^, but most publicly available solutions have coarser horizontal resolution (nominally  ~1° in longitude and latitude), and may not faithfully represent the Florida Current and coastal sea level. While much higher-resolution ocean models are available^91^ that more accurately portray the complexity of Florida Current transport and coastal sea level, these model runs are typically short, and do not span the centennial timescales of primary interest here. Thus, while it has its deficiencies (see below), SODA is perhaps one of the best-suited ocean models for my purposes. For example, Chepurin et al.^92^ show that version 2.2.4 of SODA simulates interannual-to-decadal variations in coastal sea level along the eastern USA and parts of the Caribbean, Central America, and South America reasonably well over 1950–2011.

I superimpose static sea-level effects on the dynamic sea-level fields from SODA. I add a yearly time series of global-mean sea level due to ocean warming and thermal expansion over 1901–2010 from the Version 4 of the Community Climate System Model^93^ (downloaded from the Woods Hole Oceanographic Institution’s Community Storage Server). I also include, at each tide-gauge location, an estimate of the trend in relative sea level due to the combined effects of ongoing glacial isostatic adjustment from Peltier et al.^94^ (downloaded from the PSMSL) along with twentieth-century melting of mountain glaciers and ice sheets due to Hamlington et al.^95^ (courtesy of S. Adhikari, Jet Propulsion Laboratory). Finally, I add time series of a temporally random but spatially correlated process with zero mean and temporal variance of  ~(1 cm)^2^ to simulate sea-level changes due to the inverted-barometer effect linked with the North Atlantic Oscillation^96^.

I apply the data-level equations to the transport and sea-level values, incorporating noise and bias, and imparting data gaps so that the synthetic tide gauge and submarine-cable data are only available when and where the true observations are available. The synthetic data sets are subsequently fed into the Bayesian algorithm. The results of this second synthetic data experiment are summarized in Supplementary Table 8 and Supplementary Fig. 23. In this case, only four scalar parameters (those appearing in the data-level equations) are known perfectly. For three out of these four parameters, or 75%, the true value is captured by the 95% posterior credible intervals from the model (Supplementary Table 8). For one parameter, *δ*^2^, the tide-gauge data error variance, the Bayesian model slightly underestimates the true value. Considering the process time series, I find that 81% of the true transport values and 95% of the true sea-level values are captured by the pointwise 95% posterior credible intervals produced by the Bayesian model, and that, as in the previous experiment, the full time series of the true transport is totally captured by the pathwise 95% posterior credible interval (Supplementary Fig. 23).

It is worth noting that the posterior solution for *α*, the apparent trend in the transport process Eq. (4), suggests that sea level at Settlement Point on Grand Bahama must have risen 0.2 ± 1.6 mm y^−1^ faster than at West Palm Beach near West Palm Beach due to processes unrelated to ocean dynamics. Although uncertain, this is consistent with the trend difference of  ~0.1 mm y^−1^ I imposed between these two sites based on model estimates of GIA and contemporary ice melt^94,95^, demonstrating that the model succeeds in separating static and dynamic sea-level trends.

Recall that my Bayesian model assumes that the transfer coefficient *ρ* between sea level and transport is a fixed constant. To test this assumption, I consider in more detail time series of Florida Current transport and sea-level difference across Florida Straits from SODA. Transport and sea-level difference are highly correlated with one another (Pearson correlation coefficient of  ~0.9), and a linear regression suggests that transport increases by  ~0.9 Sv for every 1-cm increase in sea level difference (Supplementary Fig. 7), consistent with a visual inspection of the two time series (Supplementary Fig. 6a). To study the correspondence as a function of frequency band, I apply admittance and coherence analysis^53^ to the model output. Transport and sea-level difference are significantly coherent at all accessible periods from 2- to 32-y (Supplementary Fig. 6b), in agreement with basic expectations from geostrophy. Moreover, the transfer function (using sea-level difference as the input and transport as the output) is qualitatively insensitive to frequency band, with similar values found at interannual and multidecadal timescales (Supplementary Fig. 6c). Importantly, the Bayesian model posterior estimate for the transfer coefficient *ρ* is consistent with SODA and overlaps the values obtained from the admittance analysis (Supplementary Figs. 6 and 7). This suggests that it is reasonable to assume that there is a constant transfer coefficient between sea-level difference and transport on the timescales of this study, and also that the Bayesian model successfully infers the correct transfer-coefficient value.

Note that Florida Current transport from SODA is suspicious (Supplementary Fig. 23c). The mean transport is  ~51 Sv, growing from  ~42 Sv at the beginning of the period to  ~56 Sv at the end. This value is  ~60% larger than the average value observed by submarine cable since 1982, and  ~10 Sv larger than the largest annual transport value inferred at any time in the original Bayesian model solution discussed in the main text. The striking increase of  ~14 Sv over the 110-y run seems extreme in light of the more subtle trend estimates produced by the original Bayesian model solution (cf. Fig. 2; Supplementary Fig. 23c). Although it is imperfect, in that it does not realistically represent the true evolution of the Florida Current over the past century, SODA is nevertheless informative in the present context. For establishing the ability of the Bayesian algorithm to infer the parameters and processes from imperfect data, I do not require that SODA reproduces observed reality, but rather that it portrays a physically plausible scenario, and that the basic statistics (e.g., spatiotemporal covariance structure, relationship between state variables, etc.) are believable.

In sum, I conclude that, even in a more complex setting, my Bayesian model performs reasonably well, giving uncertainty estimates that roughly capture the correct fraction of true values.

## Supplementary information

Supplementary Information

Peer Review File

## Data Availability

The source data underlying Fig. 2 are provided as a Source Data file. The probabilistic reanalysis of annual Florida Current transport is also available from 10.5281/zenodo.3928585. The tide-gauge data and submarine-cable data that support the main findings of this study are available from the Permanent Service for Mean Sea Level (PSMSL; http://www.psmsl.org/) and the National Oceanic and Atmospheric Administration Atlantic Oceanographic and Meteorological Laboratory (NOAA AOML; https://www.aoml.noaa.gov/), respectively. Ancillary data sets, used for interpretation and not incorporated into the Bayesian model, and their availabilities are as follows: surface-drifter data of surface current speeds shown in Fig. 1a are available from NOAA AOML; Global Digital Elevation Model bathymetry shown in Figs. 1b,  4 and Supplementary Fig. 14 are available from NOAA National Geophysical Data Center (NGDC; https://www.ngdc.noaa.gov/); Cruise data from the research vessel Walton Smith shown in Fig. 5 are available from NOAA AOML; reanalysis wind-stress fields shown in Fig. 6 and Supplementary Fig. 10 are available from the Woods Hole Oceanographic Institution Community Storage Server (WHOI CCS; https://cmip5.whoi.edu); Gridded data sets of sea-surface temperature shown in Figs. 7 and 8 are available from the UK Met Office Hadley Centre (https://www.metoffice.gov.uk/hadobs/) and NOAA Earth System Research Laboratory Physical Sciences Division (ESRL PSD; https://www.esrl.noaa.gov/psd/); results from the model-data synthesis of Gebbie and Huybers^73^ shown in Supplementary Fig. 12 are available from the NOAA National Centers for Environmental Information (NOAA NCEI; https://data.nodc.noaa.gov/cgi-bin/iso?id=noaa-model-26611); climatological ocean temperatures shown in Supplementary Fig. 14 are from the World Ocean Atlas 2018 available from NOAA NCEI (https://www.nodc.noaa.gov/OC5/woa18/); time series of climate indices shown in Supplementary Fig. 4 are available from NOAA ESRL PSD; Global Positioning System data of vertical land motion rates shown in Supplementary Table 1 are available from Système d’Observation du Niveau des Eaux Littorales (SONEL; http://www.sonel.org/); Proxy relative sea-level index points shown in Supplementary Table 2 are taken from Khan et al.^56^ and Love et al.^57^; satellite-altimetric time series of sea-surface height shown in Supplementary Fig. 9 are available from Centre of Topography of the Oceans and the Hydrosphere (CTOH; http://ctoh.legos.obs-mip.fr/); model estimates of glacial isostatic adjustment rates used in the synthetic data experiments are available from PSMSL; Global-mean thermosteric sea level from the Community Climate System Model Version 4 used in the synthetic data experiments was downloaded from the WHOI CCS; Model solutions from the Simple Ocean Data Experiment (SODA) shown in Supplementary Figs. 6, 7, and 23, and used in the synthetic data experiments are available from the University of Hawaii Asia-Pacific Data-Research Center (http://apdrc.soest.hawaii.edu/). Source data are provided with this paper.

## Source data

Source Data

## Code availability

The computer code used to run the Bayesian model and produce the results in this study, written in the MATLAB software environment, is available at the corresponding author’s GitHub website (https://github.com/christopherpiecuch), along with the complete Bayesian model solution for all processes and parameters. Source data are provided with this paper.

## References

[1] Larsen JC (1992). Transport and heat flux of the Florida Current at 27^∘^ N derived from cross-stream voltages and profiling data: theory and observations. Philos. Trans. R. Soc. A.

[2] Baringer MO, Larsen JC (2001). Sixteen years of Florida Current transport at 27° N. Geophys. Res. Lett..

[3] Meinen CS, Baringer MO, Garcia RF (2010). Florida Current transport variability: an analysis of annual and longer-period signals. Deep-Sea Res.

[4] Garcia RF, Meinen CS (2014). Accuracy of Florida Current volume transport measurements at 27°N using multiple observational techniques. J. Atmos. Ocean. Technol..

[5] Lee TN, Johns WE, Zantopp RJ, Fillenbaum ER (1996). Moored observations of western boundary current variability and thermohaline ciruclation at 26.5^∘^N in the subtropical North Atlantic. J. Phys. Oceanogr..

[6] Johns WE (2008). Variability of shallow and deep western boundary currents off the Bahamas during 2004-05: results from the 26^∘^N RAPID-MOC array. J. Phys. Oceanogr..

[7] Frajka-Williams E, Johns WE, Meinen CS, Beal LM, Cunningham SA (2013). Eddy impacts on the Florida Current. Geophys. Res. Lett..

[8] Meinen CS (2019). Structure and variability of the Antilles Current at 26.5^∘^N. J. Geophys. Res. Oceans.

[9] Sverdrup HU (1947). Wind-driven currents in a baroclinic ocean; with application to the Equatorial currents of the Eastern Pacific. Proc. Natl Acad. Sci. USA.

[10] Stommel H (1948). The westward intensification of wind-driven ocean currents. EOS T. Am. Geophys. Un..

[11] Schmitz WJ, Thompson JD, Luyten JR (1992). The Sverdrup circulation for the Atlantic along 24^∘^N. J. Geophys. Res..

[12] McCarthy GD (2015). Measuring the Atlantic meridional overturning circulation at 26^∘^N. Prog. Oceanogr..

[13] Lund DC, Lynch-Stieglitz J, Curry WB (2006). Gulf Stream density structure and transport during the past millennium. Nature.

[14] Lynch-Stieglitz J (2017). The Atlantic meridional overturning circulation and abrupt climate change. Annu. Rev. Mar. Sci..

[15] Palter JB (2015). The role of the Gulf Stream in European climate. Annu. Rev. Mar. Sci..

[16] Pillsbury JE (1890). The Gulf Stream–a description of the methods employed in the investigation, and the results of the research. Rept. Supt. US Coast Geod. Surv..

[17] Hela I (1952). The fluctuations of the Florida Current. B. Mar. Sci. Gulf Carib.

[18] Wunsch C, Hansen DV, Zetler BD (1969). Fluctuations of the Florida Current inferred from sea level records. Deep-Sea Res..

[19] Niiler PP, Richardson WS (1973). Seasonal variability of the Florida Current. J. Mar. Res..

[20] Schmitz WJ, Richardson WS (1968). On the transport of the Florida Current. Deep-Sea Res..

[21] Brooks IH, Niiler PP (1977). Energetics of the Florida Current. J. Mar. Res..

[22] Richardson W, Schmitz W, Niiler P (1969). The velocity structure of the Florida Current from the Straits of Florida to Cape Fear. Deep-Sea Res..

[23] Rahmstorf S (2015). Exceptional twentieth-century slowdown in Atlantic Ocean overturning circulation. Nat. Clim. Change.

[24] Caesar L, Rahmstorf S, Robinson A, Feulner G, Saba V (2018). Observed fingerprint of a weakening Atlantic Ocean overturning circulation. Nature.

[25] Thornalley DJR (2018). Anomalously weak Labrador Sea convection and Atlantic overturning during the past 150 years. Nature.

[26] Thibodeau B (2018). Last century warming over the Canadian Atlantic shelves linked to weak Atlantic meridional overturning circulation. Geophys. Res. Lett..

[27] Moffa-Sánchez P (2019). Variability in the northern North Atlantic and Arctic Oceans across the last two millennia: a review. Paleoceanogr. Paleocl..

[28] Thomas MD, de Boer AM, Stevens DP, Johnson HL (2012). Upper ocean manifestations of a reducing meridional overturning circulation. Geophys. Res. Lett..

[29] Beadling RL, Russell JL, Stouffer RJ, Goodman PJ (2018). Evaluation of subtropical North Atlantic Ocean circulation in CMIP5 models against the observational array at 26.5^∘^N and its changes under continued warming. J. Clim..

[30] Gu S, Liu Z, Wu L (2020). Time scale dependence of the meridional coherence of the Atlantic meridional overturning circulation. J. Geophys. Res. Oceans.

[31] Moreno-Chamarro E, Ortega P, González-Rouco F, Montoya M (2017). Assessing reconstruction techniques of the Atlantic Ocean circulation during the last millennium. Clim., Dyn..

[32] Gu S (2019). Assessing the ability of zonal *δ*^18^ O contrast in benthic foraminifera to reconstruct deglacial evolution of Atlantic meridional overturning circulation. Paleoceanogr. Paleocl..

[33] Maul GA, Chew F, Bushnell M, Mayer DA (1985). Sea level variation as an indicator of Florida Current volume transport: comparisons with direct measurements. Science.

[34] Park J, Sweet W (2015). Accelerated sea level rise and Florida Current transport. Ocean Sci..

[35] Schott F, Zantopp R (1985). Florida Current: seasonal and interannual variability. Science.

[36] Iselin CO (1940). Preliminary report on long-period variations in the transport of the Gulf-Stream system. Pap. Phys. Oceanogr. Meteorol..

[37] Sturges, W. & Hong, B. G., Gulf Stream transport variability at periods of decades, *J*. *Phys. Oceanogr.* **31**, 1304–1312 (2001).

[38] Kopp RE, Hay CC, Little CM, Mitrovica JX (2015). Geographic variability of sea-level change. Curr. Clim. Change Rep..

[39] Horton BP (2018). Mapping sea-level change in time, space, and probability. Annu. Rev. Environ. Resour..

[40] Woodworth PL (2019). Forcing factors affecting sea level changes at the coast. Surv. Geophys..

[41] Holgate SJ (2013). New data systems and products at the permanent service for mean sea level. J. Coast. Res..

[42] Tingley MP, Craigmile PF, Haran M, Li B, Mannshardt E (2012). Piecing together the past: statistical insights into paleoclimatic reconstructions. Quat. Sci. Rev..

[43] Ashe EL (2019). Statistical modeling of rates and trends in Holocene relative sea level. Quat. Sci. Rev..

[44] Cressie, N. & Wikle, C. K. *Statistics for Spatio-Temporal Data* 588 (John Wiley & Sons, 2011).

[45] Sheinbaum J, Candela J, Badan A, Ochoa J (2002). Flow structure and transport in the Yucatan Channel. Geophys. Res. Lett..

[46] Jones PD, Jonsson T, Wheeler D (1997). Extension to the North Atlantic Oscillation using early instrumental pressure observations from Gibraltar and South-West Iceland. Int. J. Climatol..

[47] DiNezio PN, Gramer LJ, Johns WE, Meinen CS, Baringer MO (2009). Observed interannual variability of the Florida Current: wind forcing and the North Atlantic oscillation. J. Phys. Oceanogr..

[48] Enfield DB, Mestas-Nuñez AM, Trimble PJ (2001). The Atlantic multidecadal oscillation and its relation to rainfall and river flows in the continental U.S. Geophys. Res. Lett..

[49] Bingham RJ, Hughes CW (2009). Signature of the Atlantic meridional overturning circulation in sea level along the east coast of North America. Geophys. Res. Lett..

[50] Little CM (2019). The relationship between United States East Coast sea level and the Atlantic meridional overturning circulation: a review. J. Geophys. Res. Oceans.

[51] Leaman KD, Molinari RL, Vertes PS (1987). Structure and variability of the Florida Current at 27°N: April 1982-July 1984. J. Phys. Oceanogr..

[52] Giese BS, Ray S (2011). El Ninõ variability in simple ocean data assimilation (SODA), 1871-2008. J. Geophys. Res..

[53] Emery, R. E. & Thomson, W. J. *Data Analysis Methods in Physical Oceanography* 3rd edn, 728 (Elsevier, 2014).

[54] Gregory JM (2019). Concepts and terminology for sea level: mean, variability, and change, both local and global. Surv. Geophys..

[55] Santamaría-Gómez A (2017). Uncertainty of the 20th century sea-level rise due to vertical land motion errors. Earth Planet. Sci. Lett..

[56] Khan NS (2017). Drivers of Holocene sea-level change in the Caribbean. Quat. Sci. Rev..

[57] Love R (2016). The contribution of glacial isostatic adjustment to projections of sea-level change along the Atlantic and Gulf coasts of North America. Earthas Future.

[58] Birol F (2017). Coastal applications from nadir altimetry: example of the X-TRACK regional products. Adv. Space Res..

[59] Theiler J, Eubank S, Longtin A, Galdrikian B, Farmer JD (1992). Testing for nonlinearity in time series: the method of surrogate data. Phys. D.

[60] Bos, M. S., Williams, S. D. P., Araújo, I. B. & Bastos, L. The effect of temporal correlated noise on the sea level rate and acceleration uncertainty. *Geophys. J. Int.* **196**, 1423–1430 (2014).

[61] Compo GP (2011). The Twentieth Century Reanalysis Project. Q. J. R. Meteorol. Soc..

[62] Poli P (2016). ERA-20C: An atmospheric reanalysis of the twentieth century. J. Clim..

[63] Leetmaa A, Bunker AF (1978). Updated charts of the mean annual wind stress, convergences in the Ekman layers, and Sverdrup transports in the North Atlantic. J. Mar. Res..

[64] Böning CW, Döscher R, Isemer J-J (1991). Monthly mean wind stress and Svedrup transports in the North Atlantic: a comparison of the Hellerman-Rosenstein and Isemer-Hasse climatologies. J. Phys. Oceanogr..

[65] Johns WE, Townsend TL, Fratatoni DM, Wilson WD (2002). On the Atlantic inflow to the Caribbean Sea. Deep-Sea Res.

[66] Smeed DA (2018). The North Atlantic Ocean is in a state of reduced overturning. Geophys. Res. Lett..

[67] Drijfhout S, van Oldenborgh GJ, Cimatoribus A (2012). Is a decline of AMOC causing the warming hole above the North Atlantic in observed and modeled warming patterns?. J. Clim..

[68] Roberts CD, Garry FK, Jackson LC (2013). A multimodel study of sea surface temperature and subsurface density fingerprints of the Atlantic meridional overturning circulation. J. Clim..

[69] Rayner NA (2003). Global analyses of sea surface temperature, sea ice, and night marine air temperature since the late nineteenth century. J. Geophys. Res..

[70] Kaplan A (1998). Analyses of global sea surface temperature 1856-1991. J. Geophys. Res..

[71] Lozier MS (2010). Deconstructing the conveyor belt. Science.

[72] Buckley MW, Marshall J (2016). Observations, inferences, and mechanisms of Atlantic meridional overturning circulation variability: a review. Rev. Geophys..

[73] Gebbie G, Huybers P (2019). The Little Ice Age and 20th-century deep Pacific cooling. Science.

[74] Zanna L, Khatiwala S, Gregory JM, Ison J, Heimbach P (2019). Global reconstruction of historical ocean heat transport and transport. Proc. Natl Acad. Sci. USA.

[75] Kanzow T (2007). J. Observed flow compensation associated with the MOC at 26. 5°N in the Atlantic. Science.

[76] Piecuch CG (2018). Origin of spatial variation in US East Coast sea-level trends during 1900–2017. Nature.

[77] Talke SA, Jay DA (2013). Nineteenth century North American and Pacific tidal data: lost or just forgotten?. J. Coast. Res..

[78] Kemp AC (2014). Late Holocene sea- and land-level change on the U.S. southeastern Atlantic coast. Mar. Geol..

[79] Hawkes AD (2016). Relative sea-level change in northeastern Florida (USA) during the last ~8.0 ka. Quat. Sci. Rev..

[80] Gerlach MJ (2017). Reconstructing common era relative sea-level change on the Gulf Coast of Florida. Mar. Geol..

[81] Smerdon JE (2012). Climate models as a test bed for climate reconstruction methods: pseudoproxy experiments. WIREs Clim. Change.

[82] Hughes CW, Meredith MP (2006). Coherent sea-level fluctuations along the global continental slope. Philos. Trans. R. Soc. A.

[83] Frankignoul C, Hasselmann K (1977). Stochastic climate models, part II: application to sea-surface temperature anomalies and thermocline variability. Tellus.

[84] Thompson PR, Mitchum GT (2014). Coherent sea level variability on the North Atlantic western boundary. J. Geophys. Res. Oceans.

[85] Zhao, J. & Johns, W. Wind-forced interannual variability of the Atlantic meridional overturning circulation at 26.5°N. *J*. *Geophys. Res. Oceans* **119**, 2403–2419.

[86] Domingues, R., Baringer, M. & Goni, G. Remote sources for year-to-year changes in the seasonality of the Florida Current transport. *J*. *Geophys. Res. Oceans* **121**, 7547–7559 (2016).

[87] Tingley MP, Huybers P (2010). A Bayesian algorithm for reconstructing climate anomalies in space and time. Part I: development and application to paleoclimate reconstruction problems. J. Clim..

[88] Gelman A, Rubin DB (1992). Inference from iterative simulation using multiple sequences. Stat. Sci..

[89] Tingley MP, Huybers P (2013). Recent temperature extremes at high northern latitudes unprecedented in the past 600 years. Nature.

[90] Taylor KE (2012). An overview of CMIP5 and the experimental design, B. Am. Meteorol. Soc..

[91] Gula J, Molemaker MJ, McWilliams JC (2016). Topographic generation of submesoscale centrifugal instability and energy dissipation. Nat. Commun..

[92] Chepurin GA, Carton JA, Leuliette E (2014). Sea level in ocean reanalyses and tide gauges. J. Geophys. Res. Oceans.

[93] Gent PR (2011). The community climate system model version 4. J. Clim..

[94] Peltier WR (2015). Space geodesy constrains ice age terminal deglaciation: The global ICE-6G_C (VM5a) model. J. Geophys. Res. Solid Earth.

[95] Hamlington BD (2018). Observation-driven estimation of the spatial variability of 20th century sea level rise. J. Geophys. Res. Oceans.

[96] Piecuch CG, Ponte RM (2015). Inverted barometer contributions to recent sea level changes along the northeast coast of North America. Geophys. Res. Lett..

[97] Lumpkin R, Johnson GC (2013). Global ocean surface velocities from drifters: mean, variance, El Niño-Southern Oscillation response, and seasonal cycle. J. Geophys. Res. Oceans.

