# Supplementary material for: Likely weakening of the Florida Current during the past century revealed by sea-level observations
Source: Nat Commun. 2020 Aug 7;11:3973. doi: 10.1038/s41467-020-17761-w (PMC7415143; doi:10.1038/s41467-020-17761-w)
Supplement: Supplementary file 1 — Supplementary Information [file 41467_2020_17761_MOESM1_ESM.pdf]

Supplementary Information for “Likely weakening of the Florida Current during the past century revealed by sea-level observations”

Christopher G. Piecuch

Supplementary Note 1 A note on symbols and notation in this document

In the Methods section, where I develop the Bayesian algorithm, I exhaust most of the letters in the Roman and Greek alphabets. In what follows here, I present more equations, which are informative for interpreting results in the main text. To avoid making these equations cumbersome, I must reuse some of the letters from Methods, but for different purposes. To reduce confusion, I have structured the text of this section and the Methods so that there is no crossover in symbol meaning between the text in the two documents; that is, symbols and letters as defined in the text here are not referred to as such in Methods, and vice versa. The one unavoidable exception is the Supplementary Tables below, some of which (Supplementary Tables 4–8) reference the symbols and letters defined in Methods, and the supplementary figure captions.

Supplementary Note 2 Using the method of surrogate data to evaluate uncertainty on linear trends computed in ancillary datasets accounting for autocorrelation of residuals

At different points in the text, I quote trends computed from ancillary time series not incorporated into the Bayesian model, namely wind-stress curl, sea-surface temperature, and sea-surface height. In these cases, I give the best estimate of the trend followed by a $\pm$ value that represents twice the estimated standard error. Here I describe how the standard errors are estimated.

Typically, when estimating a trend from ordinary least squares, one assumes that residuals are independent and identically distributed (iid) and errors are uncorrelated (e.g., white noise). Yet,

many geophysical time series do not behave as white-noise processes, but rather exhibit temporal autocorrelation[1,2], which is sometimes called long-range dependence or persistence or memory[3–5]. If autocorrelation is not taken into account, then standard errors on trends will be underestimated.

To account for autocorrelation of the residuals, I use the method of surrogate data[6]. Given a data time series $x(t)$, I compute the best estimate of the trend through the data using ordinary least squares. I then remove the trend from the data, leaving the residual series $x'(t)$. Next, I randomly generate a large number (e.g., 10^3) of synthetic time series $\left\{\tilde{x'}_i(t)\right\}$ based on $x'(t)$, such that each $\tilde{x'}_i(t)$ has the same Fourier amplitudes as $x'(t)$ but randomized (scrambled) phase. I compute linear trends in each $\tilde{x'}_i(t)$, resulting in a histogram of the possible apparent trends, or stochastic trends, in a random stationary process with the same basic timescales and amplitudes of variation and the same effective degrees of freedom as the original data series. I take the standard deviation of all of the $\tilde{x'}_i(t)$ trends as the estimated standard error on the original $x(t)$ trend.

Supplementary Note 3 Simulating the hypothetical transport of the Antilles Current

The question arose in the text as to whether the probable weakening of the Florida Current transport over the past century is partly balanced by compensating changes in the Antilles Current transport. Direct measurements of the Antilles Current are short and do not allow for a direct observational assessment. However, it is possible to estimate a range of possible stochastic (or random) transport trends, given the time-series properties of the available data.

Meinen et al.[7] report, based on $\sim$ 11 y of daily data (2005–2015), that the Antilles Current

transport has a standard deviation of 7.5 Sv and an integral timescale of 19.0 days (cf. their Table 3). Following Emery and Thomson[8], the integral timescale τ of a discrete time series is defined as,

$$\tau = 2\Delta t \sum_{k=0}^{+\infty} C_k, \tag{S1}$$

where $C_k = C\left(k\Delta t\right)$ is the autocorrelation function of the time series with itself at lag $k\Delta t$ for a time increment Δt. These statistics form the basis of simple simulation experiments of the Antilles Current transport.

I assume that Antilles Current transport behaves as random stationary red noise that can be modeled as an autoregressive process of order 1,

$$y_k = \varphi y_{k-1} + \varepsilon_k. \tag{S2}$$

Here y_k is Antilles Current transport at time step k, φ is the lag-1 autocorrelation coefficient, and $\varepsilon_k \sim \mathcal{N}\left(0, \sigma^2\right)$ is stationary white noise with zero mean and variance σ^2. To simulate this process, values for φ and σ^2 must be assigned based on the Antilles Current transport observations.

Based on the properties of an autoregressive processes, the autocorrelation of y_k is $C_k = \varphi^k$. Using this form of C_k in Eq. $(S1)$ and evaluating the sum of the geometric series gives,

$$\tau = \frac{2\Delta t}{1 - \varphi}. \tag{S3}$$

Rearranging to solve for φ, and using $\tau = 19.0 \text{ days}$ and $\Delta t = 1 \text{ day}$ from Meinen et al.[7], gives,

$$\varphi = 1 - \frac{2\Delta t}{\tau} = 0.9. \tag{S4}$$

The variance of y_k is $\mathrm{var}(y_k) = \sigma^2 / (1 - \varphi^2)$. Rearranging to solve for σ^2, setting $\varphi = 0.9$ based on Eq. ($S4$), and using $\mathrm{var}(y_k) = (7.5\ \mathrm{Sv})^2$ from Meinen et al.[7] gives,

$$\sigma^2 = \left(1 - \varphi^2\right) \mathrm{var}(y_k) = (3.3\ \mathrm{Sv})^2. \tag{S5}$$

Using these values for φ and σ^2, I run simulation experiments to quantify the possible range of stochastic trends in Antilles Current transport as a function of timescale. For time-series lengths between 1 and 150 y, I generate random values for ε_k in Eq. ($S2$) to yield 1,000 separate synthetic daily series of surrogate Antilles Current transport. I compute the linear trend in each of these 1,000 surrogate time series for each specified time-series length. This allows me to populate a histogram of the trends possible for a stationary random red-noise process with the same variance and integral timescale as the Antilles Current transport data. Shading in Supplementary Figure 11 represents the 95% confidence interval (2.5th and 97.5th percentiles) from these simulations as a function of timescale, which are ± 2.9 and ± 1.2 Sv century^{-1} for periods of 50- and 100-y, respectively.

Supplementary Note 4 Computing the trend in surface heat flux implied by a trend in sea-surface temperature

What follows in this section is based on the forms of air-sea fluxes described by Large and Yeager[9] (their section 2.1). Most symbols used in this section are theirs.

I start by establishing basic definitions, after Large and Yeager[9]. The total air-sea heat flux

Q_{as} (positive into the ocean) is given by a sum of contributions,

$$Q_{as} = Q_S + Q_L + Q_E + Q_H, \tag{S6}$$

where Q_S is shortwave solar radiation,

$$Q_S = Q_I \left(1 - \alpha\right), \tag{S7}$$

where Q_I is the insolation and α is the surface albedo; Q_L is the net longwave flux,

$$Q_L = Q_A - \sigma \left(\mathrm{SST}\right)^4, \tag{S8}$$

where Q_A is the longwave energy received from the atmosphere, SST is sea-surface temperature, and $\sigma = 5.67 \times 10^{-8}$ W m^{-2} K^{-4} is the Stefan-Boltzmann constant; Q_E is the latent turbulent flux,

$$Q_E = \Lambda_v \rho_a C_E \left[q\left(z_q\right) - q_{sat}\left(\mathrm{SST}\right)\right] \left|\Delta\vec{U}\right|, \tag{S9}$$

where $\Lambda_v = 2.5 \times 10^6$ J kg^{-1} is the latent heat of vaporization, $\rho_a = 1.22$ kg m^{-3} is air density near the surface, $q\left(z_q\right)$ is the specific humidity of air at a height z_q above the surface, C_E is the transfer coefficient for evaporation, q_{sat} is the specific humidity of air at saturation, and $\left|\Delta\vec{U}\right|$ is the near-surface wind speed; and Q_H is the sensible turbulent flux,

$$Q_H = \rho_a c_p C_H \left[\theta\left(z_\theta\right) - \mathrm{SST}\right] \left|\Delta\vec{U}\right|, \tag{S10}$$

where $c_p = 1000.5$ J kg^{-1} K^{-1} is the specific heat of air, C_H is the transfer coefficient of sensible heat, and $\theta\left(z_\theta\right)$ is air temperature at a height z_θ above the surface. Note that I ignore heat flux due to precipitation, since its contribution is often small and uncertain.

The transfer coefficients of latent and sensible heat are functions of the drag coefficient C_D,

$$C_E = \frac{34.6}{1000}\sqrt{C_D}, \tag{S11}$$

and

$$C_H = \frac{18.0}{1000}\sqrt{C_D}, \tag{S12}$$

with

$$C_D = \frac{2.70 \text{ m s}^{-1}}{U_N\,(10\text{ m})} + 0.142 + \frac{U_N\,(10\text{ m})}{13.09 \text{ m s}^{-1}}, \tag{S13}$$

where $U_N\,(10\text{ m})$ is variable wind speed at 10 m under neutral stability. Note that I assume stability in defining C_H in Eq. ($S12$); see Large and Yeager[9] for more details on forms of C_H.

The specific humidity at saturation q_{sat} is a function of SST,

$$q_{sat}\,(q_1, q_2, \text{SST}) = \frac{q_1}{\rho_a}\exp\left(\frac{q_2}{\text{SST}}\right), \tag{S14}$$

where $q_1 = 0.98 \times 640380$ kg m^{-3} and $q_2 = -5107.4$ K are the coefficients.

All of the above is as in Large and Yeager[9]. I now use these forms to consider an infinitesimal perturbation dSST in sea-surface temperature. The resulting perturbation dQ_{as} in surface heat flux is exactly,

$$dQ_{as} = \left.\frac{\partial Q_{as}}{\partial \text{SST}}\right|_{\overline{\text{SST}}} d\text{SST} = \left(\left.\frac{\partial Q_L}{\partial \text{SST}}\right|_{\overline{\text{SST}}} + \left.\frac{\partial Q_E}{\partial \text{SST}}\right|_{\overline{\text{SST}}} + \left.\frac{\partial Q_H}{\partial \text{SST}}\right|_{\overline{\text{SST}}}\right) d\text{SST}, \tag{S15}$$

where $|_{\overline{\text{SST}}}$ indicates that values are evaluated at the background average SST value. Contributions from Q_S fall away because they have no explicit dependence on SST. The partial derivatives on the right-hand side of Eq. ($S15$) are,

$$\left.\frac{\partial Q_L}{\partial \text{SST}}\right|_{\overline{\text{SST}}} = -4\sigma\left(\overline{\text{SST}}\right)^3, \tag{S16}$$

$$\left.\frac{\partial Q_E}{\partial \mathrm{SST}}\right|_{\overline{\mathrm{SST}}} = \Lambda_v \rho_a C_E \big|\Delta \vec{U}\big| \frac{q_2}{\overline{\mathrm{SST}}^2} q_{sat}\left(\overline{\mathrm{SST}}\right), \tag{S17}$$

and

$$\left.\frac{\partial Q_H}{\partial \mathrm{SST}}\right|_{\overline{\mathrm{SST}}} = -\rho_a c_p C_H \big|\Delta \vec{U}\big|, \tag{S18}$$

whence,

$$dQ_{as} = \underbrace{\left[\Lambda_v \rho_a C_E \big|\Delta \vec{U}\big| \frac{q_2}{\overline{\mathrm{SST}}^2} q_{sat}\left(\overline{\mathrm{SST}}\right) - 4\sigma\left(\overline{\mathrm{SST}}\right)^3 - \rho_a c_p C_H \big|\Delta \vec{U}\big|\right]}_{\Gamma} d\mathrm{SST}. \tag{S19}$$

For a finite but small, linear SST perturbation, Eq. ($S19$) will hold approximately. Therefore, I use Eq. ($S19$) to estimate the Q_{as} trend implied by the SST trend observed over the warming hole. Values for Λ_v, ρ_a, q_1, q_2, σ, and c_p are given above. Based on examination of a global oceanic state estimate[10], I determine that $C_D = 0.0011$, $C_E = 0.0012$, and $C_H = 0.00061$ are reasonable values for the transfer coefficients over the subpolar gyre. Judging from this state estimate, I also assume reasonable distributions $\overline{\mathrm{SST}} \sim \mathcal{U}\left(6.5°\mathrm{C}, 11.5°\mathrm{C}\right)$ and $\big|\Delta \vec{U}\big| \sim \mathcal{U}\left(7.5\ \mathrm{m\ s^{-1}}, 10.5\ \mathrm{m\ s^{-1}}\right)$ for the subpolar gyre, where $\mathcal{U}\left(a, b\right)$ is the uniform distribution with minimum a and maximum b. Using these values, I estimate an approximate 95% confidence interval for the bracketed term (Γ) on the right-hand side of Eq. ($S19$) of -27 ± 4.6 W m^{-2} °C^{-1}. Multiplying this estimate of Γ times the trend in SST over the subpolar warming hole quoted in the main text (-0.6 ± 0.4 °C century^{-1}) produces the surface heat flux trend of 16 ± 11 W m^{-2} century^{-1} given in the text.

Supplementary Note 5 Converting a trend in surface heat flux over the subpolar warming hole to a temperature acceleration over the northern North Atlantic and Arctic Oceans

From basic conservation principles, if a surface heat flux Q_{as} acts on the ocean surface, and all of the heat gained is stored locally in the ocean, then the heat budget is,

$$\frac{\partial \Theta}{\partial t} = \frac{1}{\rho C_p H} Q_{as}, \tag{S20}$$

where Θ is the depth-averaged ocean (potential) temperature, ρ is seawater density, C_p is the specific heat of seawater (distinct from c_p, which is the specific heat of air in the past section), and H is the depth of the water column. Equivalently, taking a time derivative,

$$\frac{\partial^2 \Theta}{\partial t^2} = \frac{1}{\rho C_p H} \frac{\partial Q_{as}}{\partial t}. \tag{S21}$$

Hence, the right-hand side takes the form of a temperature acceleration. Setting $\partial Q_{as} \big/ \partial t$ equal to 16 ± 11 W m^{-2} century^{-1} for the warming-hole region from the previous section, choosing typical round numbers of $\rho = 10^3$ kg m^{-3} and $C_p = 4 \times 10^3$ J kg^{-1} °C^{-1}, and selecting $H = 2.5 \times 10^3$ m as a representative depth for the northern North Atlantic Ocean, I obtain a range for the temperature acceleration $\partial^2 \Theta \big/ \partial t^2$ of 5.0 ± 3.5 °C century^{-2}, which equates to a warming of 3.1 ± 2.1 °C over a 110-y period.

These acceleration and warming numbers apply to the subpolar warming-hole region, which has a surface area of about 5.3×10^{12} m^2 (Figure 7). In other words, these are the ocean temperature changes that would be experienced in that region due to the surface heat flux in the absence of any

lateral redistribution of heat by circulation and mixing. However, given the focus of this paper, it is instructive to consider whether the heat gain is stored not locally over the warming hole, but rather more broadly across the northern North Atlantic and Arctic Ocean, from 27°N to Bering Strait. The motivation for choosing 27°N as a boundary of the control volume is obvious, given the focus of the paper at that latitude. Bering Strait is typically chosen a convenient boundary to close the control volume in studies of Atlantic Ocean heat transport and storage, since transports of mass, heat, and freshwater through Bering Strait are relatively small and stable compared to transports in the Atlantic[11,12]. (Here I assume changes in transports through Bering Strait are totally negligible.) The surface area of this larger North Atlantic and Arctic control volume is 3.6×10^{13} m^2. Hence, multiplying the values above by the ratio of surface areas ($5.3 \times 10^{12}\ \mathrm{m}^2 / 3.6 \times 10^{13}\ \mathrm{m}^2 = 0.15$), the temperature acceleration and overall warming from earlier become 0.7 ± 0.5 °C century^{-2} and 0.4 ± 0.3 °C, respectively, if the heat flux over the warming hole was redistributed evenly over the northern North Atlantic and Arctic. These are the values quoted in the main text.

Supplementary Note 6 Estimating trends in the deep branch of the overturning circulation and thermocline recirculation from trends in the Florida Current and subpolar sea-surface temperature

In the main text, I explain that the weakening of Florida Current transport and the surface heat flux trend resulting from the cooling of subpolar sea-surface temperatures over the warming hole must be physically consistent with two simple conservation principles: the sum of changes in all volume transports at 27°N must equal zero (mass conservation), and the trend in ocean heat transport across

27°N must match to lowest order the trend in surface heat flux over the warming-hole region (heat conservation). I express these requirements for mass and heat conservation respectively as,

$$\psi_F' + \psi_D' + \psi_T' = 0, \tag{S22}$$

and

$$-\psi_F'\overline{\Theta}_F - \psi_D'\overline{\Theta}_D - \psi_T'\overline{\Theta}_T = \frac{A}{C_p\rho}Q_{as}'. \tag{S23}$$

Here ψ_F, ψ_D, and ψ_T are the volume transports across 27°N (positive northward) by the Florida Current, the deep branch of the overturning circulation, and thermocline recirculation, respectively, and Θ_F, Θ_D, and Θ_T are corresponding representative ocean temperatures in Florida Straits, the deep ocean ($\gtrsim$ 1000 m), and the interior upper ocean ($\lesssim$ 1000 m), respectively. As before, Q_{as} is surface heat flux over the warming hole, ρ ocean density, and C_p specific heat capacity of seawater, and here A is the surface area of the warming hole, so that AQ_{as} is the total surface heating of the control volume. Primes are used here to indicate linear trends whereas overbars represent time means. Note that in Eq. ($S23$), I ignore the time-tendency (local storage) term and heat transport by currents acting on temperature anomalies. These assumptions are discussed in more detail below.

This linear system can be rearranged to solve for ψ_D' and ψ_T' in terms of ψ_F' and SST', *viz.*,

$$\psi_D' = \frac{\overline{\Theta}_F - \overline{\Theta}_T}{\overline{\Theta}_T - \overline{\Theta}_D}\psi_F' + \frac{1}{\overline{\Theta}_T - \overline{\Theta}_D}\frac{A\Gamma}{C_p\rho}\mathrm{SST}', \tag{S24}$$

and,

$$\psi_T' = -\frac{\overline{\Theta}_F - \overline{\Theta}_D}{\overline{\Theta}_T - \overline{\Theta}_D}\psi_F' - \frac{1}{\overline{\Theta}_T - \overline{\Theta}_D}\frac{A\Gamma}{C_p\rho}\mathrm{SST}', \tag{S25}$$

where Eq. ($S19$) was used to substitute Γ SST' for Q_{as}'. Based on examination of climatological

temperature from the World Ocean Atlas[13] along 27°N (Supplementary Figure 14), I assume that $\overline{\Theta}_F \sim \mathcal{U}\,(14.2°\text{C}, 20.5°\text{C})$, $\overline{\Theta}_T \sim \mathcal{U}\,(12.2°\text{C}, 16.9°\text{C})$, and $\overline{\Theta}_D \sim \mathcal{U}\,(1.4°\text{C}, 3.6°\text{C})$.

Given the $\overline{\Theta}_F$, $\overline{\Theta}_T$, and $\overline{\Theta}_D$ distributions, and values of $A = 5.3 \times 10^{12}$ m^2, $\rho = 10^3$ kg m^{-3}, $C_p = 4 \times 10^3$ J kg^{-1} °C^{-1}, and $\Gamma = -27 \pm 4.6$ W m^{-2} °C^{-1} from before, I can evaluate Eqs. ($S24$), ($S25$). Using the values of $\psi'_F = -1.7 \pm 3.7$ Sv century^{-1} and $\text{SST}' = -0.6 \pm 0.4$ °C century^{-1} presented and discussed in the main text for 1909–2018, I find $\psi'_D = 1.4 \pm 1.8$ Sv century^{-1} and $\psi'_T = -0.3 \pm 4.9$ Sv century^{-1}, which are also quoted in the main text. More general estimates of ψ'_D and ψ'_T through time, and shown in Supplementary Figure 13, are generated by computing the posterior median and 95% credible interval of the mean rate of change in Florida Current transport from the Bayesian model and the linear trend in sea-surface temperature over the warming hole for all periods starting between 1909 and 1980 and ending in 2018, and then applying those values for the mean rates of change and linear trends to Eqs. ($S24$), ($S25$).

Some caveats should be discussed. The form of heat conservation ($S23$) assumes that (1.) the time rate of change of temperature is negligible and (2.) heat transport due to currents acting on variable temperatures can be ignored.

Assumption (1.), that local ocean heat storage is negligible, was mentioned in the main text. I argued, using available estimates of ocean warming, that changes in local ocean heat content are too small to account for the surface heat flux implied by the longterm changes in sea-surface temperature, and therefore the surface heat flux must be mostly balanced by ocean heat transport. Note that, while it is reasonable on long, multidecadal to centennial timescales, assumption (1.)

does not apply on shorter, interannual and decadal periods, when changes in local ocean heat content become relatively more important. For example, over 1993–2015, decadal changes in subpolar North Atlantic Ocean heat content largely balanced ocean heat transport into the region due to changes in ocean circulation, and changes in surface heat flux were relatively small in comparison[14]. For this reason, Eqs. ($S24$) and ($S25$) are not evaluated for $\lesssim 40$-y periods.

Assumption (2.), that circulation changes operating on mean temperatures make the leading contributions to the heat transport, was not discussed in the text. This assumption has precedent in the observational literature and is supported by modeling results. For example, ocean heat transport estimates based on data from the RAPID array at $26°$N have largely ignored nonseasonal changes in ocean temperature[15–17]. This assumption has been justified from strong correlation found between cable-based volume transports and directly measured temperature transports in Florida Straits[15,18], for example. Models generally support this result. Across a range of timescales, from seasonal to decadal, modeled ocean heat transports in the subtropical North Atlantic are strongly determined by changes in circulation acting on time-mean ocean temperatures, though time variations in temperature are not totally negligible[19–21].

These arguments make the case that assumptions (1.) and (2.) are reasonable and justifiable in the context of the back-of-the-envelope, order-of-magnitude estimates presented here. However, they are simplifications, as acknowledged above. Future studies should revisit these topics in more detail (e.g., relative importance of local ocean heat storage versus surface heat flux and ocean heat transport, or temperature versus velocity effects on ocean heat transports as a function of timescale)

based on models. However, such in-depth analyses are beyond the scope of the simple calculations presented here for purposes of interpreting posterior solutions from the Bayesian model, and so are left to more focused future studies.

Supplementary References

1. Bos, M. S., Williams, S. D. P., Araújo, I. B., & L. Bastos. The effect of temporal correlated noise on the sea level rate and acceleration uncertainty, *Geophys. J. Int.*, **196**, 1423–1430.

2. Frankignoul, C., & Hasselmann, K. Stochastic climate models, part II: application to sea-surface temperature anomalies and thermocline variability, *Tellus*, **29**, 289–305 (1977).

3. Dangendorf, S., Rybski, D., Mudersbach, C., Müller, A., Kaufmann, E., Zorita, E., & Jensen, J. Evidence for long-term memory in sea level, *Geophys. Res. Lett.*, **41**, 5530–5537 (2014).

4. Fatichi, S., Barbosa, S. M., Caporali, E., & Silva, M. E. Deterministic versus stochastic trends: Detection and challenges, *J. Geophys. Res.*, **114**, D18121 (2009).

5. Ocaña, V., Zorita, E., & Heimbach, P. Stochastic secular trends in sea level rise, *J. Geophys. Res.-Oceans*, **121**, 2183–2202 (2016).

6. Theiler, J., Eubank, S., Longtin, A., Galdrikian, B., & Farmer, J. D. Testing for nonlinearity in time series: the method of surrogate data, *Physica D*, **58**, 77–94 (1992).

7. Meinen, C. S., Johns, W. E., Moat, B. I., Smith, R. H., Johns, E. M., Rayner, D., Frajka-Williams, E., Garcia, R. F., & Garzoli, S. L. Structure and variability of the Antilles Current at $26.5°$N, *J. Geophys. Res.-Oceans*, **124**, 3700–3723 (2019).

8. Emery, R. E., & Thomson, W. J. Data analysis methods in physical oceanography, 3rd Edition, Elsevier, 728 pp (2014).

9. Large, W. G., & Yeager, S. G. Diurnal to decadal global forcing for ocean and sea-ice models: the data sets and flux climatologies, *NCAR Technical Note*, NCAR/TN-460+STR, 112 pp.

10. Forget, G., et al. ECCO version 4: an integrated framework for non-linear inverse modeling and global ocean state estimation, *Geosci. Model Dev.*, **8**, 3071–3104 (2015).

11. Roach, A. T., Aagaard, K., Pease, C. H., Salo, S. A., Weingartner, T., Pavlov, V., & Kulakov, M. Direct measurements of transport and water properties through the Bering Strait, *J. Geophys. Res.*, **100**(C9), 18443–18457 (1995).

12. Woodgate, R. A. Increases in the Pacific inflow to the Arctic from 1990 to 2015, and insights into seasonal trends and driving mechanisms from year-round Bering Strait mooring data, *Prog. Oceanogr.*, **160**, 124–154 (2018).

13. Locarni, R. A., Mishonov, A. V., Baranova, O. K., Boyer, T. P., Zweng, M. M., Garcia, H. E., Reagan, J. R., Seidov, D., Weathers, K., Paver, C. R., & Smolyar, I. World Ocean Atlas 2018, Volume 1: Temperature, NOAA Atlas NESDIS 81, 52 pp (2018).

14. Piecuch, C. G., Ponte, R. M., Little, C. M., Buckley, M. W., & Fukumori, I. Mechanisms underlying recent decadal changes in subpolar North Atlantic Ocean heat content, *J. Geophys. Res.-Oceans*, **122**, 7181–7197 (2017).

15. Johns, W. E., Baringer, M. O., Beal, L. M., Cunningham, S. A., Kanzow, T., Bryden, H. L., Hirschi, J. J. M., Marotzke, J., Meinen, C. S., Shaw, B., & Curry, R. Continuous, array-based estimates of Atlantic Ocean heat transport at $26.5°$N, *J. Clim.*, **24**, 2429–2449 (2011).

16. Bryden, H. L., King, B. A., McCarthy, G. D., & McDonagh, E. L. Impact of a 30% reduction in Atlantic meridional overturning 2009–2010, *Ocean Sci.*, **10**, 683–691 (2014).

17. Bryden, H. L., Johns, W. E., King, B. A., McCarthy, G., McDonagh, E. L., Moat, B. I., & Smeed, D. A. Reduction in ocean heat transport at $26°$N since 2008 cools the eastern subpolar gyre of the North Atlantic Ocean, *J. Clim.*, **33**, 1677–1689 (2020).

18. Shoosmith, D. R., Baringer, M. O., & Johns, W. E. A continuous record of Florida Current temperature transport at $27°$N, *Geophys. Res. Lett.*, **32**, L23603 (2005).

19. Jayne, S. R., & Marotzke, J. The dynamics of ocean heat transport variability, *Rev. Geophys*, **39**, 385–411 (2001).

20. Dong, B., & Sutton, R. T. Variability in North Atlantic heat content and heat transport in a coupled ocean-atmosphere GCM, *Clim. Dynam.*, **19**, 485–497 (2002).

21. Piecuch, C. G., & Ponte, R. M. Importance of circulation changes to Atlantic heat storage rates on seasonal and interannual time scales, *J. Clim.*, **25**, 350–362 (2012).

22. Santamaría-Gómez, A., et al. Uncertainty of the 20th century sea-level rise due to vertical land motion errors, *Earth Planet. Sci. Lett.*, **473**, 24–32 (2017).

23. Love, R., et al. The contribution of glacial isostatic adjustment to projections of sea-level change along the Atlantic and Gulf coasts of North America, *Earth's Future*, **4**, 440–464 (2016).

24. Khan, N. S., et al. Drivers of Holocene sea-level change in the Caribbean, *Quaternary Sci. Rev.*, **155**, 13–36 (2017).

25. Gelman, A, & Rubin, D. B. Inference from iterative simulation using multiple sequences, *Stat. Sci.*, **7**, 457–472 (1992).

26. Birol, F., et al. Coastal applications from nadir altimetry: Example of the X-TRACK regional products, *Adv. Space Res.*, **59**, 936–953 (2017).

27. Gebbie, G., & Huybers, P. The Little Ice Age and 20th-century deep Pacific cooling, *Science*, **363**, 70–74 (2019).

Region	Site	Lon (°W)	Lat (°N)	Duration (years)	Rate (mm y^{-1})	Error (mm y^{-1})
Florida	AOML	80.1622	25.7347	6.37	0.27	0.74
Florida	CCV6	80.5455	28.4600	6.93	-2.95	0.74
Florida	MIA3	80.1602	25.7328	11.00	-0.32	0.80
Bahamas	EXU0	75.8734	23.5640	6.50	-1.85	0.54
Bahamas	NAS0	77.4623	25.0525	6.51	-2.18	2.42

Supplementary Table 1. Summary of GPS data from Version 6b of the dataset from Université de la Rochelle[22] used to estimate the difference in static sea-level rate across Florida Straits due to differential land motion quoted in the main text. Duration is the length of the data record. Error is twice the formal standard error provided with the dataset. Assuming errors are independent, the average rate across the two Bahamas sites is -2.02 ± 1.24 mm y^{-1} and the average rate across the three southeastern Florida sites is -1.00 ± 0.44 mm y^{-1}. The difference between the former and latter average values is -1.02 ± 1.32 mm y^{-1}, which represents the rate of differential vertical land motion across Florida Straits quoted in the main text. Multiplying by -1 to convert from the land-motion frame to the sea-level frame gives the value of 1.0 ± 1.3 mm y^{-1} quoted in the main text.

Region	Site	Reference	Lon (°W)	Lat (°N)	age (y BP)	sea level (m)
Florida	Florida Bay	Love et al.[23]	80.6	25	1260 ± 275	-1.34 ± 1.27
					890 ± 290	-0.83 ± 1.39
					400 ± 335	-1.00 ± 1.26
Florida	Bear Point	Love et al.[23]	80.3	27.4	1930 ± 350	-0.93 ± 1.45
					1380 ± 225	-1.13 ± 1.45
					1120 ± 215	-0.83 ± 1.45
Bahamas	Acklins Island	Khan et al.[24]	73.9	22.5	1048 ± 490	-1.64 ± 1.14
					698 ± 392	-1.23 ± 1.26
					398 ± 500	-1.08 ± 1.22
					242 ± 484	-0.97 ± 1.18

Supplementary Table 2. Proxy sea-level index points from southeastern Florida and the Bahamas used to estimate the difference in the rate of late-Holocene sea-level change across Florida Straits quoted in the main text. Latitudes and longitudes have been rounded to the nearest tenth of a degree. The "y BP" abbreviation stands for years before present, where present is 1950. The $\pm$ values are twice the standard errors on the age and sea-level values provided in the given references. Using ordinary least squares to fit a trend line to the index points at each site, and ignoring age and sea-level uncertainty, I compute trends of 0.36 ± 0.97, 0.05 ± 0.73 and 0.81 ± 0.22 mm y^{-1} at Florida Bay, Bear Point, and Acklins Island, respectively, where $\pm$ is twice the formal standard error furnished by ordinary least squares assuming independent data. The average of the two trends from southeastern Florida is thus 0.20 ± 0.61 mm y^{-1} and so the difference between the Bahamas and southeastern Florida is 0.6 ± 0.6, which is the value quoted in the main text.

No.	Location	Lon (°E)	Lat (°N)	Timespan (Completeness)	Coast
1	Cristóbal	-79.9167	9.35	1909–1979 (100%)	904
2	Puerto Limon	-83.0333	10	1949–1968 (90%)	906
3	Cartagena	-75.55	10.4	1949–1992 (68%)	902
4	Riohacha	-72.9167	11.55	1953–1969 (82%)	902
5	Fort-de-France II	-61.0632	14.6015	2006–2017 (100%)	912
6	Santo Tomás de Castilla	-88.6167	15.7	1965–1980 (75%)	916
7	Puerto Cortes	-87.95	15.8333	1948–1968 (100%)	908
8	Puerto Castilla	-86.0333	16.0167	1956–1968 (100%)	908
9	Lime Tree Bay	-64.7533	17.6933	1986–2015 (80%)	939
10	Port Royal	-76.85	17.9333	1955–1969 (100%)	932
11	Magueyes Island	-67.045	17.97	1955–2016 (90%)	938
12	Barahona	-71.0833	18.2	1955–1969 (67%)	936
13	Charlotte Amalie	-64.92	18.335	1976–2016 (61%)	939
14	San Juan	-66.115	18.4583	1963–2016 (81%)	938
15	Port-au-Prince	-72.35	18.5667	1950–1961 (100%)	934
16	South Sound	-81.3833	19.2667	1976–1993 (89%)	931
17	North Sound	-81.3167	19.3	1976–1996 (86%)	931
18	Puerto Plata	-70.7	19.8167	1950–1969 (70%)	936
19	Cabo Cruz	-77.7333	19.8333	1993–2017 (76%)	930
20	Guantanamo Bay	-75.1467	19.9067	1938–1971 (85%)	930
21	Gibara	-76.125	21.1083	1976–2016 (100%)	930
22	Nuevitas Punta Practico	-77.1095	21.5913	1992–2017 (35%)	930
23	Casilda II	-79.9917	21.7533	1984–2014 (48%)	930
24	Cabo de San Antonio	-84.9	21.9	1973–2017 (60%)	930
25	Isabela de Sagua	-80.0167	22.9333	2000–2016 (71%)	930
26	Key West	-81.8067	24.555	1913–2018 (97%)	940
27	Vaca Key	-81.105	24.7117	1990–2017 (79%)	940
28	Key Colony Beach	-81.0167	24.7183	1978–1994 (71%)	940
29	Virginia Key	-80.1617	25.73	1995–2017 (87%)	960
30	Miami Beach	-80.1317	25.7683	1932–1980 (92%)	960
31	Naples	-81.8067	26.1317	1966–2017 (83%)	940
32	West Palm Beach	-80.0333	26.6117	1974–2017 (36%)	960
33	Settlement Point	-78.9833	26.6833	2005–2015 (82%)	941
34	Settlement Point	-78.9967	26.71	1986–2000 (67%)	941
35	Trident Pier	-80.5917	28.415	1995–2017 (91%)	960
36	Daytona Beach Shores	-80.9633	29.1467	1967–1983 (71%)	960
37	Daytona Beach	-81	29.2333	1925–1969 (51%)	960
38	Jacksonville	-81.6167	30.35	1954–1967 (100%)	960
39	Mayport	-81.4317	30.3933	1929–1999 (99%)	960
40	Mayport	-81.4283	30.3983	2001–2017 (94%)	960
41	Fernandina Beach	-81.465	30.6717	1909–2018 (78%)	960
42	Fort Pulaski	-80.9017	32.0333	1935–2018 (95%)	960
43	Charleston	-79.925	32.7817	1922–2018 (100%)	960
44	Springmaid Pier	-78.9183	33.655	1978–2017 (60%)	960
45	Myrtle Beach	-78.885	33.6833	1958–1977 (55%)	960
46	Wilmington	-77.9533	34.2267	1936–2018 (95%)	960

Supplementary Table 3. Descriptions of tide-gauge sea-level records used in this study. "Completeness" is the percentage of timespan during which data are available. "Coast" number is the code used by the PSMSL to indicate the country and coastline of measurement.

Parameter	Description
$\boldsymbol{\eta}_0$	Sea-level initial condition
$\boldsymbol{\eta}_k$	Sea-level values at time t_k
$\overline{T}$	Transport time-mean value
T_k	Transport value at time t_k
$\boldsymbol{b}$	Spatial vector of regional trends in sea level
$\boldsymbol{a}$	Spatial vector of local trends in sea level
$\boldsymbol{\ell}$	Spatial vector of tide-gauge biases
r	AR(1) coefficient of sea level
μ	Mean value of regional trends in sea level
ν	Mean value of tide-gauge biases
ρ	Transport change per unit sea-level difference
α	Transport trend correction
π^2	Partial sill of regional trends in sea level
σ^2	Partial sill of sea-level innovations
δ^2	Tide-gauge error variance
τ^2	Spatial variance in observational biases
γ^2	Variance of local trends in sea level
ω^2	Variance of transport noise correction
ϕ	Inverse range of sea-level innovations
λ	Inverse range of regional trends in sea level

Supplementary Table 4. Descriptions of model processes and parameters.

Parameter	Prior Distribution	Hyperparameter Values
$\boldsymbol{\eta}_0$	$\mathcal{N}\left(\tilde{\eta}_{\boldsymbol{\eta}_0}\mathbf{1}, \tilde{\zeta}^2_{\boldsymbol{\eta}_0}\mathsf{I}\right)$	$\tilde{\eta}_{\boldsymbol{\eta}_0} = -0.2$ m , $\tilde{\zeta}^2_{\boldsymbol{\eta}_0} = (7.6 \times 10^{-2}$ m$)^2$
$\overline{T}$	$\mathcal{N}\left(\tilde{\eta}_{\overline{T}}, \tilde{\zeta}^2_{\overline{T}}\right)$	$\tilde{\eta}_{\overline{T}} = 32$ Sv , $\tilde{\zeta}^2_{\overline{T}} = (5.2$ Sv$)^2$
r	$\mathcal{U}\left(\tilde{u}_r, \tilde{v}^2_r\right)$	$\tilde{u}_r = 0.0$, $\tilde{v}^2_r = 1.0$
μ	$\mathcal{N}\left(\tilde{\eta}_\mu, \tilde{\zeta}^2_\mu\right)$	$\tilde{\eta}_\mu = 3.4 \times 10^{-3}$ m y^{-1} , $\tilde{\zeta}^2_\mu = (2.7 \times 10^{-2}$ m y$^{-1})^2$
ν	$\mathcal{N}\left(\tilde{\eta}_\nu, \tilde{\zeta}^2_\nu\right)$	$\tilde{\eta}_\nu = 7.0$ m , $\tilde{\zeta}^2_\nu = (0.6$ m$)^2$
ρ	$\mathcal{N}\left(\tilde{\eta}_\rho, \tilde{\zeta}^2_\rho\right)$	$\tilde{\eta}_\rho = 0.0$ Sv m^{-1} , $\tilde{\zeta}^2_\rho = (190$ Sv m$^{-1})^2$
α	$\mathcal{N}\left(\tilde{\eta}_\alpha, \tilde{\zeta}^2_\alpha\right)$	$\tilde{\eta}_\alpha = 0.0$ Sv y^{-1} , $\tilde{\zeta}^2_\alpha = (0.3$ Sv y$^{-1})^2$
π^2	$\mathcal{IG}\left(\tilde{\xi}_{\pi^2}, \tilde{\chi}^2_{\pi^2}\right)$	$\tilde{\xi}_{\pi^2} = 0.5$, $\tilde{\chi}^2_{\pi^2} = (1.9 \times 10^{-3}$ m y$^{-1})^2$
σ^2	$\mathcal{IG}\left(\tilde{\xi}_{\sigma^2}, \tilde{\chi}^2_{\sigma^2}\right)$	$\tilde{\xi}_{\sigma^2} = 0.5$, $\tilde{\chi}^2_{\sigma^2} = (1.8 \times 10^{-2}$ m$)^2$
δ^2	$\mathcal{IG}\left(\tilde{\xi}_{\delta^2}, \tilde{\chi}^2_{\delta^2}\right)$	$\tilde{\xi}_{\delta^2} = 0.5$, $\tilde{\chi}^2_{\delta^2} = (7.1 \times 10^{-3}$ m$)^2$
τ^2	$\mathcal{IG}\left(\tilde{\xi}_{\tau^2}, \tilde{\chi}^2_{\tau^2}\right)$	$\tilde{\xi}_{\tau^2} = 0.5$, $\tilde{\chi}^2_{\tau^2} = (8.5 \times 10^{-2}$ m$)^2$
γ^2	$\mathcal{IG}\left(\tilde{\xi}_{\gamma^2}, \tilde{\chi}^2_{\gamma^2}\right)$	$\tilde{\xi}_{\gamma^2} = 0.5$, $\tilde{\chi}^2_{\gamma^2} = (7.1 \times 10^{-4}$ m y$^{-1})^2$
ω^2	$\mathcal{IG}\left(\tilde{\xi}_{\omega^2}, \tilde{\chi}^2_{\omega^2}\right)$	$\tilde{\xi}_{\omega^2} = 0.5$, $\tilde{\chi}^2_{\omega^2} = (0.7$ Sv$)^2$
ϕ	$\mathcal{LN}\left(\tilde{\eta}_\phi, \tilde{\zeta}^2_\phi\right)$	$\tilde{\eta}_\phi = -7.0$ log km^{-1} , $\tilde{\zeta}^2_\phi = (2.2$ log km$^{-1})^2$
λ	$\mathcal{LN}\left(\tilde{\eta}_\lambda, \tilde{\zeta}^2_\lambda\right)$	$\tilde{\eta}_\lambda = -6.9$ log km^{-1} , $\tilde{\zeta}^2_\lambda = (0.4$ log km$^{-1})^2$

Supplementary Table 5. Prior distributions and hyperparameters. Hyperparameters are denoted with tildes to distinguish them from the other (uncertain) model parameters. The scripts are: $\mathcal{N}$ normal (or multivariate normal) distribution with mean $\tilde{\eta}$ and variance $\tilde{\zeta}^2$; $\mathcal{U}$ uniform distribution with lower bound $\tilde{u}$ and upper bound $\tilde{v}$; $\mathcal{IG}$ inverse-gamma distribution with shape ξ and scale χ; $\mathcal{LN}$ log-normal distribution with "mean" $\tilde{\eta}$ and "variance" $\tilde{\zeta}^2$.

Parameter	Units	$\hat{R}$	Median Value	95% CI	Width Ratio
$\overline{T}$	Sv	1.001	32.6317	[31.2047, 34.0538]	0.13837
α	Sv y^{-1}	1.0007	−0.013584	[−0.054013, 0.0293]	0.085205
r	—	1.0066	0.55246	[0.47413, 0.63057]	0.16441
μ ($\times 10^3$)	m y^{-1}	1.0007	2.6671	[1.1105, 4.2612]	0.028929
ν	m	0.99976	6.9845	[6.9619, 7.0065]	0.018982
ρ	Sv m^{-1}	0.9996	21.3501	[10.4544, 32.4271]	0.029465
π^2 ($\times 10^6$)	(m y^{-1})^2	1.0001	$(1.1673)^2$	$[(0.75971)^2,\ (1.9104)^2]$	0.00056614
σ^2 ($\times 10^6$)	m^2	1.0019	$(26.2588)^2$	$[(24.4292)^2,\ (28.3339)^2]$	0.00024641
δ^2 ($\times 10^6$)	m^2	0.99995	$(8.3539)^2$	$[(7.3177)^2,\ (9.4754)^2]$	0.00037666
τ^2 ($\times 10^6$)	m^2	0.99973	$(66.9832)^2$	$[(54.0808)^2,\ (85.3079)^2]$	0.00040194
γ^2 ($\times 10^6$)	(m y^{-1})^2	0.99995	$(0.6992)^2$	$[(0.40244)^2,\ (1.1171)^2]$	0.00090338
ω^2	Sv^2	0.9997	$(0.708)^2$	$[(0.4832)^2,\ (1.0033)^2]$	0.00058865
ϕ ($\times 10^3$)	km^{-1}	1.0025	0.68742	[0.52277, 0.87158]	0.0040641
λ ($\times 10^3$)	km^{-1}	1.0005	0.8429	[0.43847, 1.6407]	0.80349

Supplementary Table 6. Summary of posterior solutions for scalar parameters. The symbol $\hat{R}$ is a convergence monitor of Gelman and Rubin[25], such that values near 1 indicate convergence. Median Value and 95% credible interval (CI) are computed from the ensemble of posterior model solutions. The Width Ratio is defined as ratio of the width of the posterior 95% CI to the prior 95% CI width.

Parameter	Units	Truth	Median Value	95% CI
$\overline{T}$	Sv	32.8942	32.0523	$[30.9524,\ 33.0873]$
α	Sv y^{-1}	-0.018899	-0.023436	$[-0.059135,\ 0.0090315]$
r	—	0.54595	0.53247	$[0.46355,\ 0.60654]$
μ $(\times 10^3)$	m y^{-1}	2.977	3.1574	$[1.2438,\ 5.1438]$
ν	m	6.9876	6.9947	$[6.9739,\ 7.0165]$
ρ	Sv m^{-1}	23.5497	20.974	$[14.9067,\ 27.6991]$
π^2 $(\times 10^6)$	(m y^{-1})^2	$(1.078)^2$	$(1.4473)^2$	$[(0.94505)^2,\ (2.2444)^2]$
σ^2 $(\times 10^6)$	m^2	$(26.443)^2$	$(25.5557)^2$	$[(23.6732)^2,\ (27.7207)^2]$
δ^2 $(\times 10^6)$	m^2	$(8.7092)^2$	$(9.2437)^2$	$[(8.3297)^2,\ (10.1856)^2]$
τ^2 $(\times 10^6)$	m^2	$(67.1828)^2$	$(66.178)^2$	$[(54.0051)^2,\ (83.3185)^2]$
γ^2 $(\times 10^6)$	(m y^{-1})^2	$(0.64645)^2$	$(0.80521)^2$	$[(0.54918)^2,\ (1.1481)^2]$
ω^2	Sv^2	$(0.77083)^2$	$(0.34671)^2$	$[(0.23695)^2,\ (0.51894)^2]$
ϕ $(\times 10^3)$	km^{-1}	0.63572	0.60636	$[0.46714,\ 0.78344]$
λ $(\times 10^3)$	km^{-1}	0.79168	0.83584	$[0.44534,\ 1.6007]$

Supplementary Table 7. Summary of first synthetic data experiment. Comparison between the true (withheld) parameter values and the posterior model estimates.

Parameter	Units	True Value	Median Value	95% CI
ν	m	6.9876	6.9707	$[6.9506,\ 6.9918]$
δ^2 $(\times 10^6)$	m^2	$(8.7092)^2$	$(7.2674)^2$	$[(6.4296)^2,\ (8.1361)^2]$
τ^2 $(\times 10^6)$	m^2	$(67.1828)^2$	$(62.0712)^2$	$[(50.8668)^2,\ (78.9978)^2]$
γ^2 $(\times 10^6)$	(m y^{-1})^2	$(0.64645)^2$	$(0.80316)^2$	$[(0.55894)^2,\ (1.1291)^2]$

Supplementary Table 8. Summary of second synthetic data experiment. Comparison between the true (withheld) parameter values and the posterior model estimates.

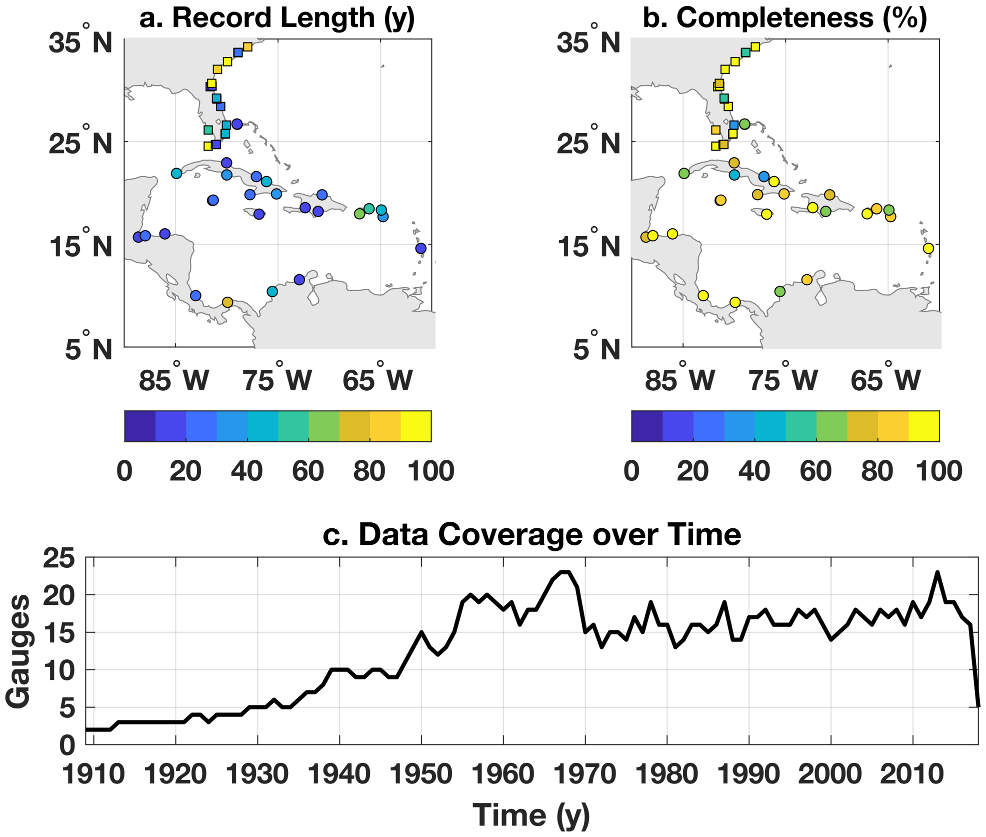

Supplementary Figure 1. Characteristics of tide-gauge data. a, Record length of tide-gauge records (number of y between the first and last measurements made during the study period). Yellower (bluer) colors indicate longer (shorter) records. **b,** Record completeness (percentage of y during the record length for which annual data are available). Yellower (bluer) colors indicate more (less) complete records. **c,** Number of tide gauges returning annual sea-level data during the course of the study period.

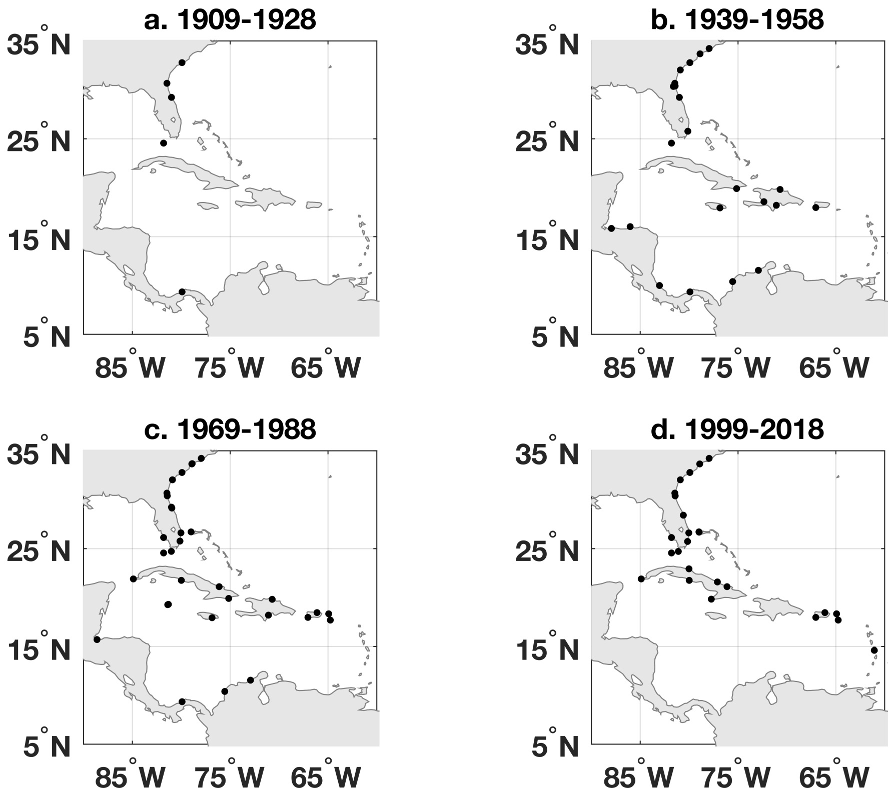

Supplementary Figure 2. Availability of tide-gauge data over time. Dots show tide gauges with at least 1 y of data during **a,** 1909–1928, **b,** 1939–1958, **c,** 1969–1988, and **d,** 1999–1928.

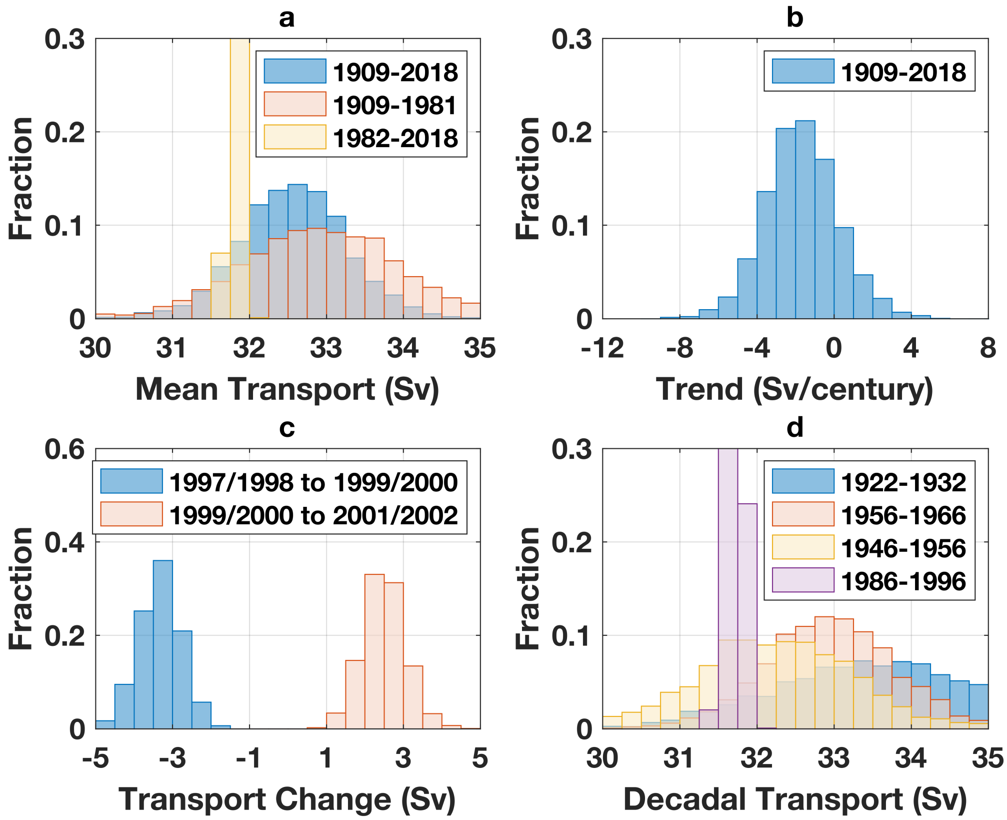

Supplementary Figure 3. Aspects of the posterior solution. a, Blue, orange, and yellow are histograms of transport $\boldsymbol{T}$ averaged during 1909–2018, 1909–1981, and 1982–2018, respectively (Sv). **b,** Histogram of the transport trend $\rho \boldsymbol{b}^{\mathsf{T}} \boldsymbol{\Delta} + \alpha$ over 1909–2018 (Sv century^{-1}). **c,** Blue (orange) is the histogram of the change in transport $\boldsymbol{T}$ between 1997/1998 to 1999/2000 (1999/2000 to 2001/2002) in units of Sv. **d,** Histograms of decadally averaged transport $\boldsymbol{T}$ in units of Sv: blue 1922–1932; orange 1956–1966; yellow 1946–1956; and purple 1986–1996. See Supplementary Table 4 for descriptions of symbols.

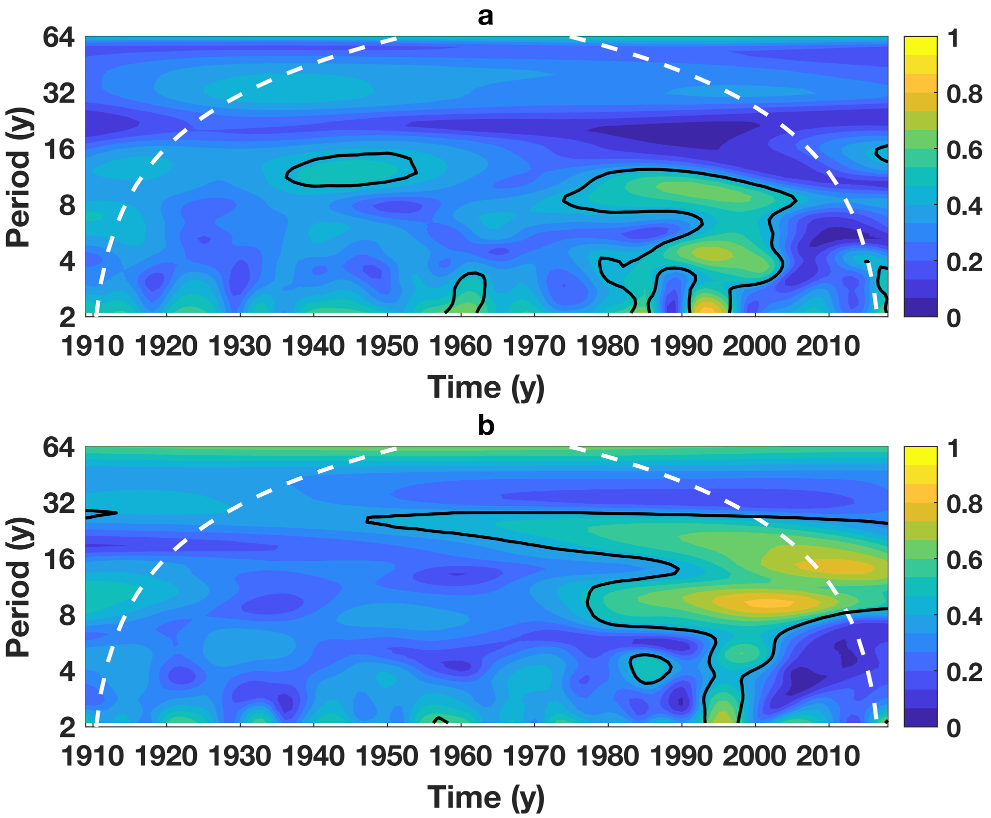

Supplementary Figure 4. Wavelet coherences. Magnitude squared wavelet coherence between Florida Current transport $\boldsymbol{T}$ and **a,** North Atlantic Oscillation and **b,** Atlantic Multidecadal Variability. Values are computed as follows. For each ensemble member, the wavelet coherence is computed between the transport solution and the climate index. For the same ensemble member, two random time series are generated, which have identical Fourier amplitudes to the transport solution and climate index, but randomized phases, and the wavelet coherence between the random time series is computed. Shaded colors represent medians of the set of wavelet-coherence values computed between all transport solutions and the given climate index. Black contouring indicates where 68% of wavelet coherences computed between transport solutions and the climate index exceed the value calculated between the pairs of random time series.

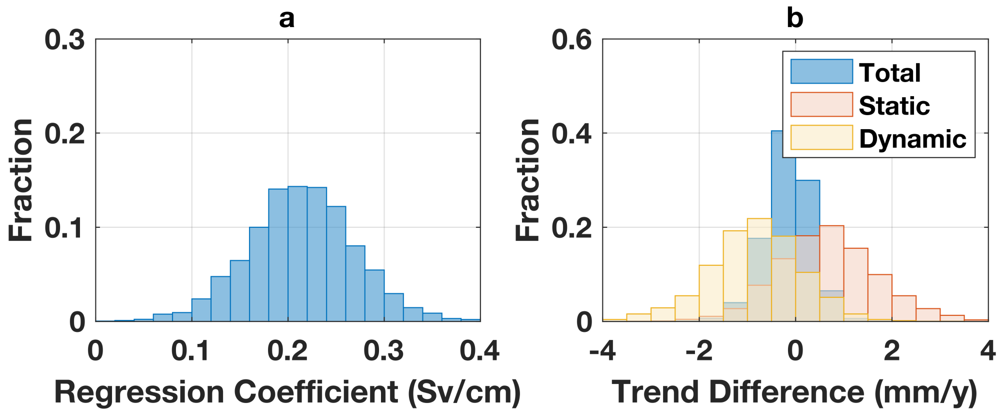

Supplementary Figure 5. More aspects of the posterior solution. a, Histogram of posterior solutions for the regression coefficient ρ (Sv cm^{-1}) between sea-level difference across Florida Straits and Florida Current transport. **b,** Histogram of posterior solutions for the total (blue), static (orange), and dynamic (yellow) trends in sea-level difference across Florida Straits, which are computed respectively as $\boldsymbol{b}^{\mathsf{T}}\boldsymbol{\Delta}$, $-\alpha/\rho$, and $\boldsymbol{b}^{\mathsf{T}}\boldsymbol{\Delta} + \alpha/\rho$ (mm y^{-1}) (see Methods). See Supplementary Table 4 for descriptions of symbols.

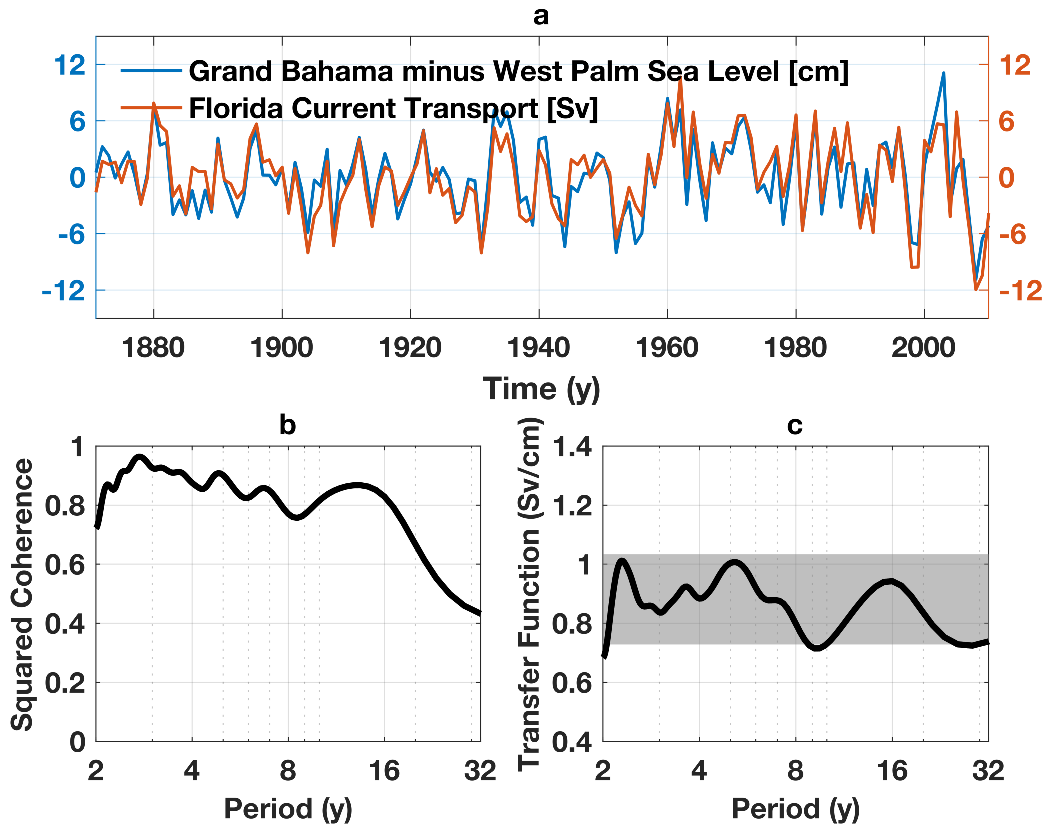

Supplementary Figure 6. Frequency-domain analysis of sea level and transport from SODA. **a,** Blue (orange) is annual sea-level difference across Florida Straits (Florida Current transport) during 1871–2010 from SODA. Both time series have been detrended. **b,** Black line is magnitude-squared coherence between sea-level difference and transport for the first 128 y of the SODA solution (1871–1998). All values are statistically significant at the 95% confidence level based on comparison against synthetic time series. **c,** Black line is amplitude of the transfer function (using sea-level difference as the input and transport as the output). Gray shading is the 95% posterior credible interval on the transfer coefficient ρ from a synthetic data experiment based on SODA (see Methods). Admittance and coherence calculations are based on Welch's method using a window length of 32 and 50% overlap.

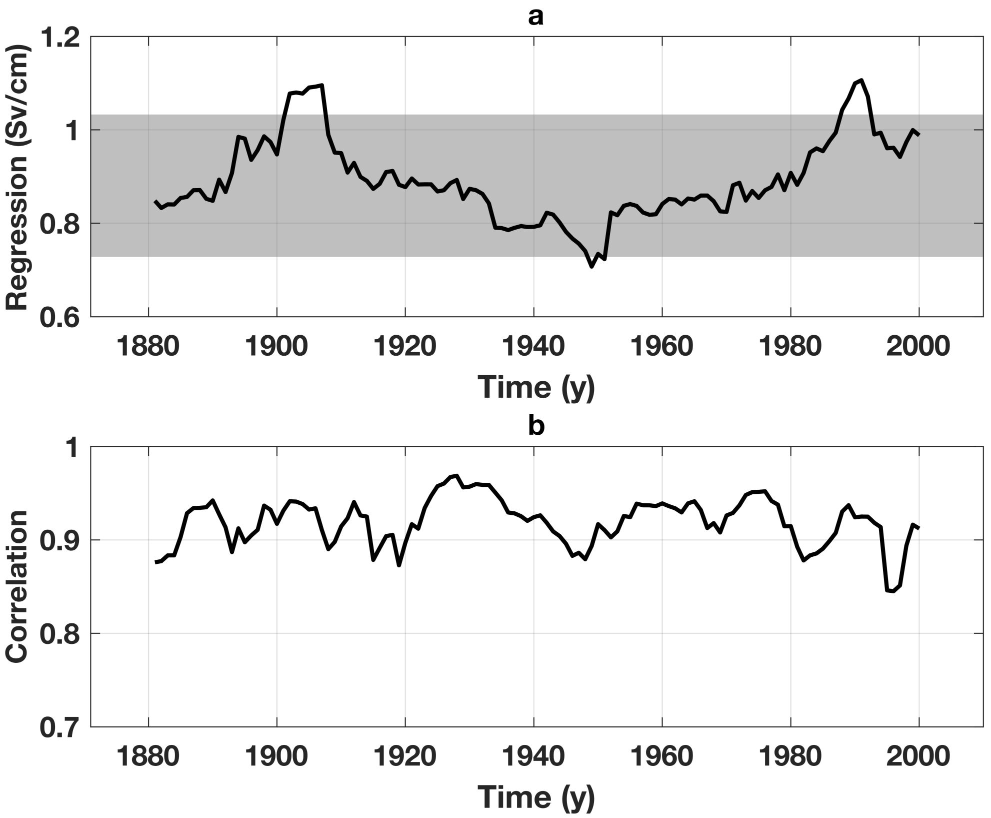

Supplementary Figure 7. Time-domain analysis of sea level and transport from SODA. a, Black line is regression coefficient between annual sea-level difference across Florida Straits and Florida Current transport for sliding 20-y windows during 1871–2010 from SODA. Gray shading is the 95% posterior credible interval on the regression coefficient ρ from a synthetic data experiment based on SODA (see Methods). **b,** Black line is correlation coefficient between annual sea-level difference across Florida Straits and Florida Current transport for sliding 20-y windows during 1871–2010 from SODA.

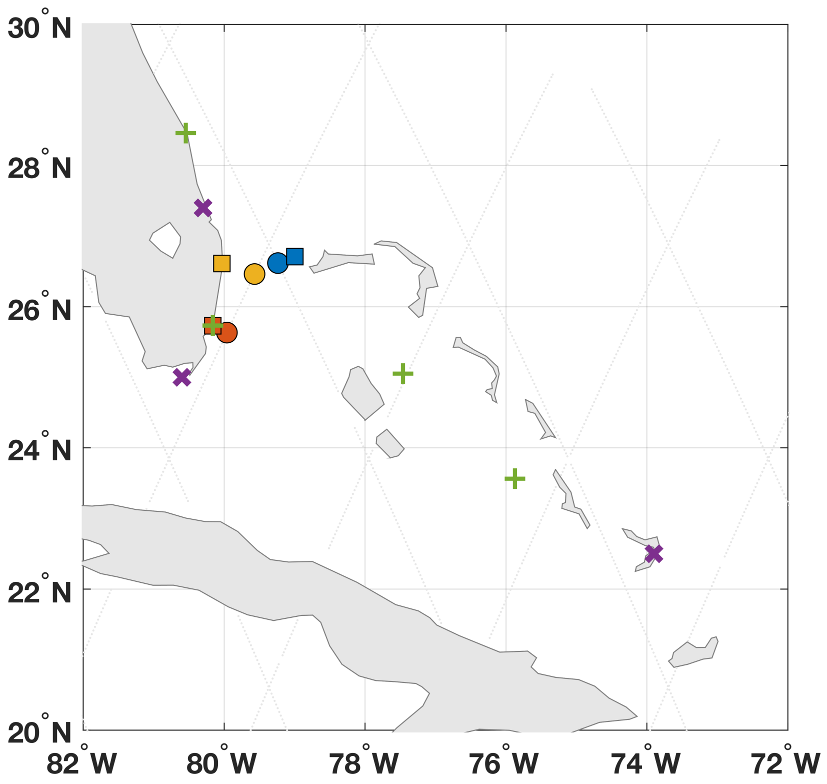

Supplementary Figure 8. Locations of ancillary observational assets. Shaded squares are tide-gauge locations (blue is Settlement Point; orange is Virginia Key; yellow is West Palm Beach). Shaded circles are the along-track satellite-altimeter data points that are nearest the corresponding tide gauge. Light gray criss-crossing marks ascending and descending altimeter tracks. Green + symbols denote locations of GPS stations (cf. Supplementary Table 1). Purple × symbols are the locations of proxy sea-level indicators (cf. Supplementary Table 2).

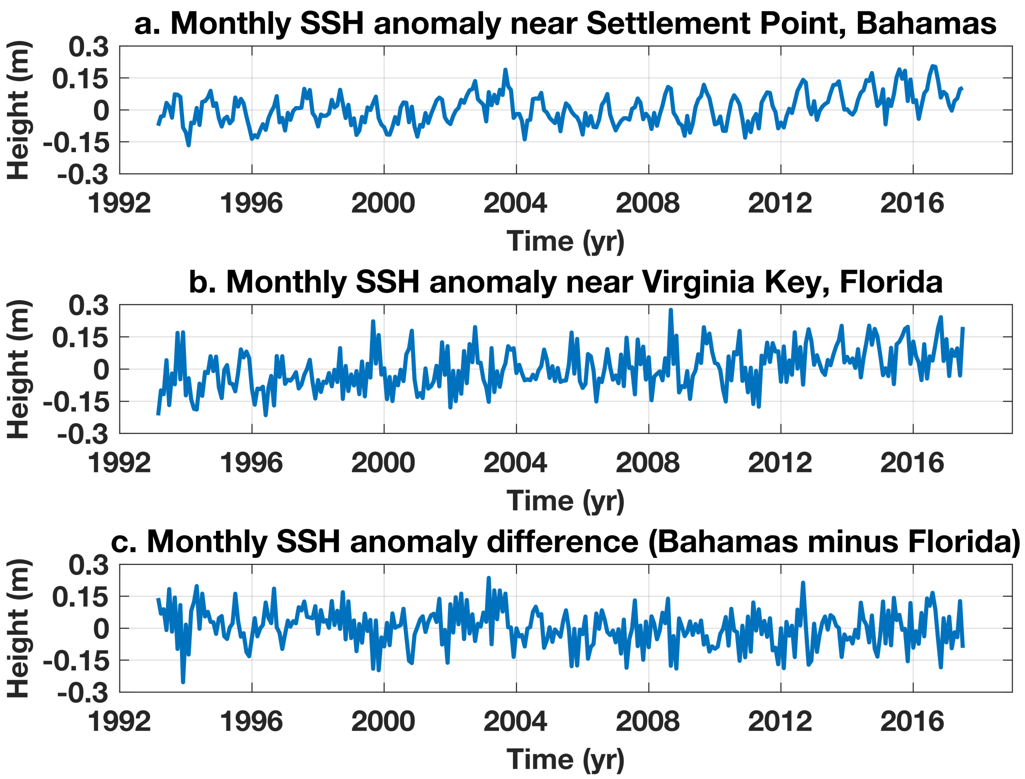

Supplementary Figure 9. Altimetric sea-surface height. Monthly time series of anomalous sea-surface height from satellite altimetry near **a,** Settlement Point, Bahamas, **b,** Virginia Key, Florida, and **c,** the difference between the two time series. Values shown here are calculated by bin averaging the raw 1-Hz data provided by Birol et al.[26] by year and month. See Supplementary Figure 8 for the locations of the time series.

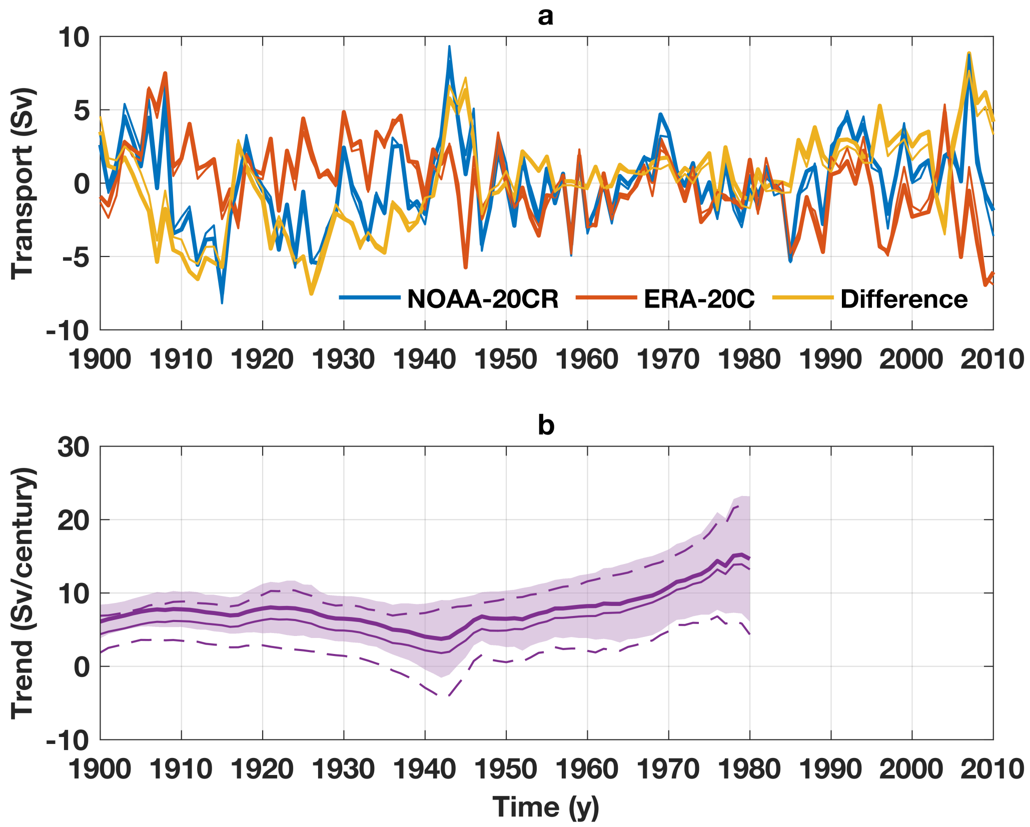

Supplementary Figure 10. Sverdrup and Ekman transport at 27°N from reanalyses. a, Thick blue and orange lines are annual geostrophic Sverdrup transport at 27°N computed from the NOAA-20CR and ERA-20C reanalyses, respectively, while the thick yellow line is the difference between the blue and orange lines. The thin lines are the same as the respective thick lines, only they include the ageostrophic Ekman transport in addition to the geostrophic Sverdrup transport. Time mean values have been removed from all time series. **b,** Thick line and light shading represent the best estimate and 95% confidence interval of the trend in geostrophic Sverdrup transport difference (thick yellow from a) for all periods starting between 1900 and 1980 and all ending in 2010. Thin line and dashed lines are the same, but include the Ekman transport difference in addition to the Sverdrup transport difference (thin yellow from a).

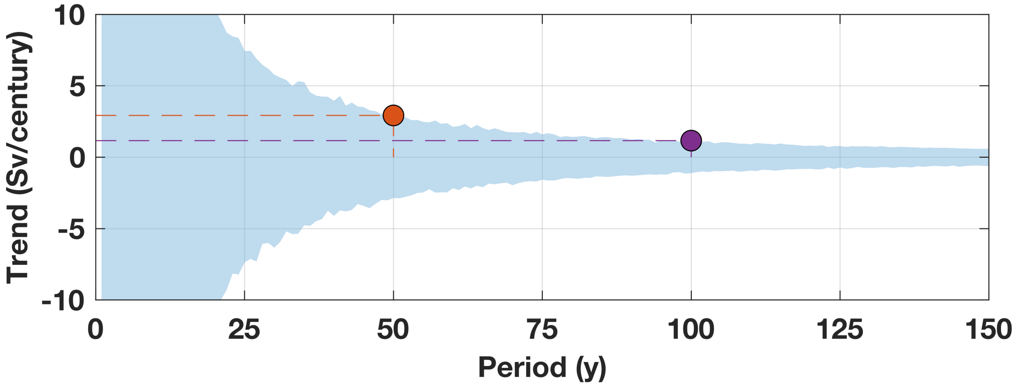

Supplementary Figure 11. Simulated trends in Antilles Current transport. Blue shading bounds the 95% confidence interval on stochastic trends in Antilles Current transport, based on simulations of a stationary red-noise process and an observed integral timescale of 19 days and variance of $(7.5\ \mathrm{Sv})^2$ for the Antilles Current. Orange and purple dots mark the trends for 50- and 100-y periods mentioned in the main text.

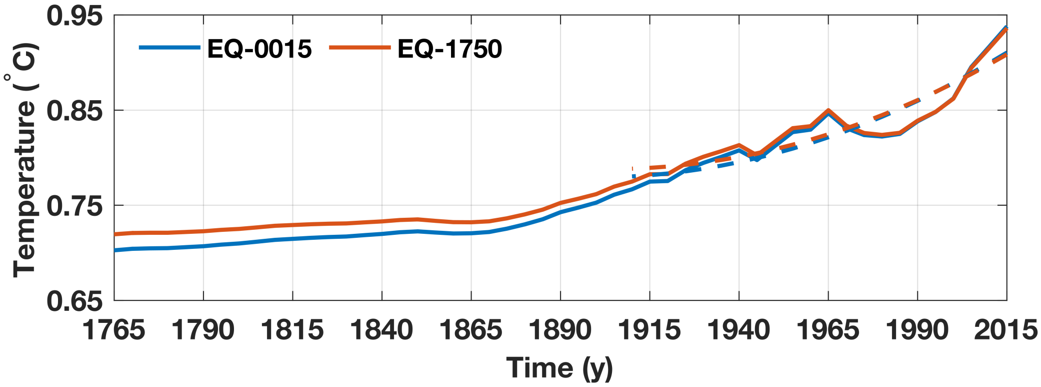

Supplementary Figure 12. Regional ocean warming over the past 250 y. Thick solid blue is the time series of ocean temperature averaged over the full-depth northern North Atlantic and Arctic Oceans, between 27°N and the Bering Strait, from the EQ-0015 empirical ocean circulation model experiment of Gebbie and Huybers[27], where the ocean is in equilibrium with surface conditions in the year 15 CE. Note that the time series of global-ocean mean temperature has been removed. Thick orange line is the same, but from the EQ-1750 experiment, where the ocean is in equilibrium in 1750 CE. Colored dashed lines are second-order polynomials fit to the respective solid lines for the period 1910–2015 CE; both dashed lines indicate an overall regional warming of ~ 0.1°C during that period.

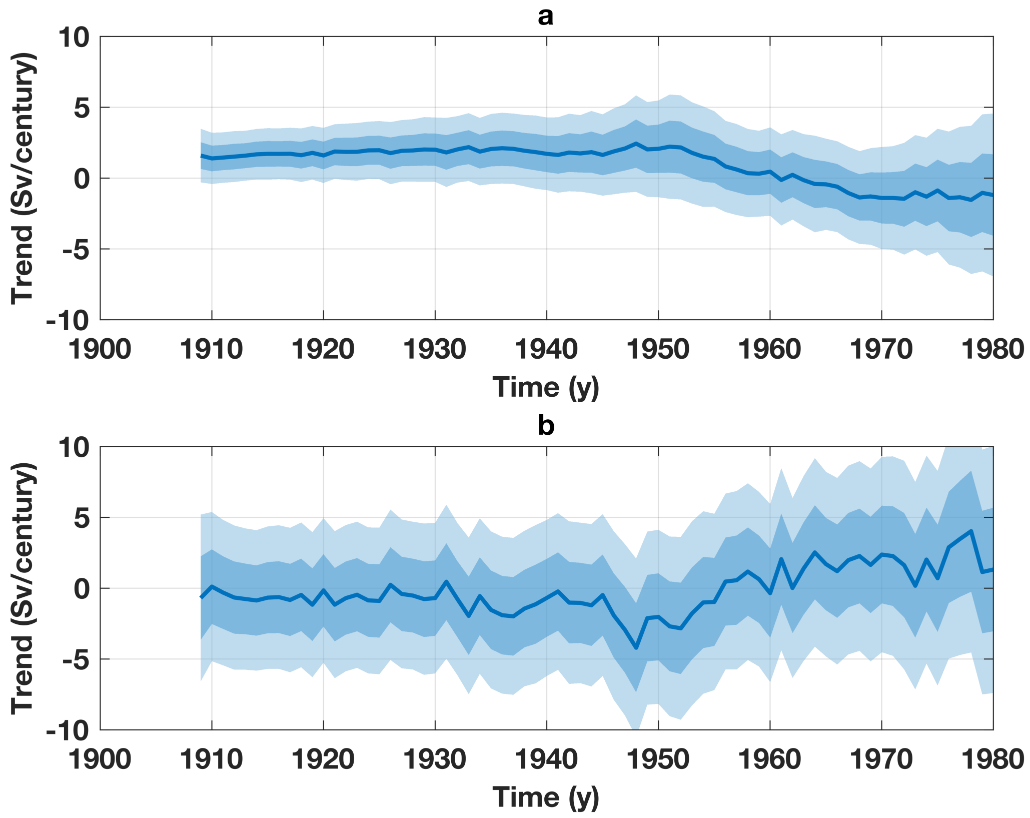

Supplementary Figure 13. Transport trends. a, Estimated trends in transport by the deep return flow of the overturning circulation for all periods starting between 1909 and 1980 and all ending in 2018 (positive northwards). Solid blue line is the best estimate while dark and light shading are respectively the estimated 68% and 95% confidence intervals. See Supplementary Information for more details on the estimates and their caveats. **b,** As in (a) but showing transport trends due to the thermocline recirculation.

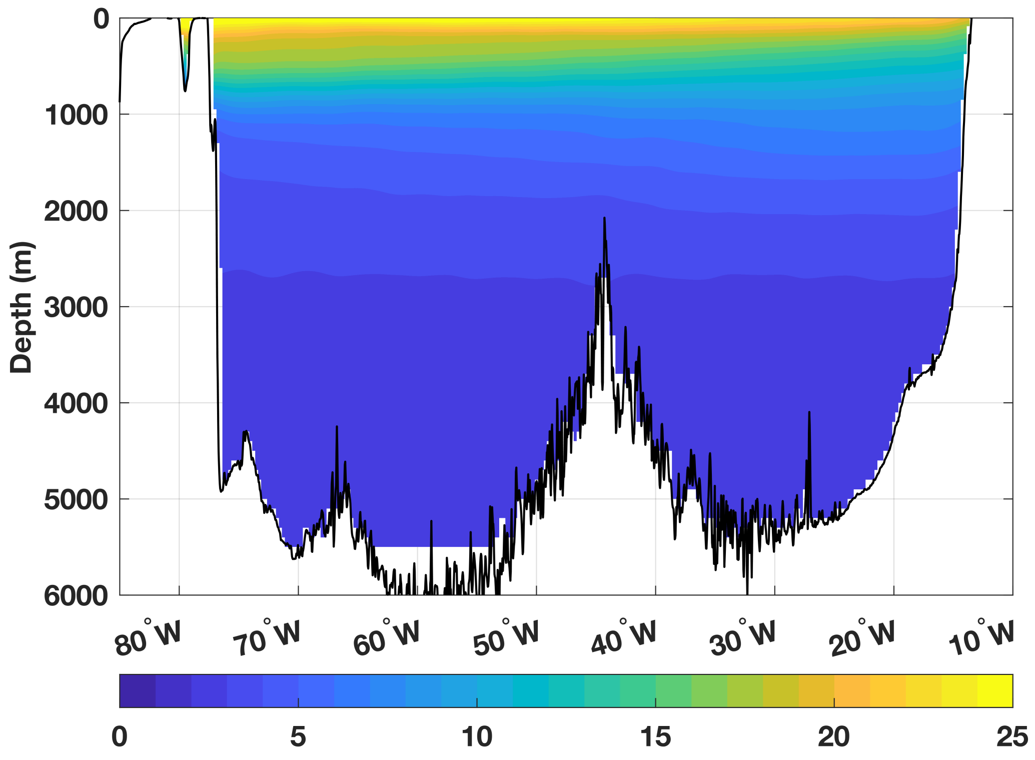

Supplementary Figure 14. Ocean temperatures. Time-mean (climatological) ocean potential temperature along 27°N in the Atlantic from the World Ocean Atlas 2018 as a function of depth and longitude (units °C).

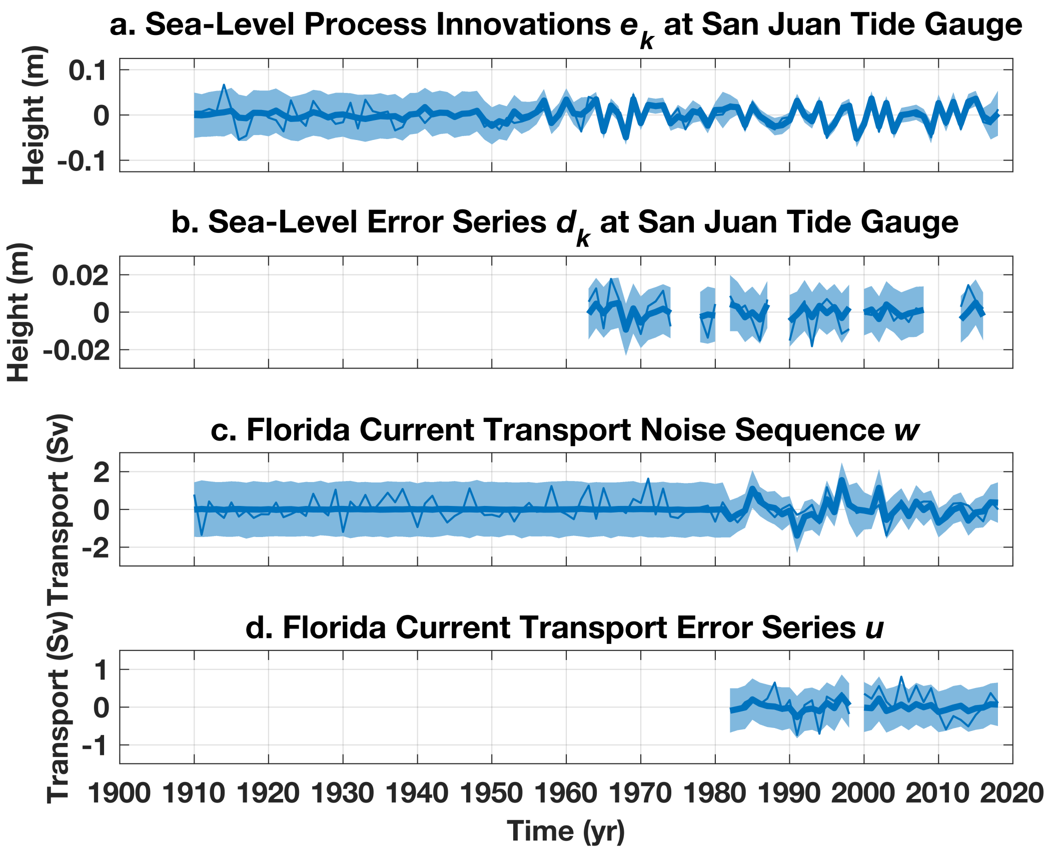

Supplementary Figure 15. Examples of residual time series. Posterior median (solid lines) and pointwise 95% credible intervals (light shading) of the sea-level **a,** process innovations $\boldsymbol{e}_k$ and **b,** data errors $\boldsymbol{d}_k$ at the San Juan (Puerto Rico) tide gauge. Posterior median (solid lines) and pointwise 95% credible intervals (light shading) of the transport **c,** noise sequence w_k and **d,** data errors u_k.

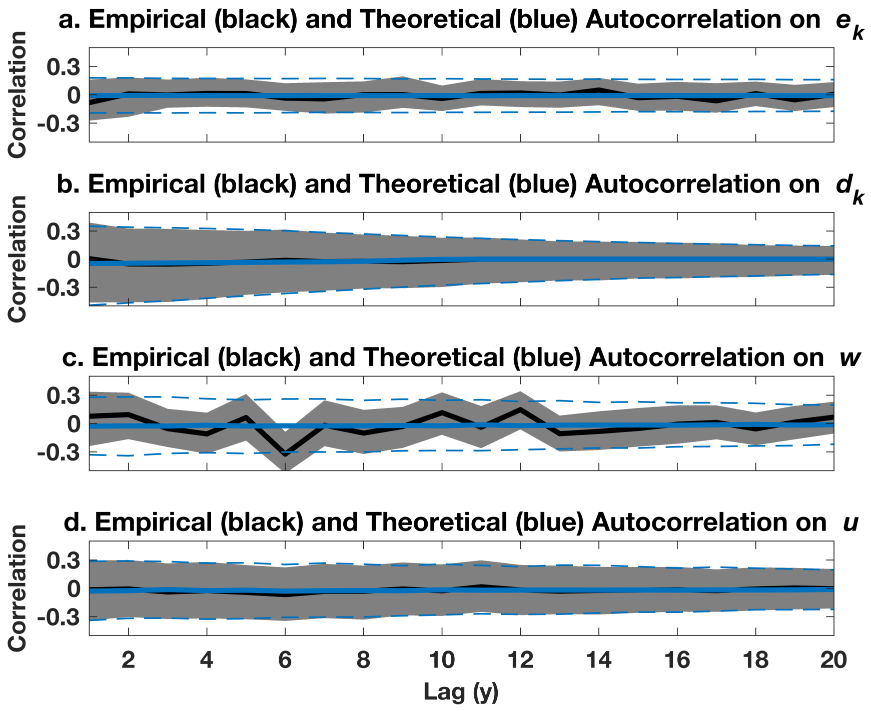

Supplementary Figure 16. Autocorrelation of the residuals. Posterior medians (solid black) and pointwise 95% credible intervals (gray shading) of the sample autocorrelation coefficient computed empirically from posterior solutions for the **a,** sea-level process innovations $\boldsymbol{e}_k$, **b,** sea-level data errors $\boldsymbol{d}_k$, **c,** transport noise sequence w_k, and **d,** transport data errors u_k. Solid and dashed blue lines are the mean $\pm$ twice the standard error on the autocorrelation coefficients expected theoretically from white noise with the same temporal degrees of freedom.

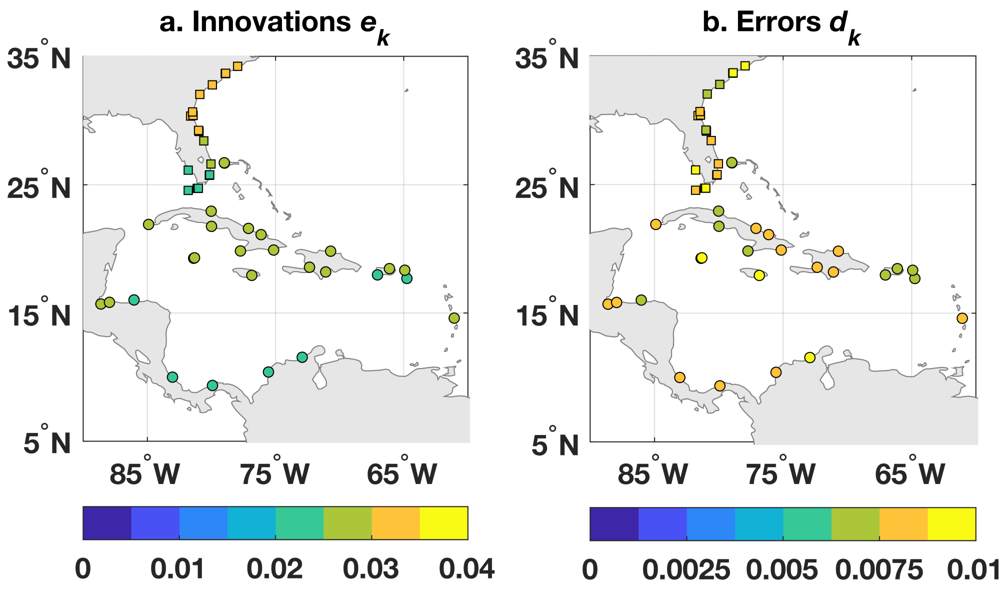

Supplementary Figure 17. Amplitude of sea-level residual time series. Median values of the standard deviation (m) computed from posterior solutions for the sea-level **a,** process innovations $\boldsymbol{e}_k$ and **b,** data errors $\boldsymbol{d}_k$ at all tide-gauge locations.

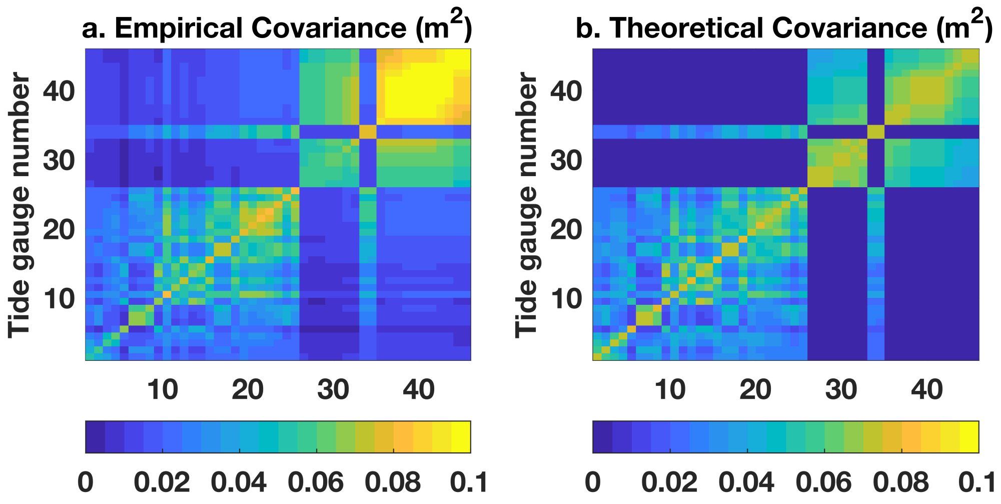

Supplementary Figure 18. Spatial covariance of sea-level process innovations. Covariance (m^2) between all pairs of sea-level process innovations $\boldsymbol{e}_k$ computed **a,** empirically based on posterior solutions for $\boldsymbol{e}_k$ and **b,** theoretically using posterior solutions for σ^2 (Supplementary Table 6) and the assumed covariance structure Eq. (2). The "tide-gauge number" along x- and y-axes refer to the values given in the leftmost column in Supplementary Table 3.

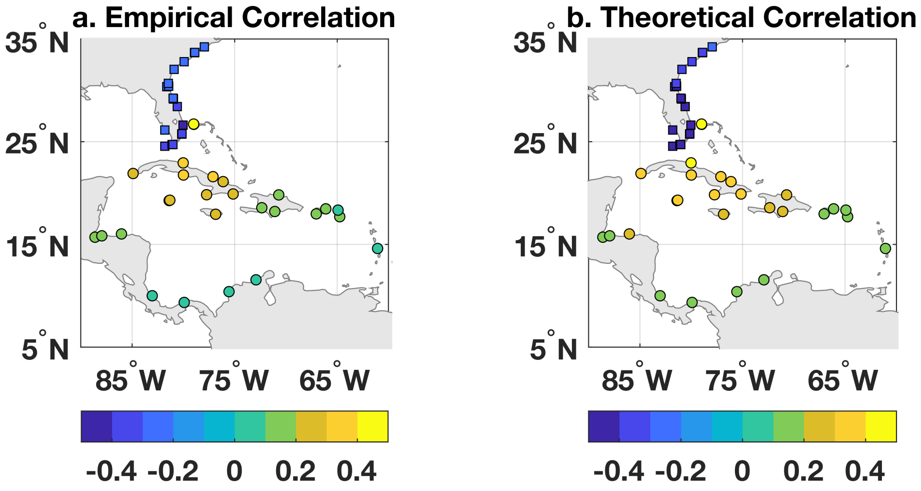

Supplementary Figure 19. Correlation between sea level and Florida Current transport. a, Shading is the median value of the Pearson correlation coefficient computed empirically between posterior Bayesian model solutions for the Florida Current transport and sea level at every location after linear trends are removed. **b,** Shading is the median theoretical value of the Pearson correlation coefficient based on the Bayesian model process-level equations and posterior solutions for the model parameters (Supplementary Table 6).

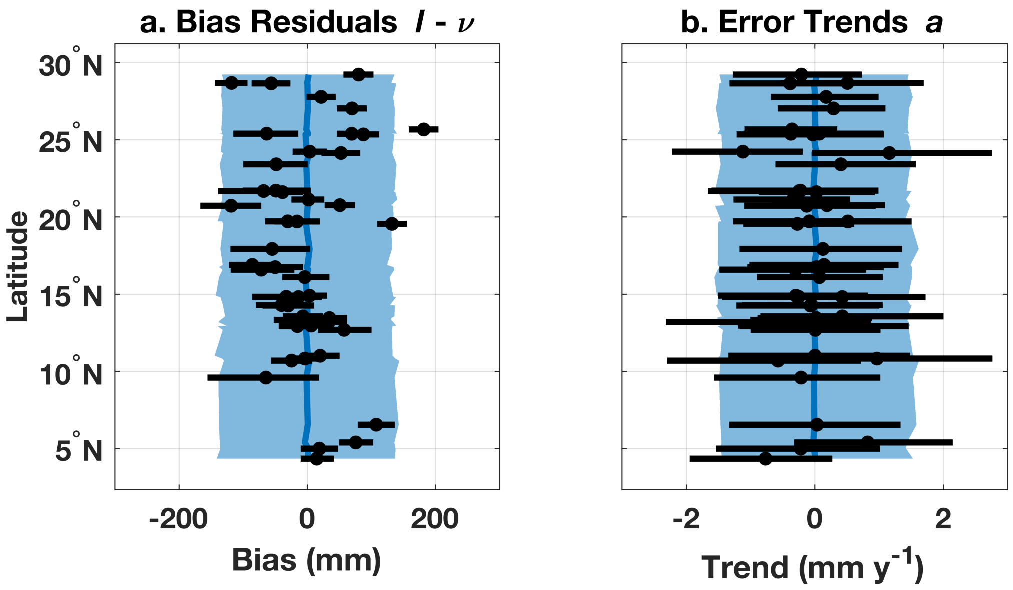

Supplementary Figure 20. Spatial structure of tide-gauge residual vectors. Posterior medians (black dots) and pointwise 95% credible intervals (black lines) for the tide-gauge **a,** data-bias anomalies $\boldsymbol{\ell} - \nu\mathbf{1}$ (m) and **b,** error trends $\boldsymbol{a}$ (mm y^{-1}). Also shown are the means (solid blue) and 95% credible intervals on these fields estimated from their assumed functional forms and posterior solutions for the respective variance parameters τ^2 and γ^2 (Supplementary Table 6).

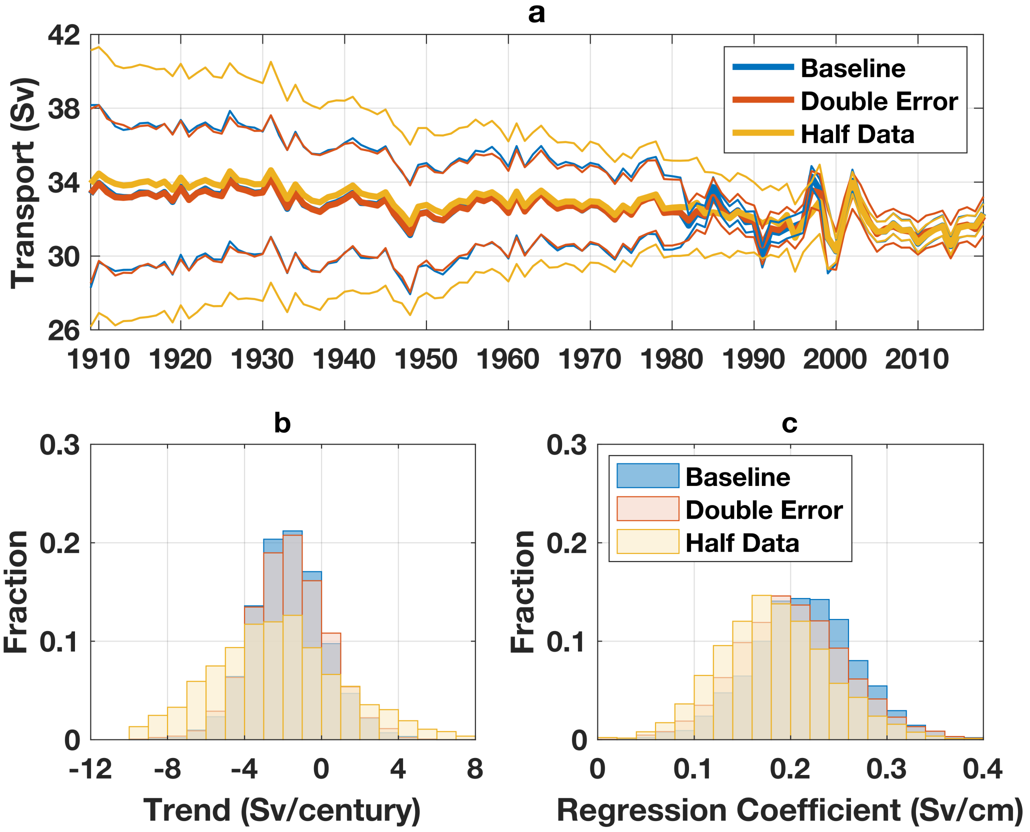

Supplementary Figure 21. Sensitivity of Bayesian model solution to input transport data. Summary of results from sensitivity experiments using different forms of the Florida Cable transport data. **a,** Time series of transport (thick lines are posterior medians; thin lines bound the posterior 95% pointwise credible intervals). **b,** Histograms of the 110-y trend (1909–2018) in Florida Current transport. **c,** Regression coefficient between sea-level difference across Florida Straits and Florida Current transport. Blue values are from the "baseline" model experiment discussed in the main text. Orange values are based on an "double error" experiment wherein the standard errors on the transport data during 1982–2018 are doubled. Yellow values are based on a "half data" experiment where the algorithm is only given the cable data during the period 2000–2018 and the 1982–1998 values are withheld.

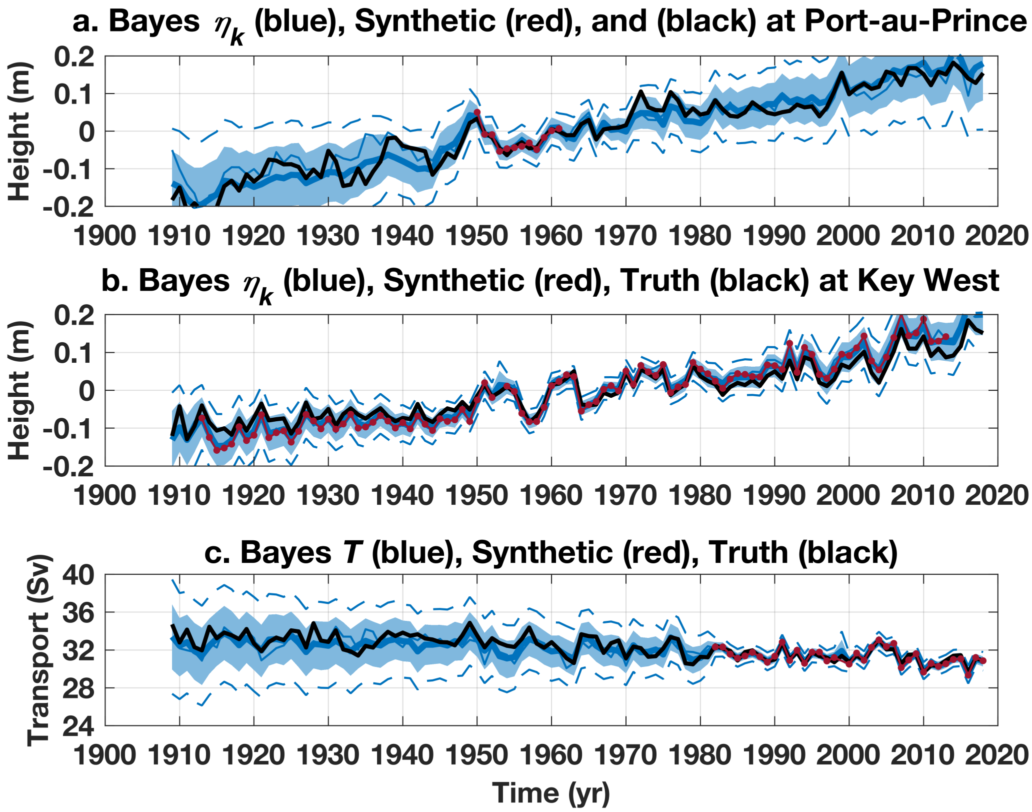

Supplementary Figure 22. Examples of results from first synthetic data experiment. Synthetic observations (red), true values (black), and posterior medians (thick blue), pointwise (blue shading) and pathwise (dashed blue) 95% credible intervals, and an arbitrary ensemble member (thin blue) of **a,** sea level at the Port-au-Prince (Haiti) tide gauge, **a,** sea level at the Key West (USA) tide gauge, and **c,** Florida Current transport.

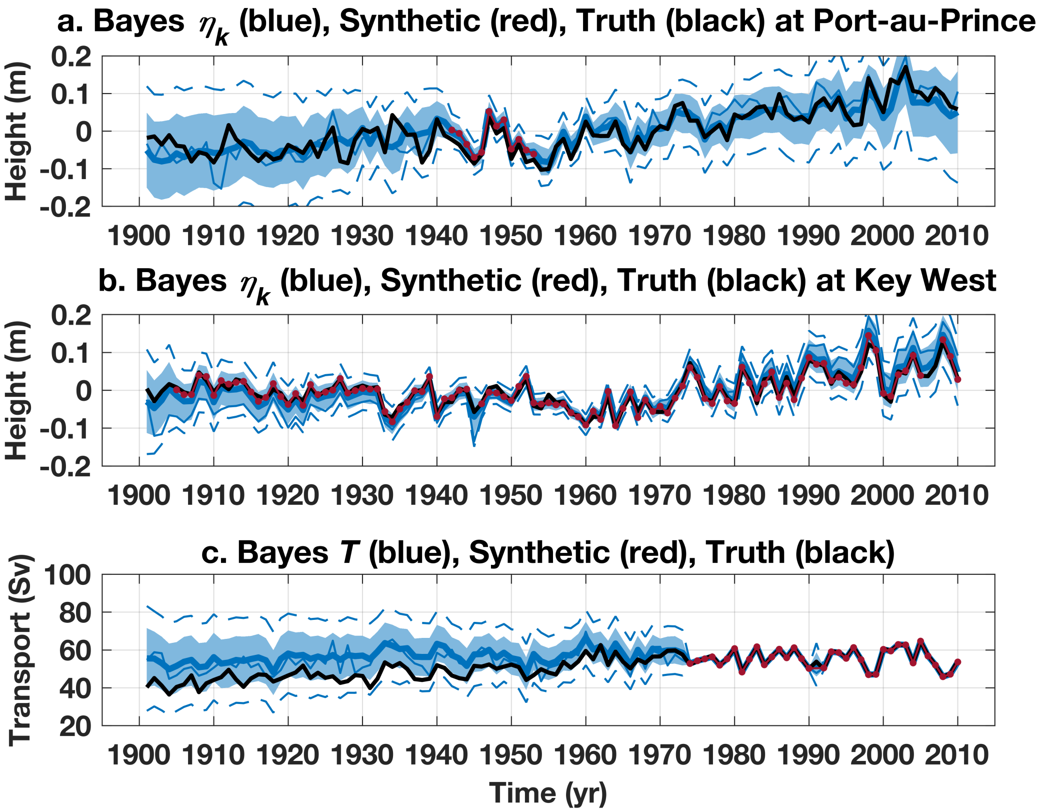

Supplementary Figure 23. Examples of results from second synthetic data experiment. Synthetic observations (red), true values (black), and posterior medians (thick blue), pointwise (blue shading) and pathwise (dashed blue) 95% credible intervals, and an arbitrary ensemble member (thin blue) of **a,** sea level at the Port-au-Prince (Haiti) tide gauge, **a,** sea level at the Key West (USA) tide gauge, and **c,** Florida Current transport.
