# Supplementary material for: Likely weakening of the Florida Current during the past century revealed by sea-level observations
Source: Nat Commun. 2020 Aug 7;11:3973. doi: 10.1038/s41467-020-17761-w (PMC7415143; doi:10.1038/s41467-020-17761-w)
Supplement: Supplementary file 2 — Peer Review File [file 41467_2020_17761_MOESM2_ESM.docx]

Peer Review Information

**Journal:** Nature Communications

**Manuscript title:** Likely weakening of the Florida Current during the past century revealed by sea-level observations

**Corresponding author name(s):** Dr Christopher Piecuch

**Editorial notes:**

**Transferred manuscripts**

This manuscript has been previously reviewed at another journal that is not operating a transparent peer review scheme. This document only contains reviewer comments and rebuttal letters for versions considered at *Nature Communications*.

**Reviewer comments & decisions:**

**Reviewer comments, first version:**

Reviewer #1 (Remarks to the Author):

Piecuch, Nature Communications Paper for Review. Weakening of the Gulf Stream at Florida Straits over the past century inferred from coastal sea-level data

Since my expertise is in the quantitative methods and Bayesian statistical modeling and not in ocean processes, I will concentrate my review on the methods and supplementary information. I have only a few comments to clarify results and outcomes within the main text.

Purpose: This paper seeks to evaluate a potential weakening of the Florida current transport over the past 110 years on decadal and centennial time scales by combining data from direct measurements of transport and tide gauge data, which is a possible proxy for transport. Using a hierarchical Bayesian framework, the paper makes probabilistic statements about the likelihood that the Florida current has slowed and implies that this may have significance pertaining to the hypothesized AMOC slowing, which would have extreme climate consequences. Piecuch looks at several variables in order to make these assessments. He models coastal sea level with temporally linear trend, which is correlated in space, through a covariance function based on distance in space between two sites. He assumes a balance between coastal sea level and current transport in order to infer the change in transport per unit change in sea level over time. Many assumptions are made within this model. However, each is justified or through simulations, climate model output, residual analysis, and sensitivity tests and synthetic data experiments.

This is an interesting, useful, well written, and careful manuscript. It seems ready for publication after a few issues/clarifications, detailed below, have been addressed in a responsive revision.

General comments:

My largest issue with the results is the calculation of the main results of the manuscript. Please clarify why you chose to compare the entire 11- year reconstruction to the observational record (Figure 2 and line 81) for the main conclusion that recent mean transport is slower than previous. The 95% CI is smaller for the 110-year average than it would be for the reconstruction prior to 1982 because of the inclusion of the observational cable data. What would this probability be if the observational record were excluded? Is this standard practice to compare a full model result to part of the model as opposed to separating the two results and comparing?
You may need to be more specific about words like occasionally, short, near-surface, unless those are generally accepted in this subject area (e.g., 2nd paragraph of main text).


Main conclusions and results that need to be clearer:
- Line 45: Does “balanced” mean that one is necessary for the other to occur or that one is causal of the other? Does this just mean that there is a correlation? Please clarify.
- Line 55: Define or give example of coastally trapped signals.
- Line 86: what does “strong observational constraints on the process” mean? That we have good evidence that these represent this process?
- Line 90: What is the significance of this standard deviation? And is that the standard deviation over all times evaluated? Why use the standard deviation here instead of the95% CI?
- Line 91: Clarify why it is significant to compare these two periods, when there is a gap in the record. Could that gap influence that finding? Could one of the measurements be biased and the weakening then strengthening balance out? Why is this a significant result?
- Line 93: Decadal-average transport was likely (P ≥ 0.79) greater than the longterm average during 1922–1932 (33.6 ± 2.8 Sv) and 1956–1966 (33.0 ± 1.7 Sv), but less than average in 1946–1956 (32.2 ± 2.0 Sv) and 1986–1996 (31.7 ± 0.2 Sv) (Supplementary Figure 1d).
o I think you are trying to say that the 1922 and 1956 averages were higher than long term and the 1946 and 1986 were lower than long term, but this is unclear in this sentence.
- 106: What kind of changes? Are we just looking for changes or specifically weakening
- Line 107: Clarify that the trend is likely a negative trend or that the transport likely declined during this period. Otherwise, it is unclear what it declined from.
- Line 121: Clarify what “would have been” means? Is there better wording that could be used here?
- Line 124: Clarify that the regression coefficient signifies the estimated transport.
- Line 136: “more exponentially” or “takes a more exponential form”?
- Line 142: statistical coherence and admittance or coherence and admittance statistics.
- Line 160: Why this interpretation? Are there other interpretations that might be valid?
- Line 170: Rate of rise? Be careful with consistency of terms. In line 167 you refer to this as rise and it could be unclear that you are referring to the same thing here.
- Line 175 – 178: Why not use the estimates from the model referenced in line 174?
- Line 182: “furnished by”, word choice
- Line 194 - 195: Explain/clarify the significance and meaning.
- Line 209: Clarify: Is weaker northward equivalent to stronger southward or are you trying to say that there are different directions in the trends?
- Line 211: Clarify what adjusted for residual autocorrelations means.
- Figure 1: It is difficult to differentiate between the purple and red. Possibly choose a different color for one or the other. A bit hard to read “Northwest Providence” and “Yucatan” in black.
- Figure 4: What type of interpolation. Is the standard deviation calculated from all models (from the average of the models)?
Assumptions that I don’t have the expert knowledge to assess concern:
1. Geostrophic balance
2. Other reasons that tide gauges need to be used cautiously, because they represent different signals (non-oceanographic and geostrophic impacts on sea level and transport). What are these and how are they accounted for?
3. Data assimilation for other tide gauge data for RSL signals.
Detailed comments/questions, line-by-line in the methods and supplementary:

Methods:
- Might there be a negative correlation in sea-surface height between the two regions in equation 3? Did you examine this possibility?
- Line 459: Define X.
- In line 489, you suggest that the longterm sea-level change are unrelated to ocean dynamics. However, the dynamic component of the difference in sea-level trends includes a term that is the opposite of the dynamic component (alpha/rho). Please explain.
- Line 494: Uncelar referential “this interpretation.” Does the interpretation refer to the relationship between dynamic and static terms?
- Line 500: You need to define N, Mk and M. I believe you mean that Mk is the locations where there are tide gauges at time step k, M is the total locations of any tide gauges, and N is the total locations for any measurement or any prediction?
- Line 508: a and l are always selected jointly. Can the selection matrices H and F have both terms for a single tide gauge? Please clarify.
- Line 505: My understanding is that M is the number of tide gauge locations and N is the total number of locations (including the tide gauge locations). Why would the coastal sea level term selected by H ever not be selected? Please clarify?
- Line 612: Does arbitrary mean randomly selected?

Supplementary:

- Table S5: Did you set bounds on the hyperparameters? Clarify why the shape parameters of all of the inverse gamma distributions are the same.
- In some places you specify solutions in cm, some in km, and some in mm.
- You don’t seem to reference some of the supplementary tables and figures within the supplementary text or the main text. Check to make sure you cover each of those.
- Line 14: “I formulate a simple ocean heat budget.”
o “volume is taken to be the full-depth”: Does that mean your calculation of volume includes the full depth? A depth is not a volume. Also, the Bering Strait is not part of the Atlantic Ocean. Are you developing a heat budget for the global oceans or just the Atlantic?
- Line 18: weakening of both the Florida Current AND the deep overturning circulation?
o It is a balance between those two and turbulent ocean heat gain? Just need to clarify this sentence
- Line 25: word choice, throughout? In?
- S6: Clarify what you are doing here, please. These do not seem like equivalent equations.
- Use the same wording to define Q_surf and Q_moc as you use in lines 18-20 (or in lines 18-20 note that these two variables are Q_surf and Q_moc.
- Line 40: maybe use unstippled instead of unhatched because of your wording in the figure caption?
- Line 42: are these ranges or differences? Make sure that is clear and why you are using them.
- Line 48: you can call this Figure S1(b), since that is what it is called in the figure. (same in line 81)
- Line 126: I thought I would see the basis for these estimates in the supplementary
- Line 144: Doesn’t this prove that having less data creates larger posterior solution uncertainty? And the model solutions are in fact sensitive to this choice?
- S13: It is difficult to see the baseline in each of these panels. Could you make the half data and double error transparent to reveal the baseline better?
- Line 149: These are the medians and the 95% CIs, right?
- Line 162: I assume you randomly chose the data to withhold, so this would be true with any data that was removed?
- Line 174: Are you choosing the median posterior model solution or a selection within some CI of solutions? Shouldn’t you assume that the uncertainty in the posterior matters for the generation of the true values or is there no uncertainty in the processes? Not just the data-level equations to perturb the process?
- Figure S14: Is the Bayes Sea-level solution the original solution, from which you generated the synthetic process and synthetic data? Or is it the posterior solution after running the model on the synthetic data? I am assuming the latter, but I would also like to see the former.

Erica L. Ashe


Reviewer #2 (Remarks to the Author):

Review of Weakening of the Gulf Stream at Florida Straits over the past century inferred from coastal sea-level data, by Christopher G. Piecuch

This paper provides a thorough discussion of long-term changes in Florida Current transport over the past 110 years, and finds a weakening of this current over this period. This is a very interesting finding as the Florida Current is a key component of Atlantic Ocean circulation, linked to both the wind-driven circulation and the thermohaline overturning circulation.

Let me first state that I approach this review with my general expertise on the AMOC, but I do not have experience with the Bayesian model used by the author. I therefore recommend that someone with expertise in this methodology reviews this paper as well, if this hasn’t happened already.

The discussion of the causes of the declining trend is very interesting, where the key question is whether the decline is linked to wind changes or to thermohaline overturning changes. Unfortunately, reanalysis data are inconsistent and thus do not allow to determine even the sign of the wind contribution. On the other hand, the paper provides a plausible estimate of the overturning contribution, arguing that the data are consistent with this largely explaining the declining trend of the Florida Current.

I think this is an important study which I recommend for publication in Nature Communications after minor revisions.

Primarily, I would suggest to the author to strengthen the section “Relation to the Deep Overturning” by adding a discussion not only in terms of heat budget consistency but also regarding consistency with transport changes in Sv estimated in previous studies. Given a total Florida Current transport of 32 Sv and a gyre estimate of 23 Sv, this would leave only 9 Sv for overturning, which is less than the total overturning (estimated to be more like 15-20 Sv). Presumably the missing part is flowing northward east of Grand Bahama and thus not part of the Florida Current. What does this mean for the estimate in trend – how much of an AMOC weakening trend would show up in the Florida Current, and how much to the east of Grand Bahama? This issue needs a bit of discussion (even if it can’t be resolved).

Small comments:

The author could also cite some further proxy data studies indicating a long-term overturning decline, e.g. Thibodeau et al. 2018, Zanna et al. 2019.

Line 45: “deep overturning circulation” usually denotes the full “overturning”, i.e. both upper northward and deep southward branch. I suggest to replace with “deep branch of the overturning circulation”.

Line 82: Is it statistically or conceptionally better to compare the period after 1982 to the mean of the entire record, or to the mean of the time preceding 1982? I find it more intuitive to compare two non-overlapping periods, e.g. because the difference then corresponds to how much the current has weakened over time. I also find the wording “that the cable data probably represent the Florida Current in a reduced state of transport” strangely vague. Does it mean that according to the cable data, the Florida Current has probably been weaker since 1982 than before? If so, say so. If not, why not?

Line 103: AMV/AMO: The reality of this variability as a distinct mode is highly questionable, see e.g.
Mann, M. E., Steinman, B. A. & Miller, S. K. Absence of internal multidecadal and interdecadal oscillations in climate model simulations. Nat Commun 11, 49 (2020)
which states: “Here we use a combination of observational data and state-of-the-art forced and control climate model simulations to demonstrate the absence of consistent evidence for decadal or longer-term internal oscillatory signals that are distinguishable from climatic noise.”


Reviewer #3 (Remarks to the Author):

This paper tests a hypothesis that the flow through the Florida Strait has weakened during the last century. This is a topical and important question. Several studies (cited in the manuscript), using proxy data, have concluded that the Atlantic Meridional Overturning Circulation (AMOC) has weakened but there is as yet no clear consensus. Thus, there will be much interest in this paper amongst those who study Atlantic circulation and its impact on climate. The probabilistic method employed seems to be a good choice to extract information from the many incomplete time series and overall the paper is well written. I recommend that the paper be published, but have a few questions that could perhaps lead to some improvements in the paper.

1. I found it curious that the results highlight that the AMOC has declined since the 1970’s. The 1970’s and 1980’s was a time when the North Atlantic was relatively cool compared to the following decades, and the AMV index had low values. Figure 6 and the associated discussion suggest the century long cooling trend in the sub polar gyre and the reduction of the Florida Current are consistent. But are the decadal variations of these quantities also consistent?

2. I wonder if it would be better to merge the two sections “Relation to Wind Stress and the Interior Gyre” and “Relation to the Deep Overturning”? These are distinct from the rest of the paper and explore the implications of the likely slowdown in the Florida Current. It is stated that “weakening of the Florida Current transport must have been balanced by changes in the interior gyre or overturning transports at 27N”. However, there is a third possibility, alluded to in the conclusions, that the reduction is balanced by an increase in transport in the Antilles Current so that the total western boundary current is unchanged. I think it would be better to mention this explicitly at the start of this section.

3. I confess that I am not sufficiently familiar with the statistical technique used to give an expert opinion the details of the analysis. However, the methodology is mostly well described, though I had to search for a definition of the term “innovation sequence”, and it would be better to define that in the text.

4. I would like to see a spatial map of the expected sea-level correlation with transport anomaly that is deduced from the analysis. Is it possible to test with the data whether this relationship between sea-level and transport is stationary in time?

5. Figure S7 gives an overview of the amount of data available and all the details are in the table S3, but I think it would be instructive to add to Figure S7 maps of the data available for some particular decades, e.g. 1920s, 1960s and 2000s.

6. The author is careful to highlight the uncertainties and to quantify the likelihood that the Florida Current has reduced. Although there is significant uncertainty about some results, they are still interesting and valuable, but I wonder if the title might be better as “Likely weakening……. ”

**Author rebuttal, first version:**

**Reviewer 1**—

Piecuch, Nature Communications Paper for Review. Weakening of the Gulf Stream at Florida Straits over the past century inferred from coastal sea-level data.

Since my expertise is in the quantitative methods and Bayesian statistical mod­eling and not in ocean processes, I will concentrate my review on the methods and supplementary information. I have only a few comments to clarify results and outcomes within the main text.

Purpose: This paper seeks to evaluate a potential weakening of the Florida cur­rent transport over the past 110 years on decadal and centennial time scales by combining data from direct measurements of transport and tide gauge data, which is a possible proxy for transport. Using a hierarchical Bayesian frame­work, the paper makes probabilistic statements about the likelihood that the Florida current has slowed and implies that this may have significance pertaining to the hypothesized AMOC slowing, which would have extreme climate conse­quences. Piecuch looks at several variables in order to make these assessments. He models coastal sea level with temporally linear trend, which is correlated in space, through a covariance function based on distance in space between two sites. He assumes a balance between coastal sea level and current transport in order to infer the change in transport per unit change in sea level over time. Many assumptions are made within this model. However, each is justified or through simulations, climate model output, residual analysis, and sensitivity tests and synthetic data experiments.

This is an interesting, useful, well written, and careful manuscript. It seems ready for publication after a few issues/clarifications, detailed below, have been addressed in a responsive revision.

I appreciate the reviewer’s thorough and constructive comments. Below I give point-by-point responses, highlighting where changes were made in the revised manuscript to address these comments.

General comments:

**R1.CA** My largest issue with the results is the calculation of the main results of the manuscript. Please clarify why you chose to compare the entire 110-year reconstruction to the observational record (Figure 2 and line 81) for the main conclusion that recent mean transport is slower than previous. The 95% CI is smaller for the 110-year average than it would be for the reconstruction prior to 1982 because of the inclusion of the observational cable data. What would this probability be if the observational record were excluded? Is this standard practice to compare a full model result to part of the model as opposed to separating the two results and comparing? You may need to be more specific

2

about words like occasionally, short, near-surface, unless those are generally accepted in this subject area (e.g., 2nd paragraph of main text).

Thanks for these questions. Reviewer 2 raises similar issues in comment R2.CD below. My original intent was to highlight a facet of the longterm weakening in transport, namely that the average transport since 1982, when we have direct observations, is lower than the longterm 1909–2018 average. I now realize, from this comment and comment R2.CD, that this was a confusing way to present the results. Therefore, in the revised text, I now compare solutions for transport during the more recent 1982–2018 period to the earlier, non-overlapping period 1909–1981 (11?. 11–12, 90–93, 330–333), as suggested by both Reviewers 1 and 2.

Words like “occasionally,” “short,” and “near-surface” suffice for general pur­poses of this introductory paragraph. Providing more specific details wouldn’t improve the reader’s experience or understanding of the main results. I’ve tried to be comprehensive in terms of referencing, so the interested reader can consult the original papers in case of detailed interest. Therefore, I haven’t adjusted the text on this point.

Main conclusions and results that need to be clearer:

R1.CB Line 45: Does “balanced” mean that one is necessary for the other to occur or that one is causal of the other? Does this just mean that there is a correlation? Please clarify.

I should have been clearer in the original submission. I mean “balanced” in the sense of mass balance. On these timescales, there is no net mass transport across 27^°^N in the Atlantic (Kanzow et al., 2007). Therefore, if there is a change in Florida Current transport, then there must also be compensating or balancing transport changes by other circulations at 27^°^N that together cancel out to give no net transport. The text has been clarified on this point (``. 44–46, 232–237).

R1.CC Line 55: Define or give example of coastally trapped signals.

I now clarify that I mean “remotely driven coastal-trapped waves and currents.” This sentence has been expanded to be more descriptive on these points (``. 59– 63).

R1.CD Line 86: what does “strong observational constraints on the process” mean? That we have good evidence that these represent this process?

I realize that my original wording was unclear. In the revision, I clarify that, because the observations have relatively small errors, the cable data, “strongly constrain the posterior solutions of the transport process” during 1982–2018 (ti. 94–95).

3

R1.CE Line 90: What is the significance of this standard deviation? And is that the standard deviation over all times evaluated? Why use the standard deviation here instead of the 95% CI?

This section describes short-term (interannual and decadal) variability in trans­port. It is meant as context for later discussions of long-term (multidecadal and centennial) changes. I quote the median posterior solution for the standard de­viation over all timescales to give an overall sense of the magnitude of transport variability. This sense wouldn’t be communicated by the 95% credible interval on transport, suggested by the reviewer, since that would contain information about both the variability and the mean. Since my meaning in this sentence is clarified by the sentence that precedes it, no changes to the manuscript were necessary on this point.

R1.CF Line 91: Clarify why it is significant to compare these two periods, when there is a gap in the record. Could that gap influence that finding? Could one of the measurements be biased and the weakening then strengthening balance out? Why is this a significant result?

Good questions. This period is of interest because (1.) there was an outage in the cable data, and (2.) observations of the Yucat´an Current transport upstream in Yucat´an Channel were anomalously low (23.8±1 Sv; Sheinbaum et al., 2002). As a result, the flows through Florida Straits and the Northwest Providence and Old Bahamas (Nicholas and Santaren) Channels during this period have been uncertain and the subject of debate (e.g., Beal et al., 2008; Meinen et al., 2010). My estimate of the Florida Current transport during this period therefore puts a valuable constraint on the flows through the other channels. Thus, this result is interesting. However, it is admittedly something of an aside. I judge that the original text struck a good balance between reporting the interesting result, but not belaboring the point, so I haven’t altered the text in the revision.

The gap in cable data does influence the posterior inference. For example, errors on the estimated transport during 1999 are noticeably larger than during other years between 1982 and 2018 (Figure 2). However, it is unlikely that the gap introduces a bias, such as the reviewer asks after. The cable transport estimates are calibrated against accurate, independent measurements made during regular (roughly bi-monthly) cruises across Florida Straits (e.g., Figure 5), which guard against any longterm bias (e.g., Meinen et al., 2010; Garcia et al., 2014). These technical details are now mentioned in the revised methods section (``. 697– 700).

R1.CG Line 93: Decadal-average transport was likely (P ≥ 0.79) greater than the longterm average during 1922–1932 (33.6± 2.8 Sv) and 1956–1966 (33.0± 1.7 Sv), but less than average in 1946–1956 (32.2±2.0 Sv) and 1986–1996 (31.7±0.2 Sv) (Supplementary Figure 1d). I think you are trying to say that the 1922 and 1956 averages were higher than long term and the 1946 and 1986 were lower than long term, but this is unclear in this sentence.

4

Good suggestion. My original wording was somewhat tortured and circuitous. The reviewer correctly understands my intended meaning. I have more or less adopted the reviewer’s suggested rewording here (``. 103–107).

R1.CH Line 106: What kind of changes? Are we just looking for changes or specifically weakening

This sentence has been adjusted to better communicate the result that the only significant changes on long, multidecadal and centennial timescales correspond to weakening of transport (``. 118; cf. Figure 3).

R1.CI Line 107: Clarify that the trend is likely a negative trend or that the transport likely declined during this period. Otherwise, it is unclear what it declined from.

Thanks for this suggestion. I’ve adjusted this sentence accordingly (ti. 118– 120).

R1.CJ Line 121: Clarify what “would have been” means? Is there better wording that could be used here?

Thanks for pointing this out. The multiple “would haves” do make this sentence hard to read. In the revision, I chose clearer and better wording (``. 133–136).

R1.CK Line 124: Clarify that the regression coefficient signifies the estimated transport.

The regression coefficient doesn’t signify transport. It corresponds to the change in transport per unit change in the sea-level difference across Florida Straits. This meaning is clear from the revised sentence immediately following this line, beginning, “This estimated change in transport per unit change in sea-level dif­ference .. . ” (``. 139–140). This line also references the methods section, where a detailed discussion of the regression coefficient and its physical interpretation is given (``. 769–785). This text together makes the clarification suggested by the reviewer.

R1.CL Line 136: “more exponentially” or “takes a more exponential form”? The latter. I’ve adopted the reviewer’s suggested wording here (``. 149–152).

R1.CM Line 142: statistical coherence and admittance or coherence and ad­mittance statistics.

I don’t understand the distinction the reviewer makes. To clarify what analyses are performed, I now reference Emery and Thomson (2014) on these points. I

5

also clarify that admittance is sometimes called the transfer function (``. 158– 162).

R1.CN Line 160: Why this interpretation? Are there other interpretations that might be valid?

This interpretation is justified by the analyses of GPS records, proxy sea-level reconstructions, and satellite altimetry described in what immediately follows this sentence. I’ve adjusted the revised text, so that there’s a smoother tran­sition between paragraphs, allowing this justification to be more apparent (ti. 175–186). Given the nature of and consistency between the various estimates and data, it’s hard to imagine a viable yet dramatically different alternative in­terpretation. Nevertheless, the uncertainties are large, and there’s clearly work to do in terms of reducing those uncertainties. Therefore, I’ve added new sen­tences into the revised text that point to concrete directions of future work (ti. 339–364).

R1.CO Line 170: Rate of rise? Be careful with consistency of terms. In line 167 you refer to this as rise and it could be unclear that you are referring to the same thing here.

I’ve changed this to “rate of sea-level rise” in line with the reviewer’s suggestion (``. 197–198).

R1.CP Line 175–178: Why not use the estimates from the model referenced in line 174?

Great question. I didn’t use those model results because they appear to conflict. Khan et al. (2017) estimate that southeastern Florida sea level rose by 0.7±0.3 mm y−' over the late Holocene (their section 3.10), whereas Love et al. (2016) estimate that sea level in this region over this period rose by 0.3–0.4 mm y−' (their Table 1). It’s unclear whether these values are significantly different, since Love et al.’s errors are not normally distributed, and Khan et al. never specify what the ± error ranges from their model represent. Also, since they apply different models to distinct but overlapping data sets, it’s unclear what the origins of any differences between their results are (e.g., data selection or model assumptions or both).

This prompted me to analyze the sea-level index points myself. Since the proxies aren’t the main focus, and are used to interpret the Bayesian model solutions, I use a simple but transparent analysis method (``. 202–211). In the end, I get similar numbers to Khan et al. (2017) for the Bahamas (my 0.8 ± 0.2 mm y−' versus their 0.9 ± 0.2 mm y−') and to Love et al. (2016) for southeastern Florida (my 0.2 ± 0.6 mm y−' versus their best estimates of 0.3–0.4 mm y−'). Similar rates of late-Holocene sea-level rise at other sites in Florida were also found by Kemp et al. (2014) and Gerlach et al. (2017). These are technical points, which would be tangential and distracting to the paper, and so I haven’t

6

altered the original text on these points, since it was sufficiently clear for my purposes.

**R1.CQ** Line 182: “furnished by”, word choice

I’ve removed the words “furnished by” so that these lines are clearer (11?. 209– 211).

**R1.CR** Line 194–195: Explain/clarify the significance and meaning.

I’ve included a new section in the Supplementary Information that explains how and why residual autocorrelation is accounted for in these trend error estimates (``. 16–35 in the revised Supplementary Information).

**R1.CS** Line 209: Clarify: Is weaker northward equivalent to stronger southward or are you trying to say that there are different directions in the trends?

No, “weaker northward” and “stronger southward” are not the same. The “weak” and “strong” descriptors refer to the magnitude and significance of the trends (e.g., stronger means larger and more significant). The “north” and “south” descriptors refer to the sense (or direction or sign) of the trends. The meaning here is made clear by the text and Figure 6, and so I haven’t made any changes to the manuscript on these points.

**R1.CT** Line 211: Clarify what adjusted for residual autocorrelations means. Please see my response to **R1.CR**, which addresses the reviewer’s question here.

**R1.CU** Figure 1: It is difficult to differentiate between the purple and red. Pos­sibly choose a different color for one or the other. A bit hard to read “Northwest Providence” and “Yucatan” in black.

Thanks for pointing this out. I’ve adjusted this figure so that the various lines are more easily differentiated and the text more readable.

**R1.CV** Figure 4: What type of interpolation. Is the standard deviation cal­culated from all models (from the average of the models)? Assumptions that I don’t have the expert knowledge to assess concern:

1. Geostrophic balance
2. Other reasons that tide gauges need to be used cautiously, because they represent different signals (non-oceanographic and geostrophic impacts on sea level and transport). What are these and how are they accounted for?
3. Data assimilation for other tide gauge data for RSL signals.

7

There are lots of comments to respond to here:

- A linear scattered data interpolant is used. This is now clarified in the figure caption.
- I don’t understand what the reviewer means by, “the standard deviation calculated from all models,” and, “from the average of the models”. No models are used. The figure is based on observational data and that the standard deviation is taken across cruises. These details have been clarified in the text (``. 144–152) and Figure 5 caption.
- Geostrophy is the dominant momentum balance across the Florida Straits on timescales 1 day, and so is a very good assumption on the timescales studied here. This has been clarified in the main text (``. 54–58, 754–755).
- In the revised text, I now give a more exhaustive list of effects other than geostrophic balance that could affect sea level (``. 59–63). In the meth­ods, I describe how the transport is modeled, and how non-oceanographic and ageostrophic processes are parameterized (ti. 769–785). The resid­ual analysis demonstrates that the model equations are appropriate given the data (``. 880–941), and the synthetic data experiments demonstrate that the Bayesian algorithm successfully infers the transport process (with meaningful error bars) given the available data (``. 980–1103).
- The comment “data assimilation for other tide gauge data for RSL signals” is unclear and therefore I cannot address it.

Detailed comments/questions, line-by-line in the methods and supplementary: Methods:

**R1.CW** Might there be a negative correlation in sea-surface height between the two regions in equation 3? Did you examine this possibility?

In designing the Bayesian model, I considered this possibility, but found that sea-level fluctuations between the two regions are generally uncorrelated or only weakly positively correlated. This can be seen in the positive-definite covariance matrices shown in Supplementary Figure 18. The spatial covariance structure assumed in the Bayesian model is largely consistent with that exhibited by the available observations. This has been clarified in the method section (``. 911– 920), and is consistent with previous studies (e.g., Thompson and Mitchum, 2014).

**R1.CX** Line 495: Define X.

X is now clearly defined as any positive integer (ti. 734–736).

8

R1.CY In line 489, you suggest that the longterm sea-level change are unrelated to ocean dynamics. However, the dynamic component of the difference in sea-level trends includes a term that is the opposite of the dynamic component (*α/ρ*). Please explain.

I don’t suggest that longterm sea-level trends are unrelated to ocean dynamics. The text states that the sea-level trends are potentially influenced by *both* ocean dynamical processes *and* processes unrelated to ocean circulation, and that these different effects need to be carefully separated in order to estimate trends in the Florida Current from tide-gauge records. The revised methods section has been expanded and now gives a fuller discussion of these points (*££*. 754–785).

R1.CZ Line 494: Unclear referential “this interpretation.” Does the interpre­tation refer to the relationship between dynamic and static terms?

I agree that this referential is unclear. I’ve clarified this sentence in the revised methods section (*££*. 777–782).

R1.CAA Line 500: You need to define *N*, *Mk* and *M*. I believe you mean that *Mk* is the locations where there are tide gauges at time step *k*, *M* is the total locations of any tide gauges, and *N* is the total locations for any measurement or any prediction?

*N*, *Mk* and *M* are all now defined in the revised methods (*££*. 725–729, 787–789, 793–794).

R1.CBB Line 508: a and *£* are always selected jointly. Can the selection matrices *H* and *F* have both terms for a single tide gauge? Please clarify.

Yes, both are selected jointly and are defined at each tide gauge. These two parameters play different roles in the model, and have different units: *£* is a random vector of bias offsets with units of length, while a is a random vector of error trends with units of length per time. Posterior solutions are well con­strained (Supplementary Figure 20), and there are no issues of identifiability. It’s unclear what the reviewer means by *H* and *F* “having both terms”; only *F* is applied to these parameters. These points have been made clear in the methods section (*££*. 791–797).

R1.CCC Line 505: My understanding is that *M* is the number of tide gauge locations and *N* is the total number of locations (including the tide gauge loca­tions). Why would the coastal sea level term selected by *H* ever not be selected? Please clarify?

The reviewer understands *M* and *N* correctly. The sea-level process is defined at all locations in space and time, whereas tide-gauge observations are made only at some places and some times. The selection matrix *Hk* accounts for this

9

difference, selecting the process at only the locations where observations were made at time step k. Thus, the sea-level process isn’t selected if there was no corresponding tide-gauge measurement at that point in space and time. These points have been made clear in the methods text (££. 795–797).

R1.CDD Line 612: Does arbitrary mean randomly selected?

Yes, arbitrary means randomly selected. So that there’s no confusion, I’ve indicated this meaning in the revised text (££. 890–892).

Supplementary:

R1.CEE Table S5: Did you set bounds on the hyperparameters? Clarify why the shape parameters of all of the inverse gamma distributions are the same.

I don’t understand what’s meant by setting bounds on the hyperparameters. However, I agree that I should have given more details on the overall philosophy and strategy for selecting the hyperparameter values. In the revised methods section, I now give more details, including an explanation for why the shape parameters on all the Gamma distributions are set to one-half (££. 805–813).

R1.CFF In some places you specify solutions in cm, some in km, and some in mm.

That’s correct. Some parameters are specified in mm, some in cm, some in m, and others in km. The reason for this is that the various parameters have different characteristic orders of magnitude. For a given parameter, I make a choice that is natural for the order of magnitude, and largely keep to that choice. For example, it is natural to express the inverse range parameters and A in units of km, whereas it is sensible to express the parameters related to the bias offset vector £ (v and r^2^) in units of m, given the respective orders of magnitude.

R1.CGG You don’t seem to reference some of the supplementary tables and figures within the supplementary text or the main text. Check to make sure you cover each of those.

Each figure and table is referenced at least once in the main text, methods, or supplementary information.

R1.CHH Line 14: “I formulate a simple ocean heat budget.” “volume is taken to be the full-depth”: Does that mean your calculation of volume includes the full depth? A depth is not a volume. Also, the Bering Strait is not part of the Atlantic Ocean. Are you developing a heat budget for the global oceans or just the Atlantic?

Good questions. It’s now apparent to me that some details of the heat bud­get weren’t clear in the original submission. I’ve overhauled this section, and

10

have tried to be clearer and more methodical (l?l?. 67–202 in the revised Supple­mentary Information). Hopefully these changes address the reviewer’s concerns here.

R1.CII Line 18: weakening of both the Florida Current AND the deep over­turning circulation? It is a balance between those two and turbulent ocean heat gain? Just need to clarify this sentence

Please see my response to R1.CHH above. R1.CJJ Line 25: word choice, throughout? In?

I’m not sure what word the reviewer is suggesting I replace with “throughout” or “in.” In any case, I think this point is moot, since I’ve substantially reworked this portion of the supplementary information.

R1.CKK S6: Clarify what you are doing here, please. These do not seem like equivalent equations.

Please see my response to R1.CHH above.

R1.CLL Use the same wording to define _Q_*_surf_* and _Q_*_moc_* as you use in lines 18–20 (or in lines 18–20 note that these two variables are _Q_*_surf_* and Q*moc*.

Please see my response to R1.CHH above.

R1.CMM Line 40: maybe use un-stippled instead of unhatched because of your wording in the figure caption?

Good catch on my inconsistent terminology. The word “unhatched” is no longer used.

R1.CNN Line 42: are these ranges or differences? Make sure that is clear and why you are using them.

In the revision, I clarify that these are indeed ranges, and also explain why they were chosen (l?l?. 159–161 in the revised Supplementary Information).

R1.COO Line 48: you can call this Figure S1(b), since that is what it is called in the figure. (same in line 81)

Good catch on my inconsistent figure referencing. In the revised text, I now indicate supplementary figures and tables in a consistent manner in line with journal style.

R1.CPP Line 126: I thought I would see the basis for these estimates in the supplementary

11

The basis for the error estimates on the cable-based annual transports is now explained in the methods (``. 702–707).

R1.CQQ Line 144: Doesn’t this prove that having less data creates larger posterior solution uncertainty? And the model solutions are in fact sensitive to this choice?

The question isn’t whether the model solutions are sensitive, but how sensitive they are to this choice. The text acknowledges that there are some differences, namely that when transport data are available, transport errors in the double-error experiment are twice as large as in the baseline experiment. However, by and large, the two solutions are nearly identical in terms of most quantities of interest (transport prior to 1982; centennial trend; transport change per unit change in sea-level difference across Florida Straits; Supplementary Figure 21). So, I believe that my original wording, that the two solutions are “generally quantitatively insensitive,” is appropriate and accurately conveys the results, and I haven’t changed it in the revision.

R1.CRR S13: It is difficult to see the baseline in each of these panels. Could you make the half data and double error transparent to reveal the baseline better?

I’ve adjusted all histogram figures so that all values are more clearly seen. R1.CSS Line 149: These are the medians and the 95% CIs, right?

Unless indicated otherwise, x ± y values represent the median plus and minus twice the standard deviation from the Bayesian model solution, which roughly corresponds to the 95% posterior credible interval. This meaning has been made clear in the text (``. 88–90).

R1.CTT Line 162: I assume you randomly chose the data to withhold, so this would be true with any data that was removed?

No, the withheld data weren’t chosen randomly. This experiment is meant to quantify the influence of a shorter data record. I withhold all data for the first half of the data record (1982–1998) but provide the algorithm with all data for the second half of the data record (2000–2018). Note that there were no data in 1999. These points have been clearly communicated in the text (``. 949–953). It’s unclear how similar or not the results would be in the distinct experiment wherein half of the data record was withheld at random, as the reviewer describes. Since it wouldn’t add anything meaningful to the manuscript, I haven’t performed such an additional experiment.

R1.CUU Line 174: Are you choosing the median posterior model solution or a selection within some CI of solutions? Shouldn’t you assume that the

12

uncertainty in the posterior matters for the generation of the true values or is there no uncertainty in the processes? Not just the data-level equations to perturb the process?

I don’t use the median posterior solution for the parameters. I use the ensemble member from the original baseline Bayesian model solution that minimizes the Mahalanobis distance to the mean parameter array over all ensemble members. This detail is clarified in the text (ti. 990–993). However, either subjective choice (median or ensemble member minimizing the Mahalanobis distance to the mean) would be reasonable in this context.

The reviewer is correct that my process and parameter estimates are uncertain. However, those uncertainties are not of utmost importance for generating the known process in the synthetic data experiments. The goal is to quantify the Bayesian model’s ability to infer a process that looks like the Florida Current transport given data that looks like the cable measurements and tide-gauge records. Thus, producing the synthetic process for these experiments doesn’t require that I perfectly know the “true transport” in any grand sense. Rather, it requires only that I perfectly know some process that is reasonable, in that its basic statistics (e.g., timescales and amplitudes of variability) are similar to the Florida Current transport. This criterion is satisfied by the experiments.

I’ll admit that a potential shortcoming is that I’ve only performed a small num­ber of experiments. How sensitive are my results to this small sample size and the particular parameter values and models I’ve chosen to base the experiments on? (I suspect this is what the reviewer’s really after here.) It’s impossible to answer these questions without performing a large number of synthetic data experiments. Unfortunately, running a meaningfully large ensemble of synthetic data experiments (e.g., order of hundreds) is unrealistic and unfeasible, given the computational cost of running a single experiment (order of hours). This shortcoming notwithstanding, the synthetic data experiments I’ve provided are still informative for demonstrating the reasonableness of the posterior solutions. Therefore, I believe that no adjustments to the manuscript are needed on these points, especially since many papers reconstructing transports provide no such demonstration whatsoever of the meaningfulness of the error estimates provided with their reconstruction.

**R1.CVV** Figure S14: Is the Bayes Sea-level solution the original solution, from which you generated the synthetic process and synthetic data? Or is it the posterior solution after running the model on the synthetic data? I am assuming the latter, but I would also like to see the former.

This comment refers to what is now Supplementary Figure 22. The top panels show three quantities at two locations. Black is the synthetic sea-level process, which was withheld from the Bayesian model. It was produced using data-constrained parameters from the original Bayesian model solution discussed in

13

the main text (Supplementary Table 6). Red is the synthetic tide-gauge record. It was determined by corrupting the synthetic process, and is available only some of the time. Blue is the posterior solution determined by applying the Bayesian model to the synthetic data at all locations. These details have been clarified in the text (``. 990–1007) and Supplementary Figure 22 caption.

I don’t show or discuss the original Bayesian model solutions for the sea-level process requested by the reviewer for two main reasons. First, doing so would be uninformative in the context of the synthetic data experiments. The process-and data-level equations model the sea-level field and tide-gauge data using random-in-time innovation series and error sequences. Therefore, while the synthetic process and data will have the same covariance amplitudes as in reality, the phasing will be random. For example, there is no reason for the long­term trend or short-term variation in sea level that actually occurred at Port-au-Prince during the past 110 y to match the simulated trend or variation in Supplementary Figure 22a. That is, showing the original posterior solution in this figure would be comparing apples to oranges.

Second, doing so would be tangential and beyond the scope of the paper. My focus is on changes in the Florida Current. Discussion of sea-level solutions is restricted to those locations (Settlement Point, Virginia Key, Lake Worth Pier) that are close to the transport cable, and are helpful for interpreting the changes inferred in the Florida Current. It’s unclear what a more general discussion of the posterior sea-level solution would bring, other than making what is already a very long paper even longer. Once this paper is published, I plan to make the complete Bayesian model solution available through Github, which will enable a future study to interrogate the estimated sea-level changes.

**Reviewer 2**—

Review of Weakening of the Gulf Stream at Florida Straits over the past century inferred from coastal sea-level data, by Christopher G. Piecuch

This paper provides a thorough discussion of long-term changes in Florida Cur­rent transport over the past 110 years, and finds a weakening of this current over this period. This is a very interesting finding as the Florida Current is a key component of Atlantic Ocean circulation, linked to both the wind-driven circulation and the thermohaline overturning circulation.

Let me first state that I approach this review with my general expertise on the AMOC, but I do not have experience with the Bayesian model used by the author. I therefore recommend that someone with expertise in this methodology reviews this paper as well, if this hasn’t happened already.

The discussion of the causes of the declining trend is very interesting, where the key question is whether the decline is linked to wind changes or to thermohaline overturning changes. Unfortunately, reanalysis data are inconsistent and thus

14

don’t allow to determine even the sign of the wind contribution. On the other hand, the paper provides a plausible estimate of the overturning contribution, arguing that the data are consistent with this largely explaining the declining trend of the Florida Current.

I think this is an important study which I recommend for publication in Nature Communications after minor revisions.

I appreciate the reviewer’s positive assessment. Below I describe revisions made to the paper in response to the reviewer’s comments.

R2.CA Primarily, I would suggest to the author to strengthen the section “Relation to the Deep Overturning” by adding a discussion not only in terms of heat budget consistency but also regarding consistency with transport changes in Sv estimated in previous studies. Given a total Florida Current transport of 32 Sv and a gyre estimate of 23 Sv, this would leave only 9 Sv for overturning, which is less than the total overturning (estimated to be more like 15-20 Sv). Presumably the missing part is flowing northward east of Grand Bahama and thus not part of the Florida Current. What does this mean for the estimate in trend? how much of an AMOC weakening trend would show up in the Florida Current, and how much to the east of Grand Bahama? This issue needs a bit of discussion (even if it can’t be resolved).

I agree that these points deserve much more attention than I gave them in the original submission. Therefore, I substantially expanded the part of the main text that discusses the weakening of the Florida Current in the context of other possible circulation changes along 27^◦^N (``. 231–325). This section includes a comparison between my estimate of the mean southward gyre transport and previous estimates (ti. 240–243) as well as a discussion of possible changes in the Antilles Current transport east of Abaco (``. 259–274), which were both requested by the reviewer. It also includes a more general heat and mass budget exercise, wherein I put bounds on possible changes in the deep branch of the overturning circulation and the upper-ocean thermocline recirculation given the estimated changes in Florida Current transport and observed cooling of the subpolar sea surface (``. 275–325).

Small comments:

R2.CB The author could also cite some further proxy data studies indicating a long-term overturning decline, e.g. Thibodeau et al. 2018, Zanna et al. 2019.

Thanks for the references. I’ve added those two and others (e.g., the recent review by Moffa-Sánchez et al., 2019).

R2.CC Line 45: “deep overturning circulation” usually denotes the full “over­turning”, i.e. both upper northward and deep southward branch. I suggest to replace with “deep branch of the overturning circulation”.

15

Agreed. I’ve changed the text accordingly throughout. I no longer use “deep overturning circulation” but rather “deep branch of the overturning circulation” or “deep return flow of the overturning circulation.”

**R2.CD** Line 82: Is it statistically or conceptually better to compare the period after 1982 to the mean of the entire record, or to the mean of the time preceding 1982? I find it more intuitive to compare two non-overlapping periods, e.g. because the difference then corresponds to how much the current has weakened over time. I also find the wording “that the cable data probably represent the Florida Current in a reduced state of transport” strangely vague. Does it mean that according to the cable data, the Florida Current has probably been weaker since 1982 than before? If so, say so. If not, why not?

Sorry for the confusion here. While there’s no advantage statistically one way or the other, I’ll grant that it’s more straightforward conceptually to compare the two non-overlapping periods 1909–1981 and 1982–2018, which I now do in the revision (``. 90–93; Supplementary Figure 3). I’ve also clarified the text to more clearly communicate that Florida Current transport has probably been weaker since 1982 than before (``. 11–12, 330–333). See also my reply to **R1.CA** above.

**R2.CE** Line 103: AMV/AMO: The reality of this variability as a distinct mode is highly questionable, see e.g. Mann, M. E., Steinman, B. A. & Miller, S. K. Absence of internal multidecadal and interdecadal oscillations in climate model simulations. Nat Commun 11, 49 (2020) which states: “Here we use a com­bination of observational data and state-of-the-art forced and control climate model simulations to demonstrate the absence of consistent evidence for decadal or longer-term internal oscillatory signals that are distinguishable from climatic noise.”

Thanks for this reference. I hadn’t seen it yet. The Mann et al. (2020) findings don’t contradict anything in my paper. Those authors don’t argue that the AMV isn’t a bonafide physical mode of climate variation. Rather, they call into question whether AMV is a true oscillation in the sense of having a well-defined, narrow-band spectral peak. However, whether AMV is distinguishable from climatic noise really has no bearing on my results, and so I haven’t altered the manuscript on this point.

**Reviewer 3**—

This paper tests a hypothesis that the flow through the Florida Strait has weak­ened during the last century. This is a topical and important question. Several studies (cited in the manuscript), using proxy data, have concluded that the Atlantic Meridional Overturning Circulation (AMOC) has weakened but there is as yet no clear consensus. Thus, there will be much interest in this paper amongst those who study Atlantic circulation and its impact on climate. The

16

probabilistic method employed seems to be a good choice to extract information from the many incomplete time series and overall the paper is well written. I recommend that the paper be published, but have a few questions that could perhaps lead to some improvements in the paper.

Thanks, I appreciate the reviewer’s positive review. Below, I provide answers to the reviewer’s questions, which lead to improvements in the paper.

**R3.CA** I found it curious that the results highlight that the AMOC has declined since the 1970s. The 1970s and 1980s was a time when the North Atlantic was relatively cool compared to the following decades, and the AMV index had low values. Figure 6 and the associated discussion suggest the century long cooling trend in the sub polar gyre and the reduction of the Florida Current are consistent. But are the decadal variations of these quantities also consistent?

Great questions. The original paper argued that (1.) Florida Current transport declined over various periods, including since the 1970s, and (b.) the 110-y decline in Florida Current transport is consistent with observed cooling of sub­polar sea-surface temperatures and hypothesized weakening of the deep branch of the overturning circulation. However, in the original paper, I didn’t consider the link between the Florida Current and the overturning circulation at other timescales. Therefore, it wasn’t clear whether my original results support a weakening of the deep return flow of the overturning circulation since the 1970s.

In the revision, I now consider the relationship between the Florida Current and other circulations across 27^◦^N more generally across timescales using sim­ple heat and mass conservation principles. I find that estimated changes in the Florida Current, when viewed in light of observed changes in subpolar sea-surface temperatures, support a weakening of the deep branch of the overturning circulation during the past 70–110 y or so. However, I also find that on shorter timescales (e.g., since the 1970s), a meaningful decline in the overturning circu­lation cannot be detected, given these estimates and principles (``. 299–308). I appreciate the reviewer prompting me to dig deeper into these issues.

**R3.CB** I wonder if it would be better to merge the two sections “Relation to Wind Stress and the Interior Gyre” and “Relation to the Deep Overturning”? These are distinct from the rest of the paper and explore the implications of the likely slowdown in the Florida Current. It is stated that “weakening of the Florida Current transport must have been balanced by changes in the interior gyre or overturning transports at 27N”. However, there is a third possibility, alluded to in the conclusions, that the reduction is balanced by an increase in transport in the Antilles Current so that the total western boundary current is unchanged. I think it would be better to mention this explicitly at the start of this section.

Excellent suggestions. Following the reviewer’s recommendations, I’ve combined these two sections in the revised manuscript (``. 231–325). I’ve also inserted

17

a new discussion of the Antilles Current (``. 259–274). These revisions im­mediately precede the expanded heat and mass budget discussions, described immediately above in response to **R3.CA**.

**R3.CC** I confess that I am not sufficiently familiar with the statistical tech­nique used to give an expert opinion the details of the analysis. However, the methodology is mostly well described, though I had to search for a definition of the term “innovation sequence”, and it would be better to define that in the text.

Reviewer 1 is an applied statistician who provided a thorough assessment of the methods. The reviewer’s point regarding innovation sequences is well taken, since this terminology will be foreign to most physical oceanographers and cli­mate scientists. In the revised methods section, I now provide a more infor­mative description, making an analogy to the forcing function of the classical stochastic climate model of Frankignoul and Hasselmann (1977) that will be more familiar to oceanographers and climate scientists (``. 730–732).

**R3.CD** I would like to see a spatial map of the expected sea-level correlation with transport anomaly that is deduced from the analysis. Is it possible to test with the data whether this relationship between sea-level and transport is stationary in time?

In response to the reviewer’s request, I’ve added a new supplementary figure showing the theoretical and empirical maps of the correlation coefficient between transport and local sea level along with accompanying text to the revised method section (``. 921–928; Supplementary Figure 19).

Given the available data, it’s impossible to make a meaningful observational test of whether or not the relationship is stationary on the timescales of interest to this study. However, it is possible to make such a test based on an ocean circula­tion model. Using output from SODA, I find that the relation between Florida Current transport and sea-level difference across Florida Straits doesn’t show strong dependence on time period or frequency band (``. 155–173, 1072–1088; Supplementary Figures 6, 7). On this basis, I conclude that the assumption of a stationary relationship is reasonable.

**R3.CE** Figure S7 gives an overview of the amount of data available and all the details are in the table S3, but I think it would be instructive to add to Figure S7 maps of the data available for some particular decades, e.g. 1920s, 1960s and 2000s.

This comment refers to what is now Supplementary Figure 1. I agree that such a map, showing data availability for different time periods, would be informative. I’ve now included just such a map in the revised manuscript (Supplementary Figure 2).

18

R3.CF The author is careful to highlight the uncertainties and to quantify the likelihood that the Florida Current has reduced. Although there is significant uncertainty about some results, they are still interesting and valuable, but I wonder if the title might be better as “Likely weakening ... ”

I agree that it’s important to emphasize uncertainties. I’ve added the word “likely” to the paper’s title, as the reviewer suggests. Note that I’ve also short­ened the title (e.g., changing ‘Gulf Stream at Florida Straits’ to ‘Florida Cur­rent’), to be consist with the journal’s maximum title length is 15 words.

References

- Beal, L. M., J. M. Hummon, E. Williams, O. B. Brown, W. Baringer, and E. J. Kearns. Five years of Florida Current structure and transport from the Royal Caribbean Cruise Ship *Explorer of the Seas*, *J. Geophys. Res.*, 113, C06001 (2008).
- Frankignoul, C., and K. Hasselmann. Stochastic climate models, part II: application to sea-surface temperature anomalies and thermocline vari­ability, *Tellus*, 29, 289–305 (1977).
- Garcia, R. F., and C. S. Meinen. Accuracy of Florida Current volume transport measurements at 27^°^N using multiple observational techniques, *J. Atmos. Ocean. Tech.*, 31, 1169–1180 (2014).
- Gerlach, M. J., S. E. Engelhart, A. C. Kemp, R. P. Moyer, J. M. Smoak, C. E. Bernhardt, and N. Cahill. Reconstructing Common Era relative sea-level change on the Gulf Coast of Florida, *Mar. Geol.*, 390, 254–269 (2017).
- Kanzow, T., S. A. Cunningham, D. Rayner, J. J.-M. Hirschi, W. E. Johns, M. O. Baringer, H. L. Bryden, L. M. Beal, C. S. Meinen, and J. Marotzke. Observed flow compensation associated with the MOC at 26.5^°^N in the Atlantic, *Science*, 317, 938–941 (2007)
- Kemp, A. C., C. E. Bernhardt, B. P. Horton, R. E. Kop, C. H. Vane, W.

R. Peltier, A. D. Hawkes, J. P. Donnelly, A. C. Parnell, and N. Cahill. Late Holocene sea- and land-level change on the U.S. southeastern Atlantic coast, *Mar. Geol.*, 357, 90–100 (2014)

- Khan, N. S., E. Ashe, B. P. Horton, A. Dutton, R. E. Kopp, G. Brocard,

S. E. Engelhart, D. F. Hill, W. R. Peltier, C. H. Vane, and F. N. Scatena. Drivers of Holocene sea-level change in the Caribbean, *Quat. Sci. Rev.*, 155, 13–36 (2017).

- Love, R., G. A. Milne, L. Tarasov, S. E. Engelhart, M. P. Hijma, K. Latychev, B. P. Horton, and T. E. Törnqvist. The contribution of glacial isostatic adjustment to projections of sea-level change along the Atlantic and Gulf coasts of North America, *Earth’s Future*, 4, 440–464 (2016).

19

- Mann, M. E., B. A. Steinman, and S. K. Miller. Absence of internal multidecadal and interdecadal oscillations in climate model simulations, *Nat. Common.*, 11, 49 (2020).
- Meinen, C. S., M. O. Baringer, and R. F. Garcia. Florida Current trans­port variability: an analysis of annual and longer-period signals, *Deep-Sea Res.*, 57, 835–846 (2010).
- Moffa-Sánchez, P., E. Moreno-Chamarro, D. J. Reynolds, P. Ortega, L. Cunningham, D. Swingedouw, D. E. Amrhein, J. Halfar, L. Jonkers, J. H. Jungclaus, K. Perner, A. Wanamaker, and S. Yeager. Variability in the northern North Atlantic and Arctic Oceans across the last two millennia: a review, *Paleoceanogr. Paleocl.*, 34, 1399–1436 (2019).
- Sheinbaum, J. J. Candela, A. Badan, and J. Ochoa. Flow structure and transport in the Yucatan Channel, *Geophys. Res. Lett.*, 29, 1040 (2002).
- Thibodeau, B., C. Not, J. Hu, A. Schmittner, D. Noone, C. Tabor, J. Zhang, and Z. Liu. Last century warming over the Canadian Atlantic shelves linked to weak Atlantic meridional overturning circulation, *Geo-phys. Res. Lett.*, 45, 12376–12385 (2018).
- Thompson, P. R., and G. T. Mitchum. Coherent sea level variability on the North Atlantic western boundary, *J. Geophys. Res. Oceans*, 119, 5676–5689, doi:10.1002/2014JC009999 (2014).
- Zanna, L., S. Khatiwala, J. M. Gregory, J. Ison, and P. Heimbach. Global reconstruction of historical ocean heat transport and transport, *Proc. Natl. Acad. Sci. U.S.A.*, 116(4), 1126–1131 (2019).

**Reviewer comments, second version:**

Reviewer #1 (Remarks to the Author):

The author has addressed all of the issues raised in my previous review. I think the paper will influence thinking in the field, and I find the statistical analysis valid and appropriate. I believe the work will be reproducible with the level of detail provided.

- Erica Ashe


Reviewer #2 (Remarks to the Author):

I am generally happy with the revisions.

I wouldn't use the term "temperature acceleration" (line 286), and perhaps the extended section "Relation to Large-Scale North Atlantic Ocean Circulation" can be made a bit easier to read generally.

2 Examples:
1. "trend in surface heat flux of 16 11 W m2 century􀀀1 over the warming hole"
Should make sign intuitively clear, e.g. "trend in surface heat uptake"
2. "trends in the deep return flow are positive (northward)" is confusing as a positive trend suggests in increase, but since this is a southward flow it is a decrease. Why not say: "trends show a decline in the deep return flow..."

Stefan Rahmstorf


Reviewer #3 (Remarks to the Author):

The author has addressed all of the comments from the previous review and I recommend publication of the revised manuscript.
David Smeed

**Author rebuttal, second version:**

**Reviewer 1**—

The author has addressed all of the issues raised in my previous review. I think the paper will influence thinking in the field, and I find the statistical analysis valid and appropriate. I believe the work will be reproducible with the level of detail provided.

No response needed.
**Reviewer 2**—

I am generally happy with the revisions. I wouldn’t use the term “tempera­ture acceleration” (line 286), and perhaps the extended section “Relation to Large-Scale North Atlantic Ocean Circulation” can be made a bit easier to read generally. Two examples:

1. “trend in surface heat flux of 16 ± 11 W m−^2^ century−^1^ over the warm­ing hole” should make sign intuitively clear, e.g., “trend in surface heat uptake”.
2. “trends in the deep return flow are positive (northward)” is confusing as a positive trend suggests in increase, but since this is a southward flow it is a decrease. Why not say: “trends show a decline in the deep return flow.. . ”

The term “temperature acceleration” is precise, clear, and readily understood. Since the reviewer doesn’t offer a suitable alternative, I’ve retained this phrase in the final manuscript version. In any case, the reviewer’s more general point that this section should be made more readable is well taken. I’ve combed through this section and made a few changes that make it easier to read. These changes are indicated in the tracked changes version of the manuscript.

**Reviewer 3**—

The author has addressed all of the comments from the previous review and I recommend publication of the revised manuscript.

No response needed.

2

**Reviewer comments, third version:**

**Author rebuttal, third version:**
